# Determination of sulfachloropyridazine residue levels in feathers from broiler chickens after oral administration using liquid chromatography coupled to tandem mass spectrometry

**DOI:** 10.1371/journal.pone.0200206

**Published:** 2018-07-05

**Authors:** Ekaterina Pokrant, Francisca Medina, Aldo Maddaleno, Betty San Martín, Javiera Cornejo

**Affiliations:** 1 Preventive Medicine Department, Faculty of Veterinary and Animal Sciences, University of Chile, La Pintana, Santiago, Chile; 2 Laboratory of Veterinary Pharmacology, Faculty of Veterinary and Animal Sciences, University of Chile, La Pintana, Santiago, Chile; University of Campinas, BRAZIL

## Abstract

Several antimicrobials are routinely used by the poultry farming industry on their daily operations, however, researchers have found for some antimicrobials that their residues persist for longer periods in feathers than they do in edible tissues, and at higher concentrations, as well. But this information is not known for other classes of antimicrobials, such as the sulfonamides. Therefore, this work presents an accurate and reliable analytical method for the detection of sulfachloropyridazine (SCP) in feathers and edible tissues from broiler chickens. This method was also validated in-house and then used to study the depletion of sulfachloropyridazine in those matrices. The experimental group comprised 54 broiler chickens, who were raised under controlled conditions and then treated with a commercial formulation of 10% sulfachloropyridazine for 5 days. Samples were analyzed via LC-MS/MS, using ^13^C_6_-sulfamethazine (SMZ-^13^C_6_) as an internal standard. Aromatic sulfonic acid solid phase extraction (SPE) cartridges were used to clean up the samples. The Limit of Detection (LOD) for this method was set at 10 μg kg^-1^ on feathers and liver; and at 5 μg kg^-1^ on muscle. Within the range of 10–100 μg kg^-1^, the calibration curves for all matrices presented a determination coefficient greater than 0.96. Our results show, with a 95% confidence level, that sulfachloropyridazine persisted in feathers for up to 55 days after ceasing treatment, and its concentrations were higher than in edible tissues. In consequence, to avoid re-entry of antimicrobial residues into the food-chain, we recommend monitoring and inspecting animal diets that contain feather derivatives, such as feathers meals, because they could be sourced from birds that might have been medicated with sulfachloropyridazine.

## Introduction

Food security, safety, and affordability are important concerns nowadays, and these qualities are strongly dependent on the ability to raise healthy animals. In turn, ensuring animal health occasionally requires using antimicrobials to treat their diseases [1, 2]. However, using antimicrobials in food animals could lead to unintended consequences, such as the development of resistant microorganisms, or a greater probability of finding drug residues in edible tissues or other animal by-products [3, 4]. For consumers, these drug residues may cause hypersensitivity, tissue damage, gastrointestinal alterations and neurological disorders [5–7].

The sulfonamides class of antimicrobials is used in veterinary practice for the therapy of infectious diseases. Most sulfonamides are quickly absorbed at the gastrointestinal level and then distributed to body tissues and fluids (e.g. synovial and cerebrospinal fluids). These molecules bind to plasma proteins at levels ranging from 15% to 90% where binding levels that are higher than 80%result in a greater half-life [8]. In this regard, is interesting how Baert et al. [9] found that when administering one dose of 33.34 mg kg^-1^ of a sulfadiazine formulation to broiler chicken, its oral bioavailability reached an 80%. Meanwhile, Sentepe and Eraslan [10] calculated that following one oral dose of 60 mg kg^-1^ of sulfaclozine to broiler chickens, its bioavailability only reached 49.93%.

Within the sulfonamides group, molecules differ by which radical is bound to the amide group (-SO_2_NHR) or, occasionally, by the substituent amide group (-NH_2_). These variations confer each molecule their characteristic physicochemical and pharmacokinetic properties, as well as their antimicrobial activity profile. Sulfachloropyridazine, along with sulfamethoxazole and sulfadiazine, are deemed as the sulfonamides that better resist biodegradation [11].

The broad-spectrum activity of sulfonamides against bacteria and protozoa, as well as their low cost, are the main drivers for their use in poultry farming to treat infections of the digestive and respiratory tracts [12, 13]. However, considering the risk that the presence of antimicrobial drug residues in animal products poses to public health, both the European Union (EU) and the Food and Agriculture Organization (FAO) have set Maximum Residue Limits (MRL) in all edible tissues. In the case of sulfonamides, the EU established an MRL of 100 μg kg^-1^ [14], whereas FAO established the same MRL for sulfamethazine [15].

Currently, several studies have analyzed the pharmacological behavior of antimicrobials belonging to the family of sulfonamides, on different edible tissues of poultry. For example, Łebkowska-Wieruszewska and Kowalski [16], as well as Li and Bu [17], determined sulfachloropyrazine in muscle, liver, skin and fat of broiler chickens, after receiving medicated feed. These authors concluded that this drug reached high concentrations up to day 4 after ceasing treatment, before it quickly declined to levels below the limit of detection for the analytical methodology. Furthermore, Łebkowska-Wieruszewska and Kowalski [18] had previously detected these analytes in samples of turkey muscle and liver tissues, finding that their concentrations decreased rapidly from day 7 on, after ceasing treatment. Additionally, Premarathne et al., [19] detected sulfadiazine concentrations that were higher that the detection capability of their method (138.1 μg kg^-1^), in three eggs samples from a total of 50 samples they analyzed.

Meanwhile, antimicrobial behavior in by-products such as feathers has already been studied for fluoroquinolones, amphenicols, macrolides and tetracyclines [20–27]. Interestingly, researchers have found higher concentrations in feathers than in edible tissues such as liver and muscle samples. Therefore, it is clear that feathers are a matrix that bio-accumulates antimicrobial residues. Nowadays though, neither presence of sulfonamide residues nor their behavior, has been assessed in poultry by-products. A step in that direction is the work of Jansen et al. [28], who developed a multi-residue method based on using ultra-high performance liquid chromatography (UHPLC) coupled to tandem mass spectrometry (MS/MS) for the analysis of chicken feathers. That method allowed them to perform qualitative confirmatory analyses of tetracyclines, quinolones, macrolides, lincosamides, pleuromutilins and sulfonamides. However, no studies have been done yet in chicken feathers to analyze the behavior and depletion of sulfonamides, establishing a parallel to their concentration in edible tissues.

Feathers are an especially important among by-products of the poultry industry, considering that it accounts for approximately 4 to 9 percent of the live weight of each bird [29]. Unsurprisingly, poultry rendering plants produce vast amounts of feather meal. For example, in the year 2008, 603.9 metric tons of feather meal were produced in the USA, and 73.3 metric tons were then exported [30].

Feather meal is a specially interesting source of protein for animal diets because keratin accounts for more than 90% of the chemical composition of feathers [31]. Feather meal is also regarded as a cheap source of protein for diets of poultry, swine, ruminants and fish. This means that feather meal ultimately becomes part of the food-chain [21, 32, 33]. But this situation poses a risk to human and animal health, as it is a path of re-entry into the food chain for multiple drugs and contaminants, and it has not been properly accounted for yet. Love et al. [32] made this clear when they analyzed feather meals sampled in China and several USA States (Arkansas, North Carolina, Oregon, California, Idaho and Tennessee), detecting 17 antimicrobials from six different families.

Taking into account the role of feather meal as well as the high volume that rendering plants are producing, it is important to determine how other antimicrobial families behave in this matrix. That information would allow designing mitigation and control measures for antimicrobial residues when they are detected in this non-edible by-product.

The work of Jansen et al. [28] brings that goal one step closer, because these authors have developed a multi-residue method based on ultra-high performance liquid chromatography (UHPLC) coupled to tandem mass spectrometry (MS/MS) for the analysis of chicken feathers. Such method allowed them to perform qualitative confirmatory analyses of tetracyclines, quinolones, macrolides, lincosamides, pleuromutilins and sulfonamides. However, as noticed before, no studies have analyzed the behavior and depletion of sulfonamides in chicken feathers while also relating those results to the concentrations of these antimicrobials that could be found in edible tissues.

Because of the reasons mentioned above, in this work we have determined the depletion time for sulfachloropyridazine (SCP) in feathers from broiler chickens who received a commercial formulation of 10% SCP. We also compared the concentrations found in these by-products with those detected in edible tissues that were sampled from the same birds.

## Materials and methods

### Controlled treatment study

The experimental animal population totaled 54 1-day-old male broiler chickens from the genetic line Ross 308. These birds were sourced from a commercial hatchery and raised in batteries that were subject to controlled environmental conditions (25 ± 5°C and 50–60% relative humidity). They were provided *ad libitum* access to water and non-medicated feed. The latter was analyzed to ensure it was not contaminated with antimicrobial drugs. This feed was formulated according to recommendations from the genetic company, using mainly corn and soy beans as the basic ingredients. Regarding their cages, these had a raised wire-mesh floor to avoid fecal contamination of feathers.

These birds were raised and monitored within indoor facilities of the Avian Pathology Laboratory from the Faculty of Veterinary and Animal Sciences of the University of Chile. Both their housing and slaughter protocol were approved by the Bioethics Committee of the Faculty of Veterinary and Animal Sciences (Certificate N° 23–2014). These were carried out in strict accordance with the recommendations stated in the Animal Protection Act N° 20,380 of the Chilean legislation [34], as well as the Directive 2010/63/EU ‘on the protection of animals used for scientific purposes’ [35]. Additionally, the slaughter protocol was designed in conformity with the Council Regulation (EC) No. 1099/2009 ‘on the protection of animals at the time of killing’ [36].

The depletion study was designed following the recommendations of the European Medicines Agency on its ‘Guideline on approach towards harmonization of withdrawal periods’ EMA/CVMP/SWP/735325/2012 [37]. The experimental design established two groups of chicks (A and B) that were randomly regrouped on their fifth day of life. Group ‘A’ comprised 42 chickens, who were treated with a commercial formulation of 10% sulfachloropyridazine via an orogastric catheter to ensure delivering a full dose of the drug. The therapeutic dose was set at 30 mg kg^-1^ and it was delivered once a day over five consecutive days. The label of this product established a withdrawal time of 30 days for chicken muscle. Meanwhile, group ‘B’ comprised 12 chickens that were kept as untreated control animals.

Seven sampling points were set at days 7, 14, 21, 32, 36 and 38 after ceasing treatment. On each occasion, seven birds from group ‘A’ and two birds from group ‘B’ were slaughtered.

### Sample collection and processing

Muscle, liver and feather samples were collected from every slaughtered bird at each sampling point. From each bird, samples included sections of breast and leg muscles, their entire liver, and all of their feathers. In the case of muscle and liver samples, the first step to process them was the removal of fats and grinding. Meanwhile, feather samples were first treated cryogenically with liquid nitrogen, as different studies have reported that this procedure improves grinding efficiency of feathers [23, 25, 26]. Then, samples were ground in an industrial Robot Coupe^®^ R4 table-top cutter food processor (Burgundy, France) to ensure their homogeneity. Finally, all samples were individually packaged and stored at -20°C until needed for analyte extraction and chromatographic analysis.

### Implementation of analytical methodology

Analytical methodologies for the detection of sulfachloropyridazine (SCP) in samples of feathers, muscle and liver tissue via LC-MS/MS were implemented based on techniques previously published by other authors [38–44].

#### Standards, reagents and solutions

Sulfachloropyridazine (SCP) of certified purity (99.7%) was the standard used in this work. An intermediate spiking solution was prepared then, dissolving the SCP standard in HPLC-grade water up to a concentration of 500 ng mL^-1^, and used to fortify blank samples. Also, Sulfamethazine-phenyl-^13^C_6_-hemihydrate (SMZ-^13^C_6_) of certified purity (99.9%) was used to prepare an internal standard (IS) by diluting SMZ-^13^C_6_ in HPLC-grade water up to a concentration of 115.5 ng mL^-1^. All reagents were manufactured by Sigma Aldrich, Inc. (Merck KGaA).

Reagents such as water (HPLC grade), formic acid (98–100%, HPLC grade), hydrochloric acid (P. A. grade), n-Hexano (HPLC grade), ammonia solution 25% (P. A. grade), hydrochloric acid fuming 37% (P. A. grade), and sodium hydroxide (P.A. grade), were all sourced from Merck Millipore (Merck KGaA), while methanol and ethyl acetate (HPLC grade) were sourced from J.T. Baker^®^ (Avantor^™^ Performance Materials, LLC).

Two solutions (mobile phase A and B) were used for extraction. Mobile phase A was 0.1% formic acid in methanol (pH 2.9 ± 0.3) and mobile phase B was 0.1% formic acid in water (pH 2.7 ± 0.2).

#### Extraction of sulfachloropyridazine from feather samples

Extraction began by weighing in 2 ± 0.02 g of each sample in a 50-mL polypropylene tube and then fortifying it with the internal standard solution (SMZ-^13^C_6_). Samples rested for 15 minutes before adding 40 mL of ethyl acetate to the mix. The resulting mixture was stirred for 15 minutes on a vortex-mixer and then sonicated for 5 minutes. Next, feathers were centrifuged at 1,800 g for 10 minutes and the supernatant was filtered through glass wool into a fresh 50-mL polypropylene tube where they were concentrated down to 15 mL using a water bath (40–50°C) under a mild nitrogen flow.

Aromatic sulfonic acid (BAKERBOND spe^™^) disposable extraction columns of 6 mL (column size) and 500 mg (sorbent weight) were used for solid phase extraction (SPE), conditioning them with 6 mL of hexane and 6 mL of ethyl acetate. Samples were filtered through the columns and then these were washed up with 2 mL of water and 2 mL of methanol. Elution was then performed with 10 mL of a mixture of methanol and ammonia solution (97/3) and the eluate was evaporated using a water bath (40–50°C) under a mild nitrogen flow. Samples were reconstituted in 300 μL of a mixture of mobile phase A and B (15/85), stirred on a vortex-mixer and sonicated for 5 minutes. This reconstituted solution was transferred into an Eppendorf tube and centrifuged at 17,000 g for 10 minutes. Finally, samples were filtered through 13 mm millex filters with 0.22 μm polyvinylidene fluoride (PVDF) membranes. These resulting filtrates were transferred into glass vials while waiting for LC-MS/MS injection and analysis (dx.doi.org/10.17504/protocols.io.pr4dm8w).

#### Extraction of sulfachloropyridazine from muscle and liver samples

The extraction process began by weighing of each sample within a 50-mL polypropylene tube (5 ± 0.05 g for muscle and 2 ± 0.02 g for liver), and then fortifying it using the internal standard solution (SMZ-^13^C_6_). Each sample rested for 15 minutes, 15 mL of water were added to the tube and then were stirred for 10 minutes on a vortex-mixer. Afterward,15 μL of NaOH 1M were added to the mixture, and samples were stirred again for 15 minutes, sonicated for 5 minutes, and their pH was adjusted to 7.8–8.0 with 10% v/v HCl.

Next, 10 mL of ethyl acetate were added to each sample, stirring them for 10 minutes on a vortex-mixer, and centrifuging them at 2,790 g for 2 minutes. The resulting gel was dissolved shaking the samples by hand in a flask, and centrifuging them again at 2,790 g for 10 minutes. The supernatant was transferred to a clean 50 mL polypropylene tube, and the last step was repeated twice. The resulting supernatant was evaporated using a water bath (40–50°C) under a mild nitrogen flow, followed by a reconstitution in 500 μL of a mixture of mobile phase A and B (15/85). Then, the solution was stirred on a vortex-mixer, sonicated for 5 minutes, transferred to an Eppendorf tube and centrifuged at 17,000 g for 10 minutes. Finally, samples were filtered through 13 mm millex filters with 0.22 μm polyvinylidene fluoride (PVDF) membranes. The filtrate was transferred to glass vials while waiting for LC-MS/MS injection and analysis (dx.doi.org/10.17504/protocols.io.pvcdn2w and dx.doi.org/10.17504/protocols.io.pwxdpfn).

#### LC-MS/MS analysis

For the instrumental analysis, a Symmetry C8 analytical column of 3.5μm and 2.1 x 100 mm (Waters^®^) was fitted in an Agilent 1290 Infinity Series liquid-chromatograph equipment, coupled to an API 3200 (AB Sciex, Darmstadt, Germany) triple-quadrupole mass-spectrometer. The analytical data was then integrated using the Analyst^®^ version 1.5 software package (SCIEX, Framingham, Massachusetts).

To achieve isocratic chromatographic separation, a flow gradient of 200 μL min^-1^ was established with a 45% of mobile phase A and a 55% of mobile phase B, which corresponded to extraction solutions A and B, respectively, as well as a volume of injection of 20 μL and a column oven temperature of 35 °C.

A multiple reaction monitoring (MRM) scan type was used for acquiring and visualizing LC-MS/MS data. The source temperature was 450°C, and the pressure was 40 psi for nebulizer (GS1), 20 psi for turbo ion (GS2), 20 psi for curtain gas and 10 psi for collision gas. The ionization was performed by electrospray, and the ion spray voltage was 5000 V. Table 1 lists the monitored ions masses.

**Table 1:** Monitored ions.

Analyte	Precursor ion (Q1^d^ mass) (Da^e^)	Fragment ion (Q3^f^ mass) (Da)	Time (ms)	DP^g^ (V^h^)	EP^i^ (V)	CE^j^ (V)	CXP^k^ (V)	RT^l^ (min)/CV (%)
**SCP 1**^a^	284.934	155.900	200.0	31.000	5.500	19.000	4.000	2.510 / 0.98
**SCP 2**^b^	284.934	108.200	200.0	31.000	5.500	27.000	10.000	2.503 / 0.93
**SMZ-**^**13**^**C**_**6**_ **(IS)**^c^	285.113	124.100	200.0	71.000	4.500	29.000	4.000	2.122 / 1.70

Monitored ions masses, voltage parameters, fractionation parameters and analyte retention time.

^a^SCP 1: Sulfachloropyridazine fragment ion 1

^b^SCP 2: Sulfachloropyridazine fragment ion 2

^c^SMZ-^13^C_6_ (SI): Sulfamethazinephenyl-^13^C_6_ hemihydrate (Internal Standard)

^d^Q1: Quadrupole 1

^e^Da: Dalton

^f^Q3: Quadrupole 3

^g^DP: Declustering potential

^h^V: Volt

^i^EP: Entrance potential

^j^CE: Collision Energy

^k^CXP: Collision Cell Exit Potential

^l^RT: Retention Time

### In-house validation of the analytical methods

An internal protocol for validation of the analytical method was designed following the recommendations from EU Commission Decision 2002/657/EC [45]. The protocol included the assessment of different parameters to ensure the methodology was suitable for detecting sulfachloropyridazine in feathers and edible tissues of broiler chickens. Namely, it assessed selectivity, specificity, linearity of the calibration curve, recovery and precision.

The Limit of Detection (LOD) was established based on a signal-to-noise ratio greater than 3:1. Meanwhile, the Limit of Quantification (LOQ) was determined by calculating the sum of the LOD plus 1.64 times the standard deviation (based on the results from 20 samples of each matrix, fortified at the LOD). Recovery was calculated through blank samples fortified at 0.1, 0.4 and 1 times the MRL for muscle tissue, as established by EU (100 μg kg^-1^). Precision was determined through the analysis of repeatability and intralaboratory reproducibility.

### Depletion study

Persistence of sulfachloropyridazine in feathers was calculated following the statistical method defined by the ‘Guideline on Approach Towards Harmonization of Withdrawal Periods’ EMA/CVMP/SWP/735325/2012 of the European Medicines Agency [37]. This guideline describes the statistical approach that the agency recommends for establishing withdrawal periods, as well as the minimum number of sampling points and animals that are required to ensure statistical robustness. In the case of chickens though, the required number of animals per sampling point was determined according to the guideline VICH GL48 on ‘Studies to evaluate the metabolism and residue kinetics of veterinary drugs in food-producing animals: marker residue depletion studies to establish product withdrawal periods’ (EMA/CVMP/VICH/463199/2009) [46]. This guideline specifies that a minimum of 6 birds are required per sampling point for poultry depletion studies.

For the regression analysis of the concentrations found in feather samples, these were plotted as a semi-logarithmic scale of concentration against time, for each sampling point. Then, a linear regression analysis was performed on the final elimination phase, considering a confidence level of 95%. Based on this plot and considering a slope with a 95% of confidence, we calculated the time (rounded to full days) when concentrations fell below the threshold level, which was characterized as any concentration lesser or equal than the LOD defined for the technique.

## Results

### In-house validation

**Fig 1:**
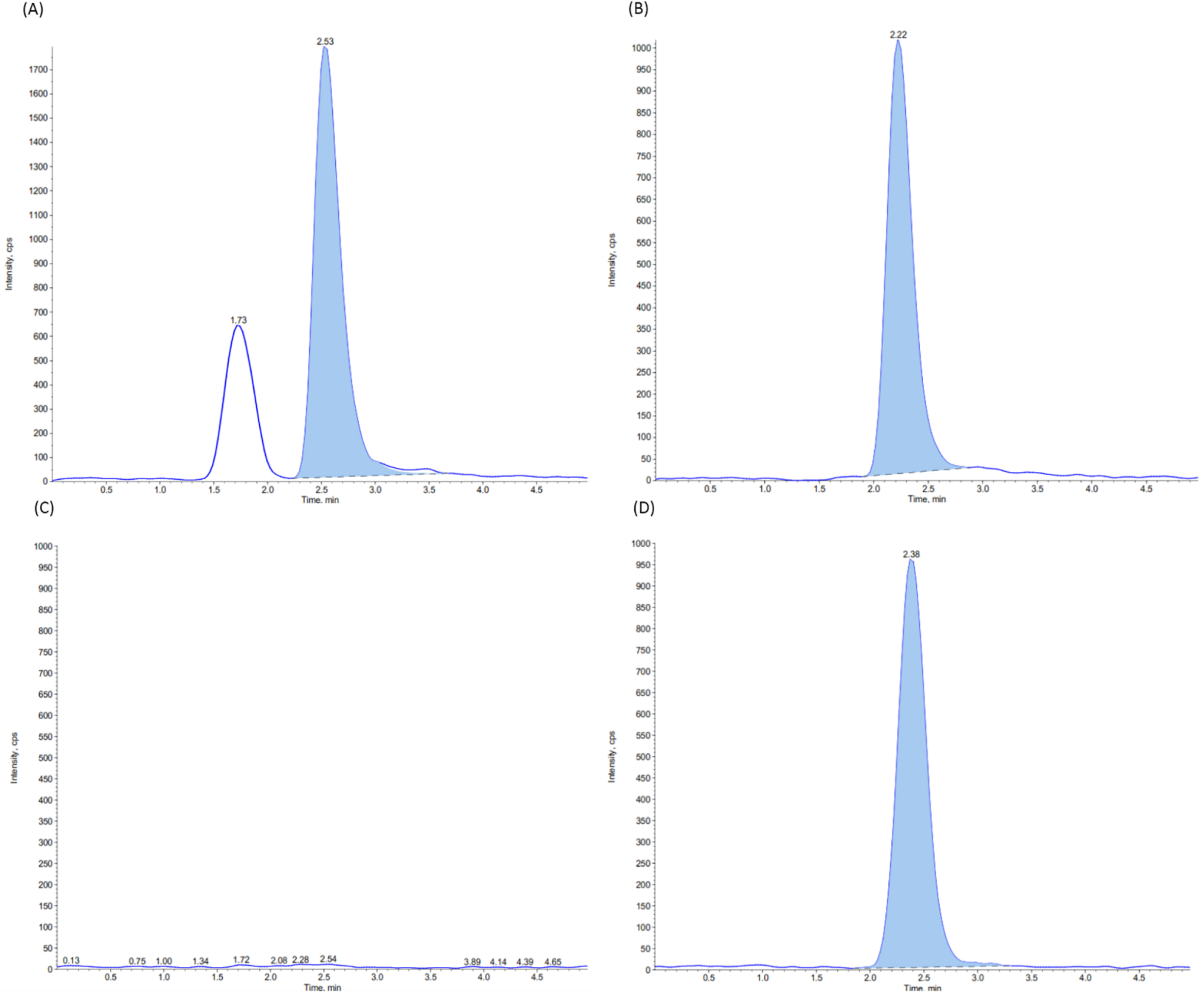
Chromatograms of sulfachloropyridazine in chicken feathers samples. (A) Pure standard SCP solution; (B) Pure Internal Standard solution (SMZ-^13^C_6_); (C) SCP in chicken feather samples, fortified at 10 μg Kg^-1^; (D) Internal Standard (SMZ-^13^C_6_) in chicken feather samples, fortified at 4.8 μg Kg^-1^; (E) Blank sample; (F) Internal Standard (SMZ-^13^C_6_) in chicken feather samples certified free of SCP residues.

Specificity of the analytical methods was determined using 20 blank samples. No interferences were detected for these samples on the analyte retention time for either feather, muscle or liver matrices (Fig 1 and S1 Fig).

An LOD of 10 μg kg^-1^ was established for this analytical methodology on feathers and liver, whereas on muscle samples it was set at 5 μg kg^-1^, with a signal-to-noise ratio greater than 3:1. Meanwhile, LOQ for sulfachloropyridazine were set at 14.6 μg kg^-1^, 6.2 μg kg^-1^, and 12.7 μg kg^-1^, for feathers, muscle, and liver samples, respectively.

Calibration curves were fortified on each matrix at concentrations of 10, 20, 40, 80 and 100 μg kg^-1^. These curves showed a determination coefficient (R^2^) greater than 0.98 for the feather matrix, and of 0.96 for both muscle and liver matrices (Table 2).

**Table 2:** In-house validation parameters.

Parameters	Feather	Muscle	Liver
**Linearity of calibration curve**	R^2^ CC1^a^: 0.9836 R^2^ CC2^b^: 0.9957 R^2^ CC3^c^: 0.9884	R^2^ CC1^a^: 0.9805 R^2^ CC2^b^: 0.9863 R^2^ CC3^c^: 0.9891	R^2^ CC1^a^: 0.9836 R^2^ CC2^b^: 0.9602 R^2^ CC3^c^: 0.9667
	CV^d^: 0.62%	CV: 0.44%	CV: 1.24%
**Repeatability**	10 ng/gr: 23.3%	10 ng/gr: 8.65%;	10 ng/gr: 20.8%
**Reproducibility**	10 ng/gr: 26.2%	10 ng/gr: 8.65%;	10 ng/gr: 24.9%

Results for linearity and precision parameters, by analytical matrix

^a^R^2^CC1: Coefficient of determination for Calibration Curve 1

^b^R^2^CC2: Coefficient of determination for Calibration Curve 2

^c^R^2^CC3: Coefficient of determination for Calibration Curve 3

^d^CV: Coefficient of variation

As for the recovery percentages, these fluctuated between 98 to 101 percent in all matrices, and for all fortification levels. Table 2 presents the average and coefficients of variation (CV) calculated for each fortification level (10, 40 and 100 μg kg^-1^) that were used to determine the precision, assessed through repeatability and intralaboratory reproducibility (S1 Dataset).

### Quantification of sulfachloropyridazine residues in samples

Sulfachloropyridazine concentrations in muscle, liver, and feather samples were quantified via a lineal regression analysis of the calibration curves on their respective fortified matrices. To this end, we considered those curves that presented an R^2^≥0.95.

To quantify feather samples at sampling points 1, 2, and 3, calibration curves were prepared with concentrations ranging from 100 to 1000 μg kg^-1^. However, sulfonamide concentrations detected by the equipment were too high for those samples that were collected on the first sampling. Therefore, those samples were extracted and analyzed again but using a new fortified calibration curve that included concentrations within the range actually found in the samples. This allowed us to avoid extrapolating quantifications. In the case of sampling points 4, 5, and 6, the calibration curves used for quantification included concentrations in the range of 10 to 100 μg kg^-1^. As for the residues in muscle and liver samples, these were quantified using calibration curves that include a concentration range of 10 to 100 μg kg^-1^.

The concentration that were quantified in muscle samples at day 7 after ceasing treatment were below the MRL, as it is currently established for this matrix in both Chile and the EU (100 μg kg^-1^). Meanwhile, concentrations in liver samples at the same timepoint were below the LOD for the analytical methodology. On the other hand, concentrations in feather samples on that same day reached an average of 2,858.78 μg kg^-1^ of sulfachloropyridazine (S2 Fig). Later on, at day 14 after ceasing treatment, analyte concentrations in the feather matrix declined approximately 84.65%, down to an average of 438.89 μg kg^-1^. However, in spite of such reduction, the concentrations that were detected and quantified at day 21 after ceasing treatment were of 183.39 μg kg^-1^, on average. Table 3 presents the average sulfachloropyridazine concentrations for different matrices, and for each time point after treatment.

**Table 3:** Sulfachloropyridazine concentrations in feather, muscle and liver samples.

Sample Point	Days after treatment	Age	Average SCP^a^ concentration (μg kg^-1^) in feather	Average SCP concentration (μg kg^-1^) in muscle	Average SCP concentration (μg kg^-1^) in liver
1	7	16	2,858.78	20.54	<LOD
2	14	23	438.89	<LOD	16.20
3	21	30	183.39	<LOD	ND
4	32	43	18.92	ND^c^	-
5	36	45	<LOD^b^	-	-
6	38	47	<LOD	-	-

Average concentration of sulfachloropyridazine detected in samples of feather, muscle and liver tissue from broiler chickens, after receiving an oral treatment of 10% sodium sulfachloropyridazine.

^a^SCP: sulfachloropyridazine

^b^LOD: Limit of Detection (10 μg kg^-1^)

^c^ND: Not Detected

### Depletion of sulfachloropyridazine in feather samples

**Fig 2:**
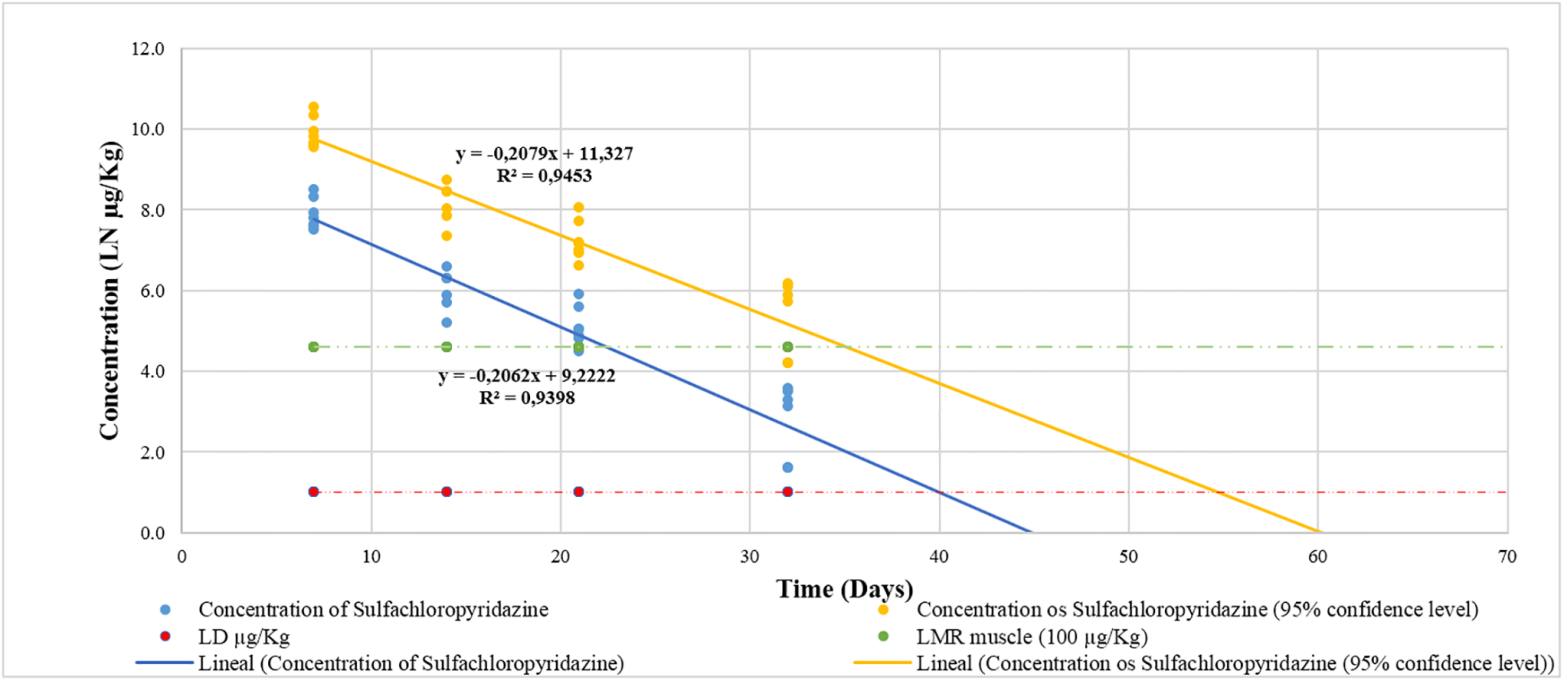
Depletion time of sulfachloropyridazine concentrations in chicken feather samples. The depletion time was calculated on day 55 (95% confidence level). The LOD of the analytical methodology (10 μg Kg^-1^) was considered as the cut-off point.

To determine the depletion time for feathers, sulfacloropyridazine concentrations were plotted against time on a semi-logarithmic scale, and a linear regression analysis was performed considering a 95% confidence level. Based on this curve, we determined that on day 55 (rounded up to a full day from 54.705) the concentrations of sulfachloropyridazine were less or equal than the LOD (10 μg kg^-1^) that had been established for this analytical technique (Fig 2 and S1 Table).

## Discussion

Chicken feathers are by-products from the poultry industry that are mainly used as an ingredient in diets fed to other animal species. Several studies have shown that drugs from different antimicrobial families persist for a longer period in feathers. Hence, the potential for transferring residues through the food-chain must be addressed [20–26].

To contribute in addressing these concerns, our research group developed an in-house validation protocol to ensure that the analytical method is fit for its purpose of detecting and quantifying sulfachloropyridazine in feather, muscle and liver samples, precisely and confidently.

Our results show that sulfachloropyridazine an antimicrobial drug from the sulfonamides class persists in feathers for a longer period and at greater concentrations than in edible tissues. In particular, we calculated a depletion time of 55 days for feather samples, after ceasing treatment (95% confidence interval) for feather samples. Meanwhile, concentrations in edible tissues fell below the established MRL for muscle and liver samples (100 μg kg^-1^) by the day 7 after treatment. These results agree with those reported by other researchers for the depletion behavior of antimicrobial drugs from different classes in both edible tissues and feathers.

In the case of edible tissues, Łebkowska-Wieruszewska and Kowalski [16], as weel as Li and Bu [17], found that sulfachloropyridazine reached high concentrations up to day 4 after ceasing treatment, approximately; afterward, its concentrations quickly declined below the LOD. Furthermore, Łebkowska-Wieruszewska and Kowalski [18] had previously detected this drug in muscle and liver samples of turkeys, where its concentrations diminished rapidly seven days after ceasing treatment.

Meanwhile, several studies have found residues of quinolones, tetracyclines and amphenicols in the feather matrix. These residues persisted for longer periods and at greater concentrations than in edible tissues such as muscle and liver. For example, San Martin et al. [20] studied quinolones and found that the concentrations of enrofloxacin and ciprofloxacin (a metabolite of enrofloxacin) in feathers were higher than those detected in edible tissues (muscle, liver and kidney). Similarly, Cornejo et al. [21] found that the concentrations of flumequine in feathers were higher than those quantified in liver and muscle samples. Later on, Cornejo et al. [22] also showed that both enrofloxacin and ciprofloxacin were transferred to feathers, and that these antimicrobials remained in them for a longer period. Similarly, the concentrations were higher than those found in edible tissues. In the same line, results from Jansen et al. [24] also indicate that depletion of enrofloxacin in feathers was slower than what is observed in matrices commonly used to supervise MRLs.

In the case of oxytetracycline, Berendsen et al. [23] studied its accumulation in feathers after easing treatment by analyzing individual feather segments. They found that part of the oxytetracycline residues built up into the feather rachis. Therefore, analyzing feathers for antimicrobial residues is actually a valuable tool. Recently, Cornejo et al. [26] reported that residues of oxytetracycline and 4-epi-oxytetracycline (its metabolite) persist for a longer period, and their concentrations are higher in feathers than what is observed in edible tissues of birds treated with this drug, even after meeting withdrawal periods.

In another study by Cornejo et al. [25], they determined that residues of florfenicol and florfenicol amine also remained in feathers for longer periods than the withdrawal time that has been established for muscle tissue.

Currently no other studies have been reported comparing the depletion of sulfonamides in feathers against its depletion in edible tissues. Our results agree with the general trend described by the aforementioned works, as on day 7 after treatment we found residue concentrations of 2,858.78 μg kg^-1^ in feathers, whereas in edible tissues these were of 20.54 μg kg^-1^. Also, the concentrations found were below the LOD for muscle and liver samples, respectively. Afterward, concentrations declined and reached 183.39 μg kg^-1^ on day 21 after treatment.

In a broad study, Jansen et al. [28] analyzed 20 chicken feather samples and detected active compounds in 23 out of 26 recorded treatments. Among these compounds there were some antimicrobials from the sulfonamides class, such as sulfachloropyridazine, which was present at a concentration of 1858 ng g^-1^.

Finally, the information currently available supports the necessity of monitoring and inspecting animal feed containing feather-derivative ingredients to avoid re-entry of drug residues into the food-chain. In this regard, this work shows that after treating broiler chickens with a commercial formulation of 10% sodium sulfachloropyridazine, feathers should not be used as an ingredient in animal diets; unless, of course, these are properly monitored until day 55 after ceasing treatment.

## Conclusions

The results in this work show that sulfachloropyridazine bioacumulate at high concentration levels in feathers, even though in muscle and liver samples concentrations fell below the LOD that was established for the analytical methodology. In addition, these concentrations persisted for a longer period in feathers. Therefore, this inedible by-product is an important re-entry route for antimicrobial drugs into the food chain.

The present work is the first to study sulfachloropyridazine depletion in feathers matrix and we anticipate that future studies could explore the impact of feather meal manufacturing processes on the behavior of this antimicrobial drug. Furthermore, feathers could be used by the poultry industry as a non-invasive sampling matrix suitable for monitoring different veterinary medications. The depletion behavior we have detailed in this work leads us to believe that this matrix could become an effective tool for the inspection of diets destined to feed different animal species and, therefore, aid on the design and application of new policies of antimicrobial surveillance.

## Supporting information

S1 Fig Chromatograms of sulfachloropyridazine in feather samples. Fortified samples at 10, 20, 40, 80 and 100 μg Kg^-1^, as well as the positive and negative controls (blank sample to sulfacholopyridazine). Sulfamethazinephenyl-^13^C_6_ hemihydrate (SMZ-13C6) as internal Standard at a concentration of 4.8 μg Kg^-1^.
(PDF)

S2 Fig Chromatograms of sulfachloropyridazine (SCP) residues in feather. Samples from experimental animals treated with sulfachloropyridazine. Sulfamethazinephenyl-^13^C_6_ hemihydrate (SMZ-13C6) was used as Internal Standard for the fortification of the samples.
(PDF)

S1 Dataset In-house validation of analytical methodology in feathers. Results for retention time, specificity, limit of detection, limit of quantification, linearity, recovery and precision parameters are detailed in this dataset.
(PDF)

S1 Table Concentrations of sulfachloropyridazine residues in feathers. Data of concentrations of sulfachloropyridazine residues in feathers samples, for each sampling point during depletion determination.
(PDF)
