# Supplementary figures and images for: Determination of sulfachloropyridazine residue levels in feathers from broiler chickens after oral administration using liquid chromatography coupled to tandem mass spectrometry
Source: PLoS One. 2018 Jul 5;13(7):e0200206. doi: 10.1371/journal.pone.0200206 (PMC6033452; doi:10.1371/journal.pone.0200206)

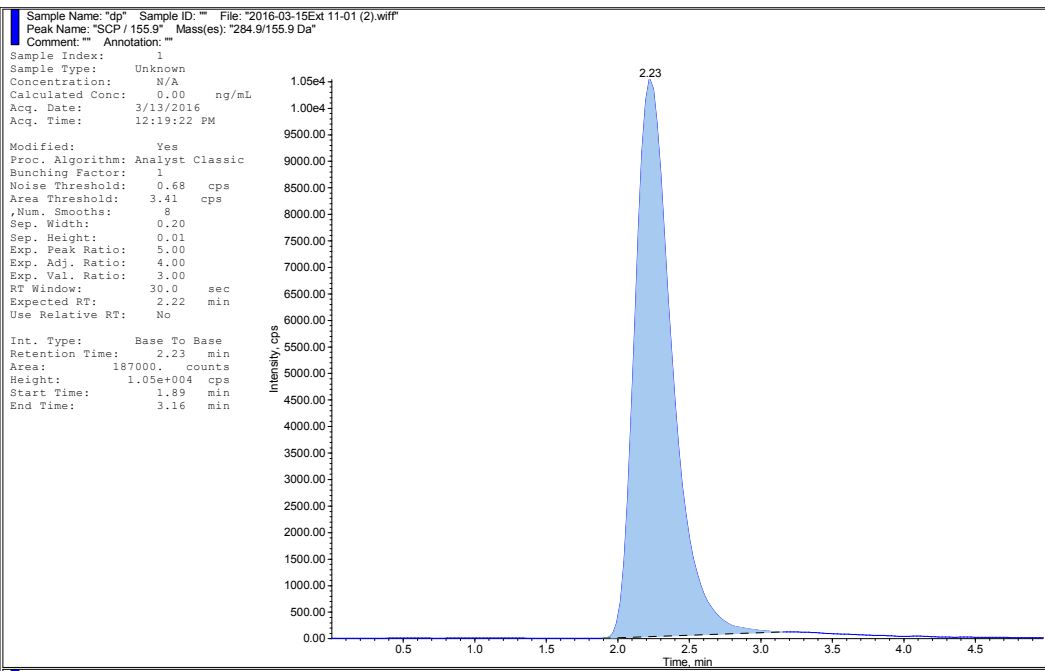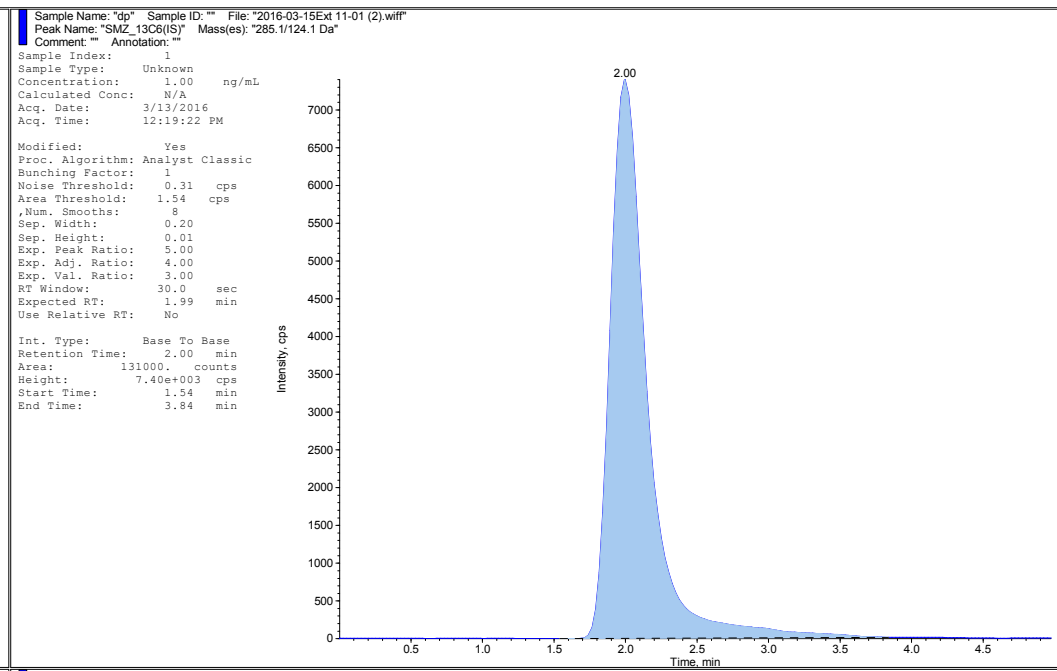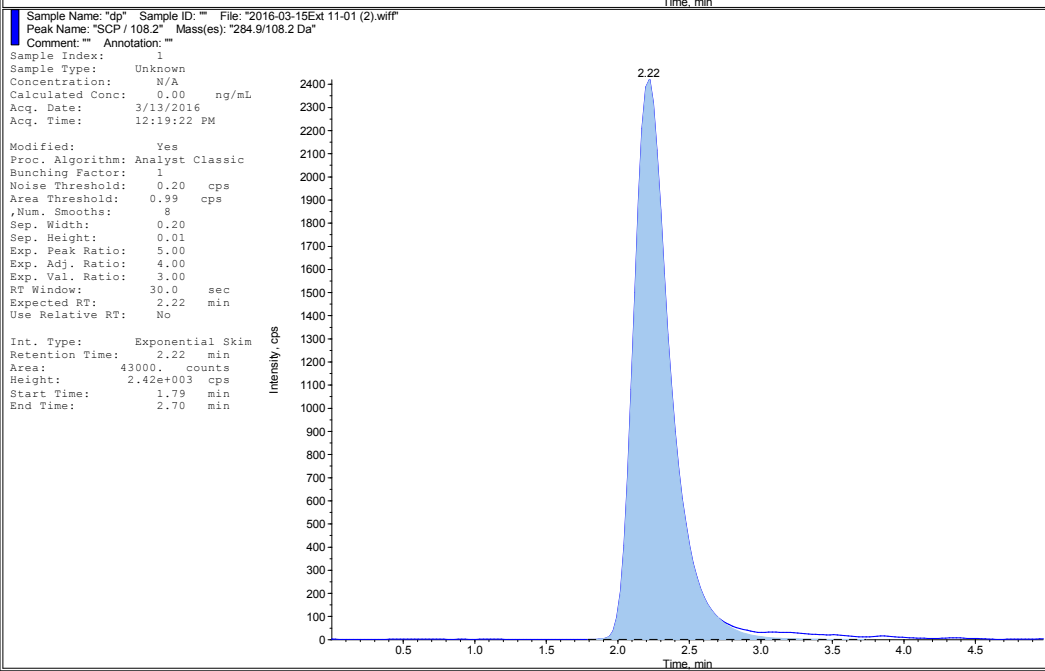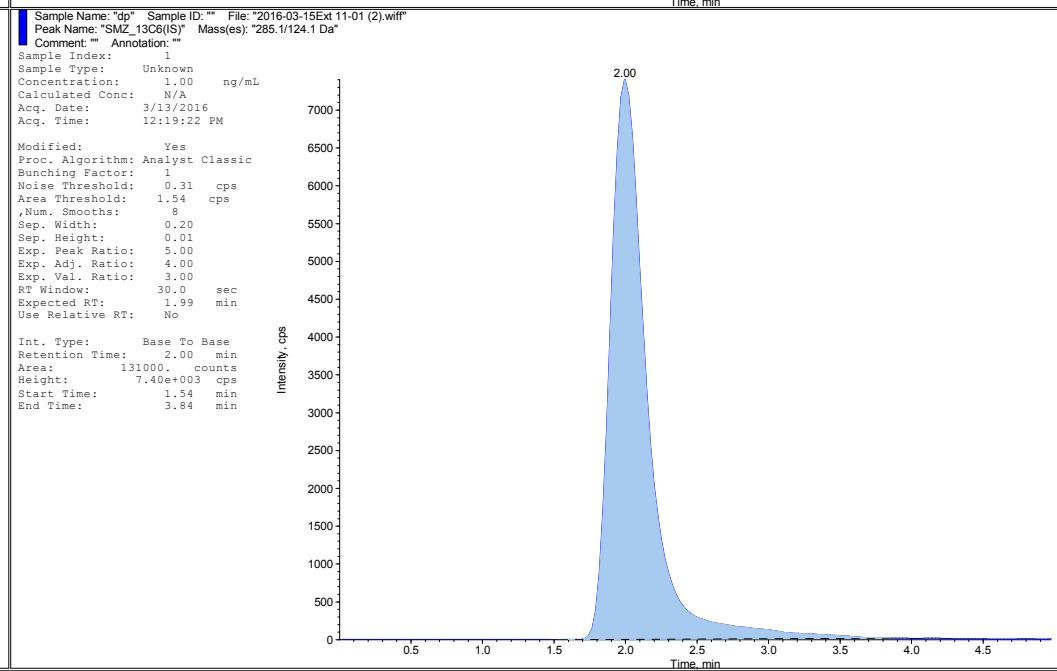

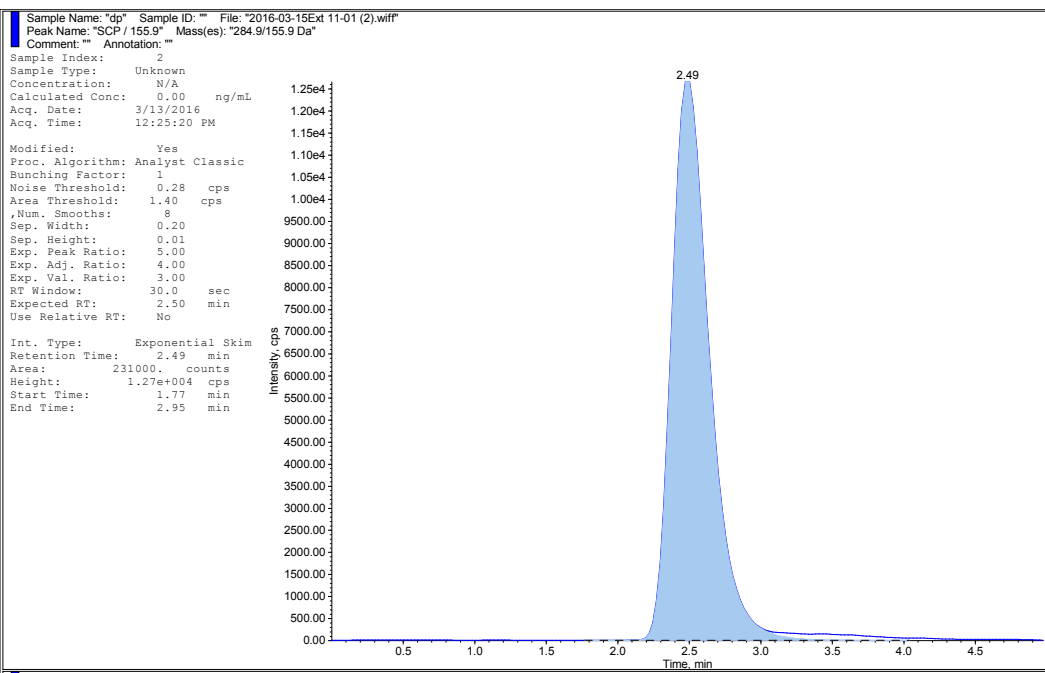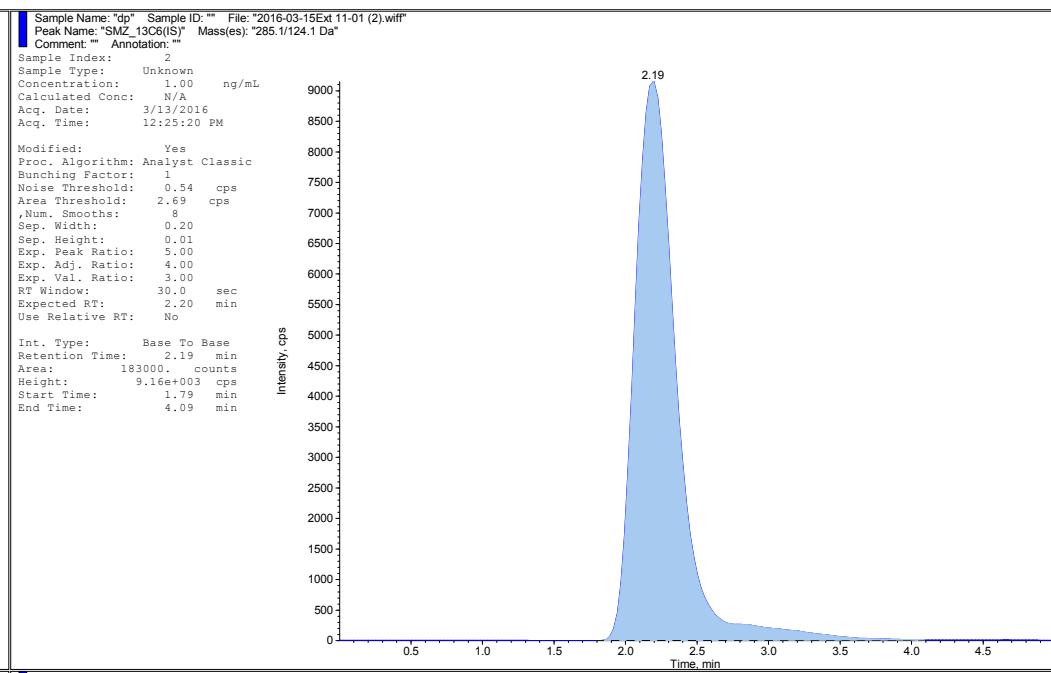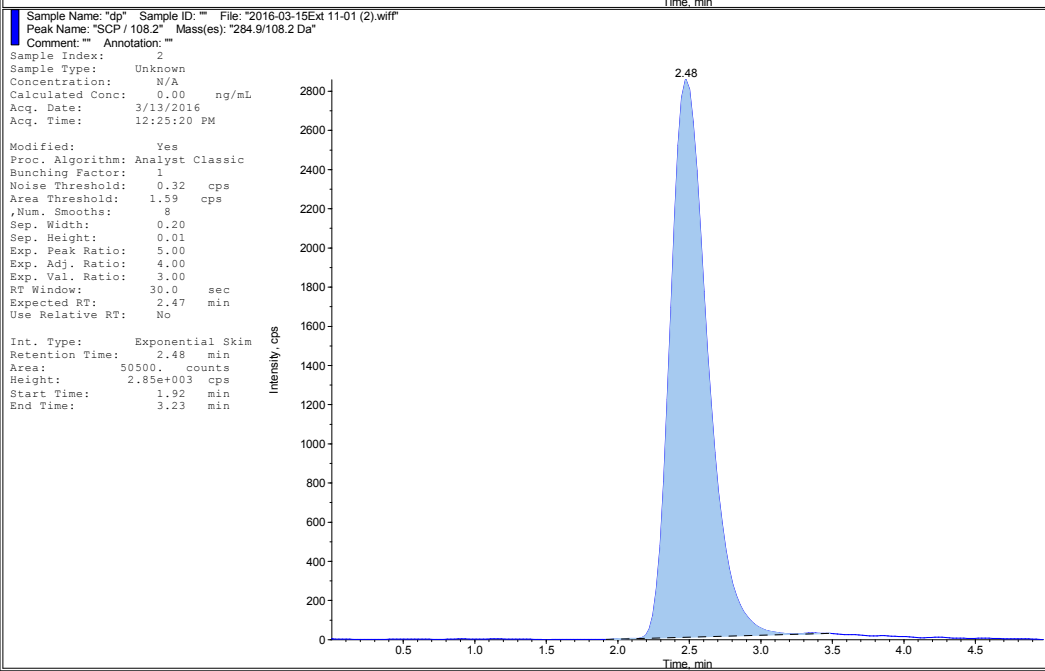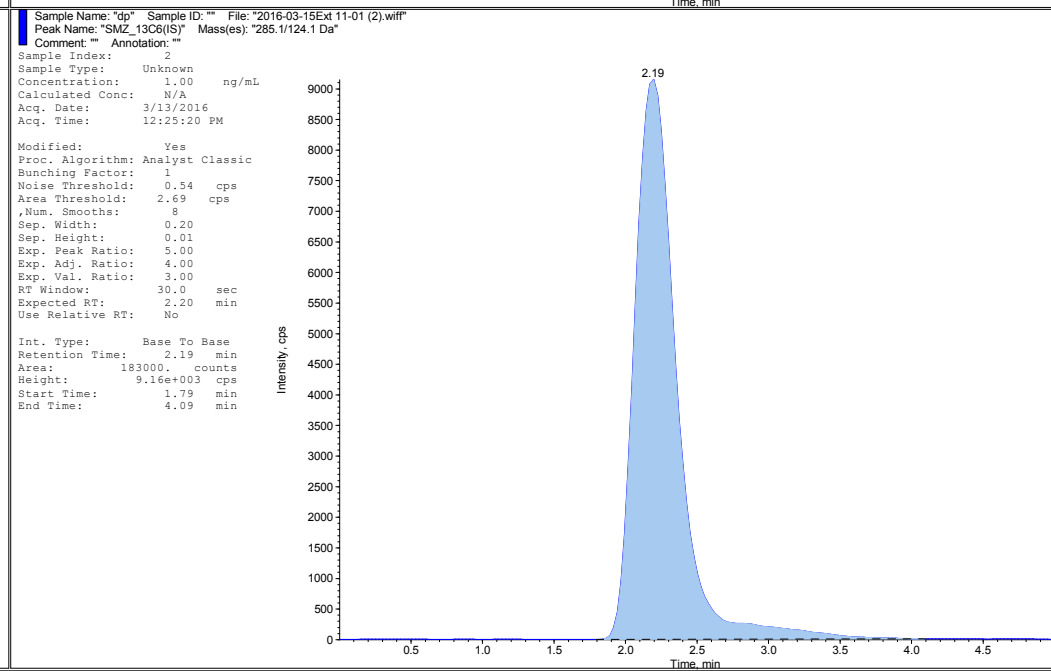

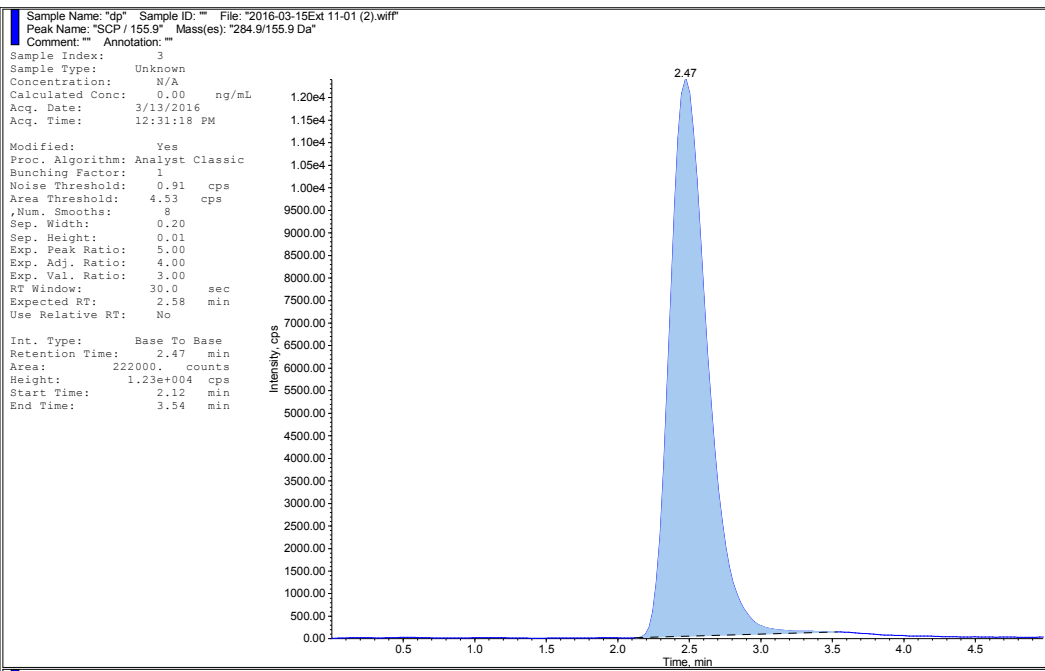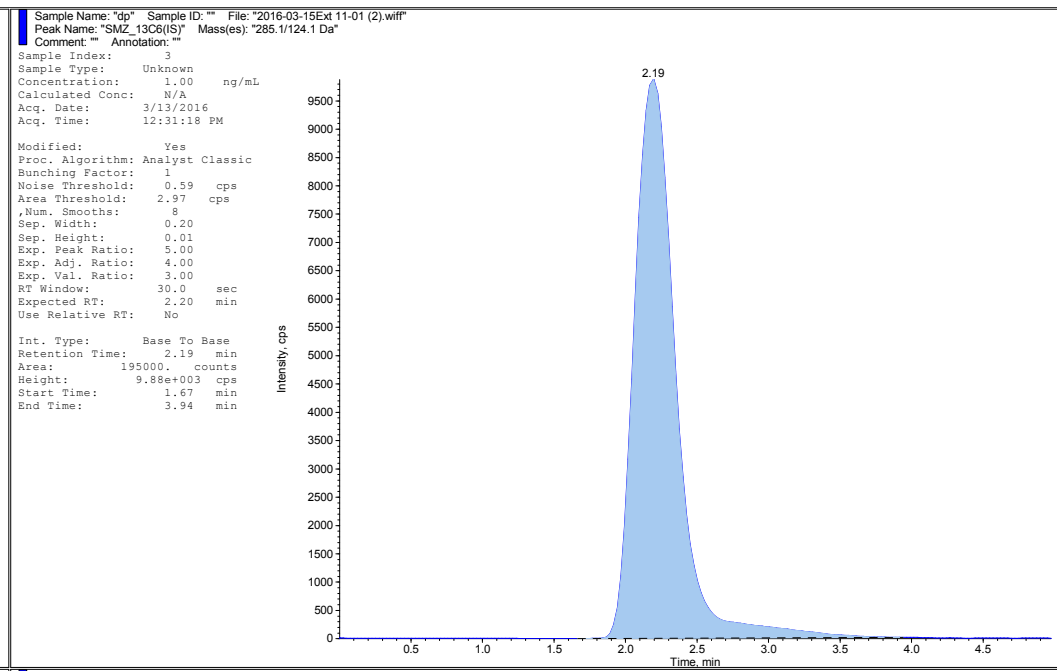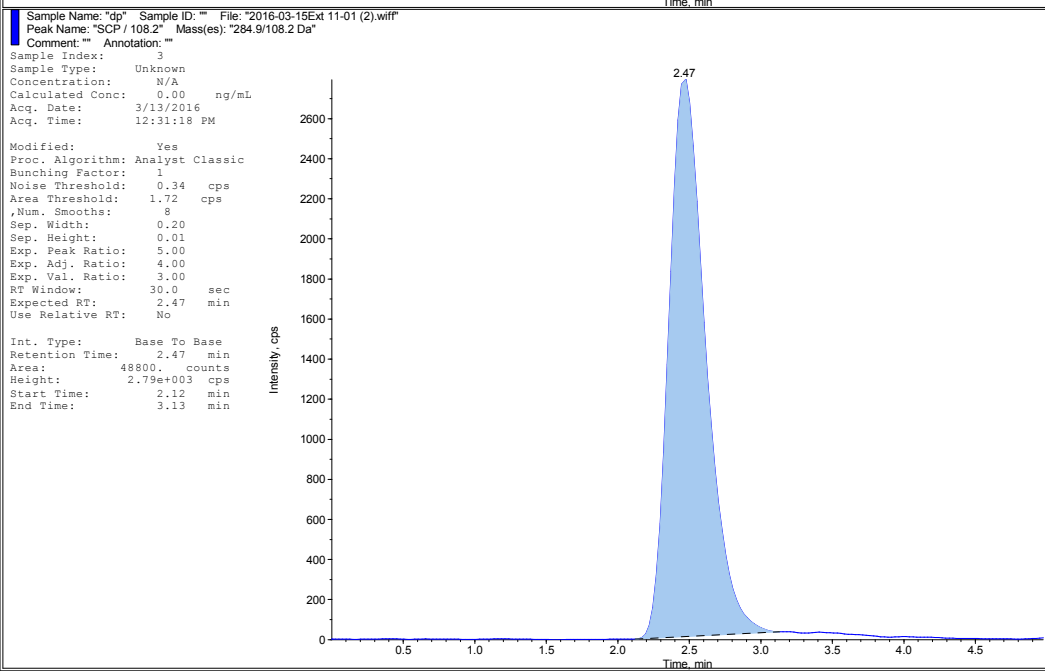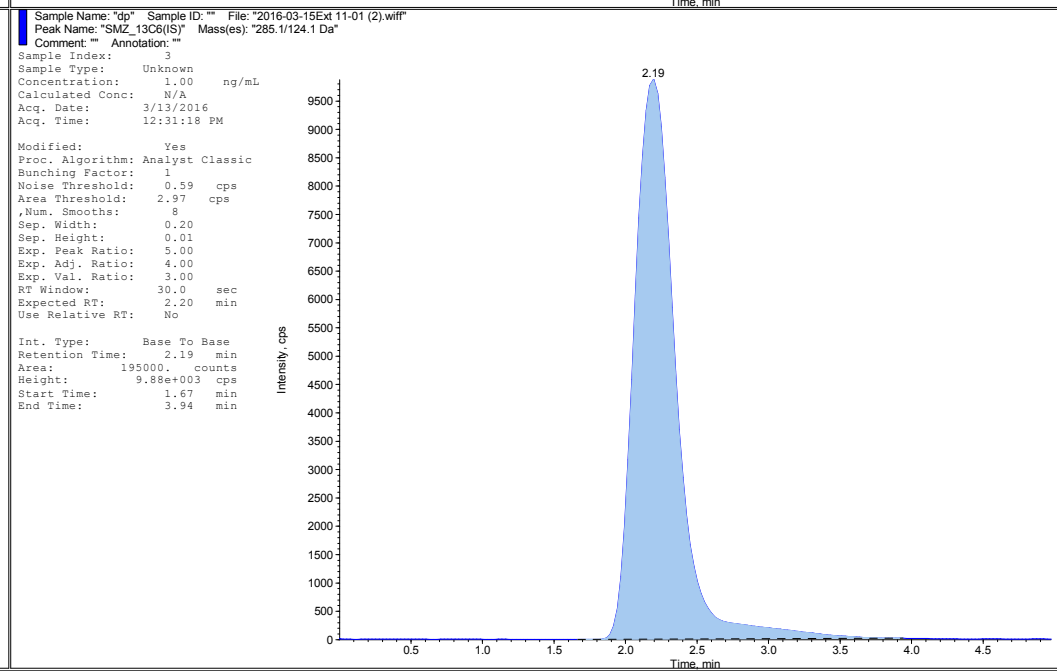

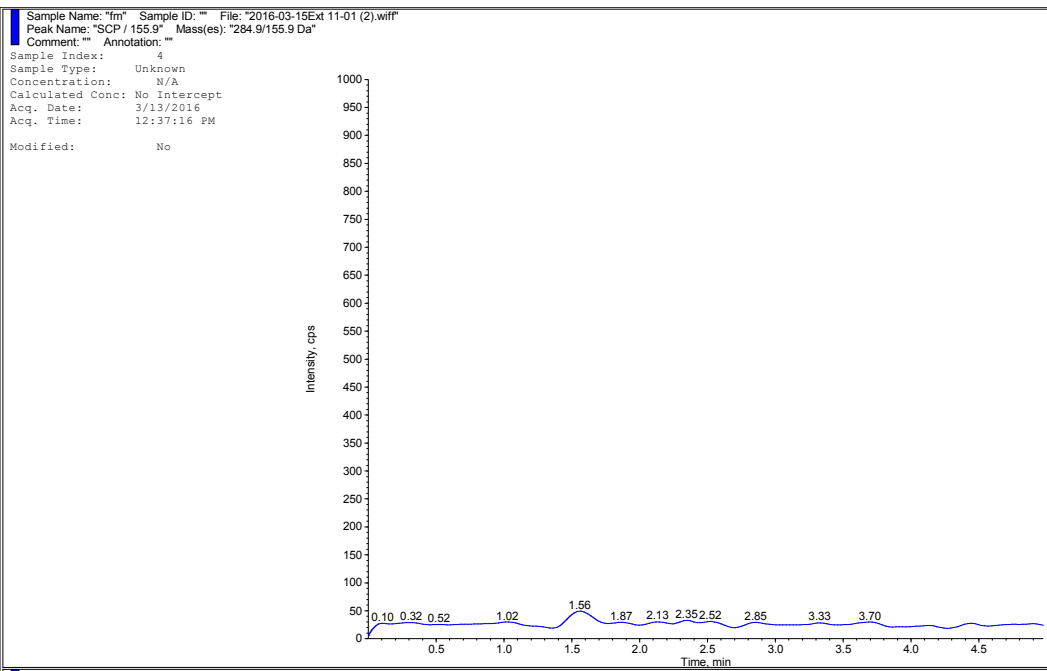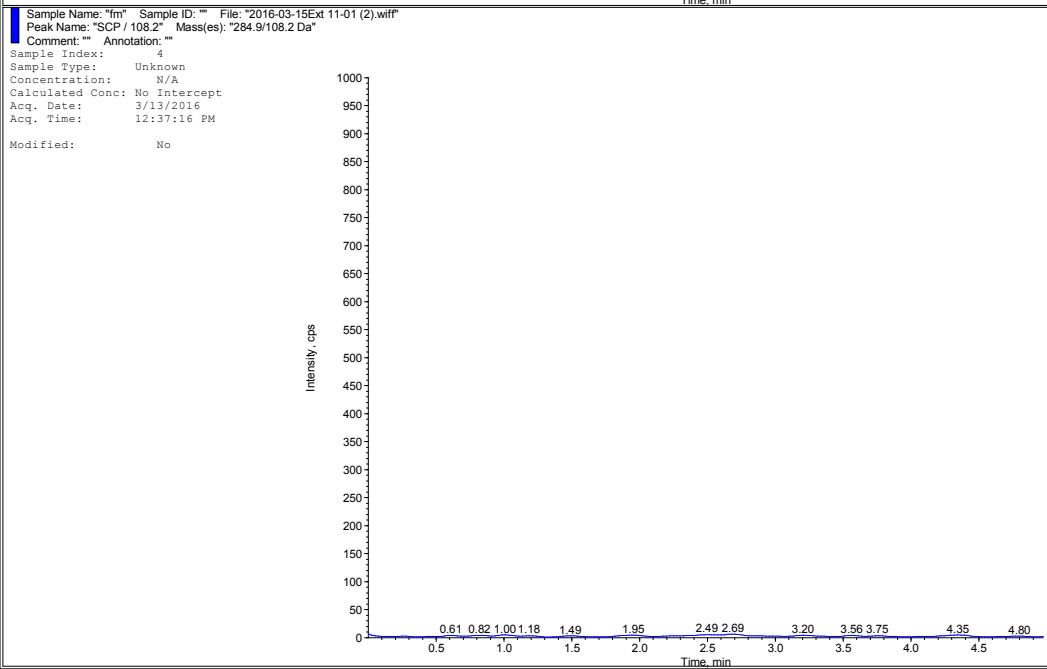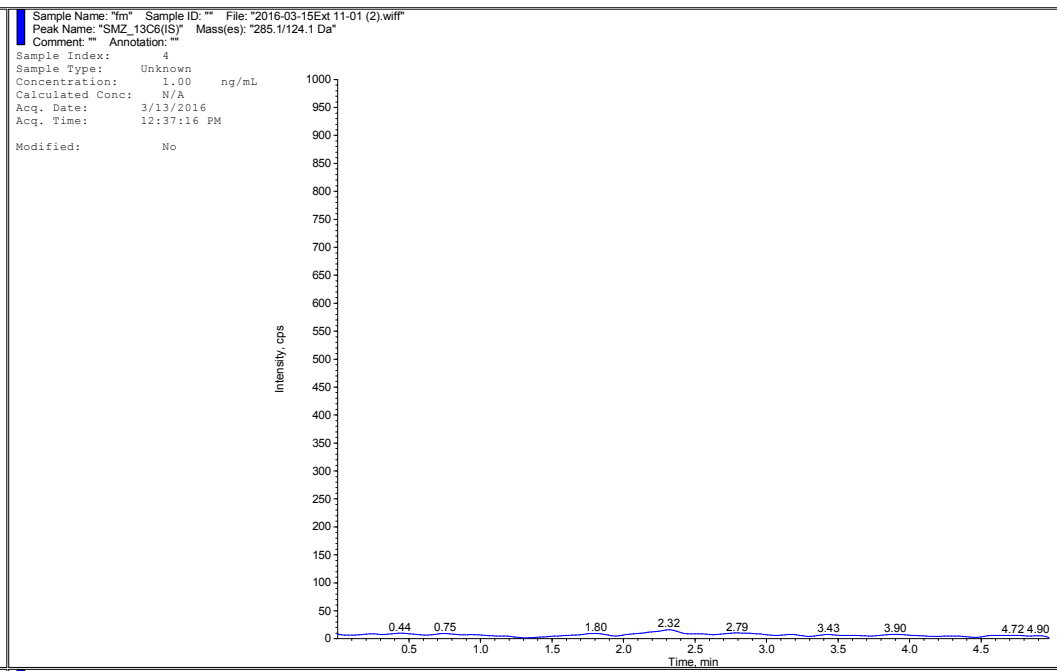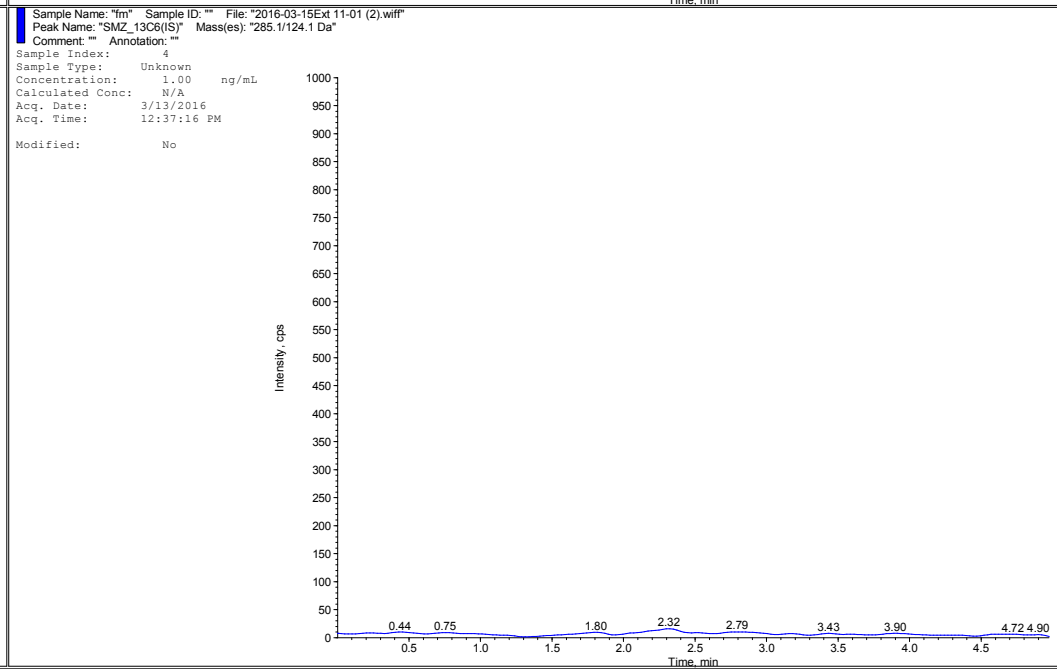

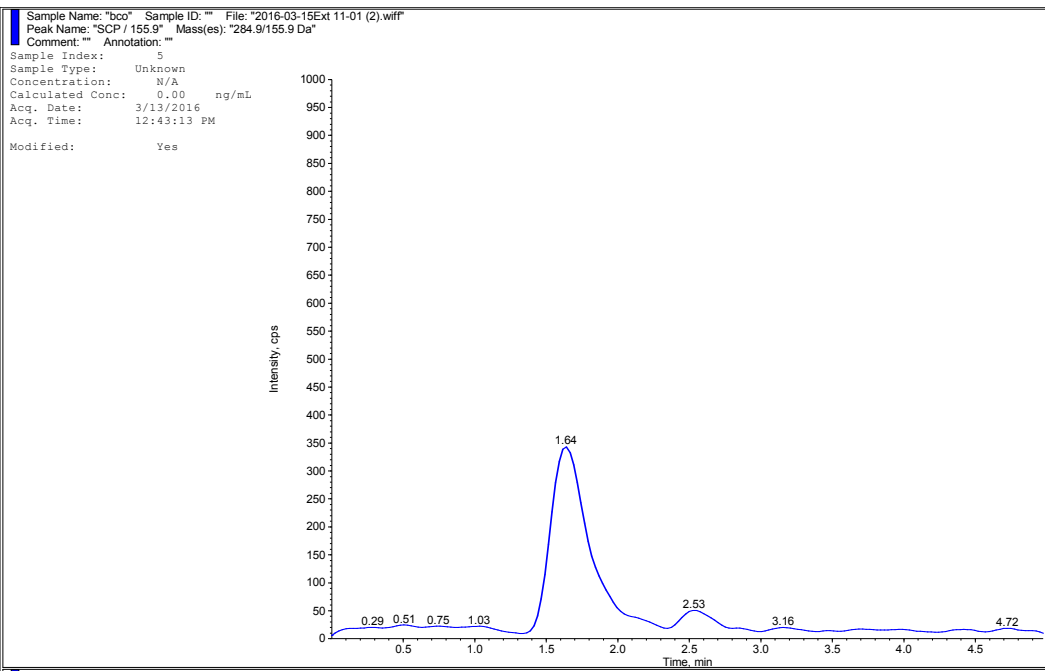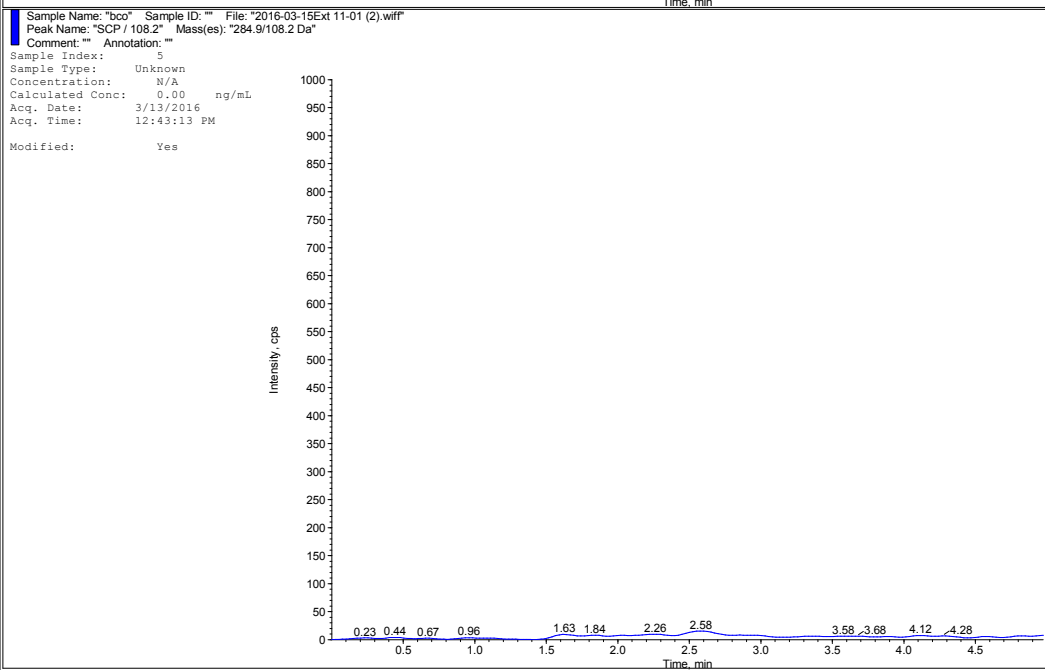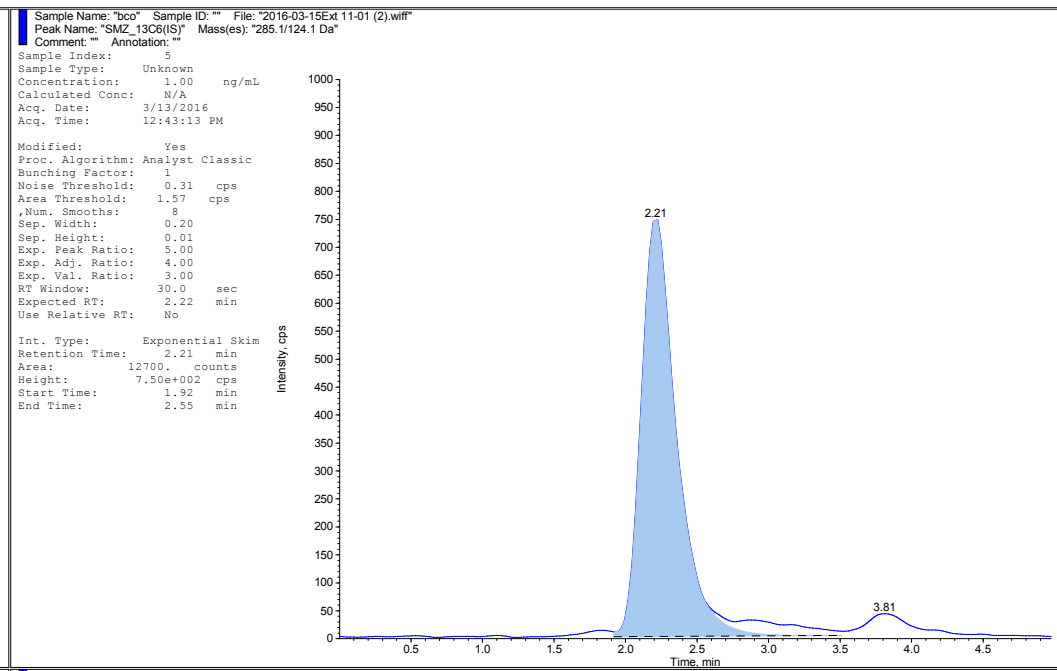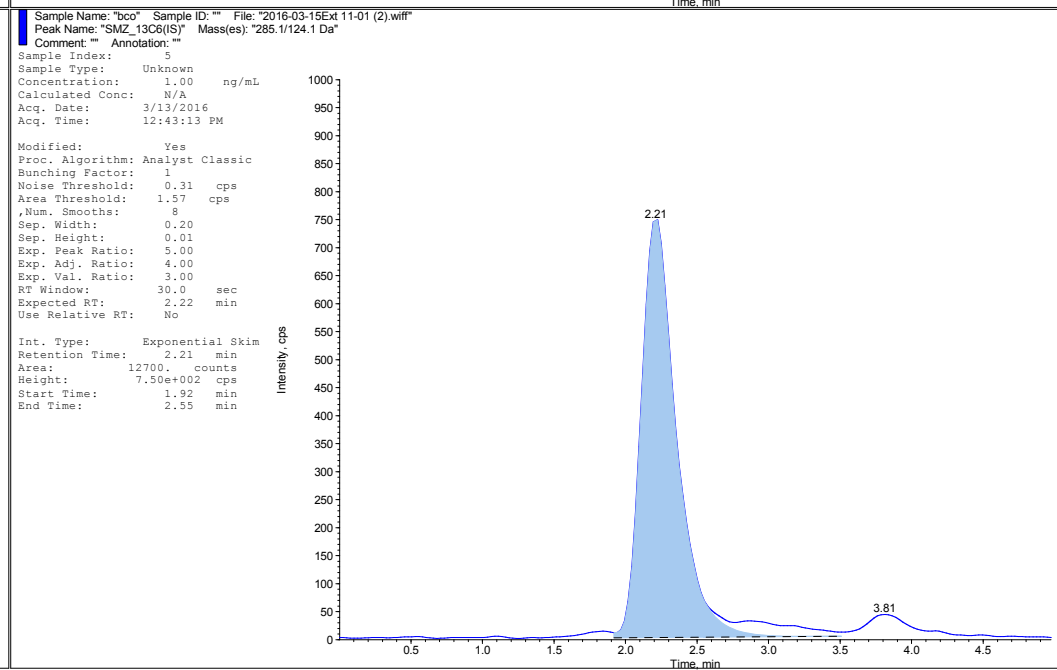

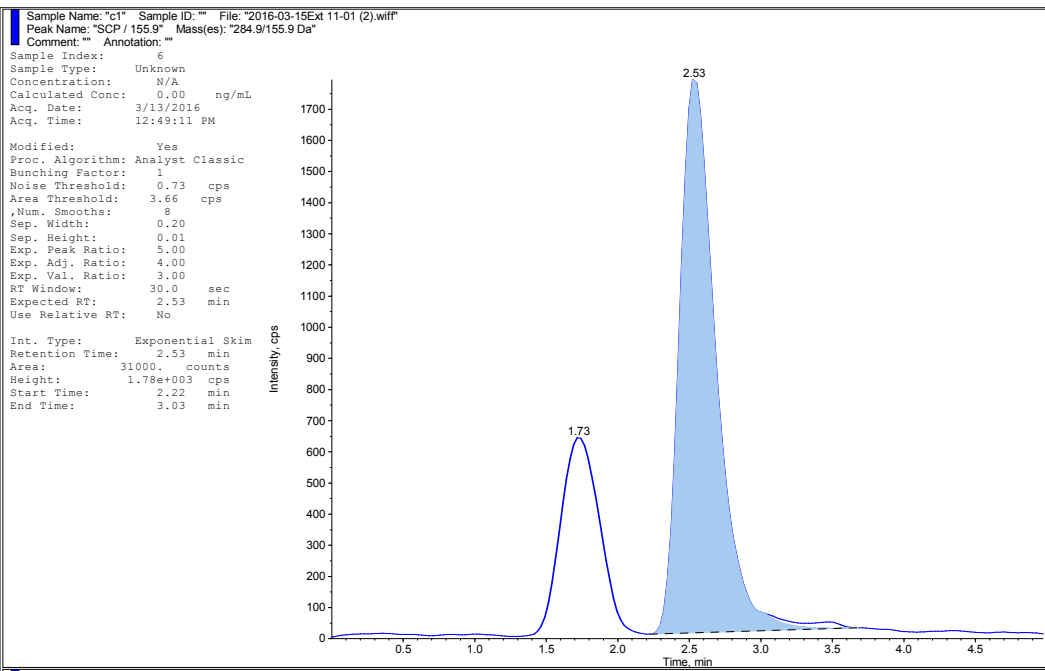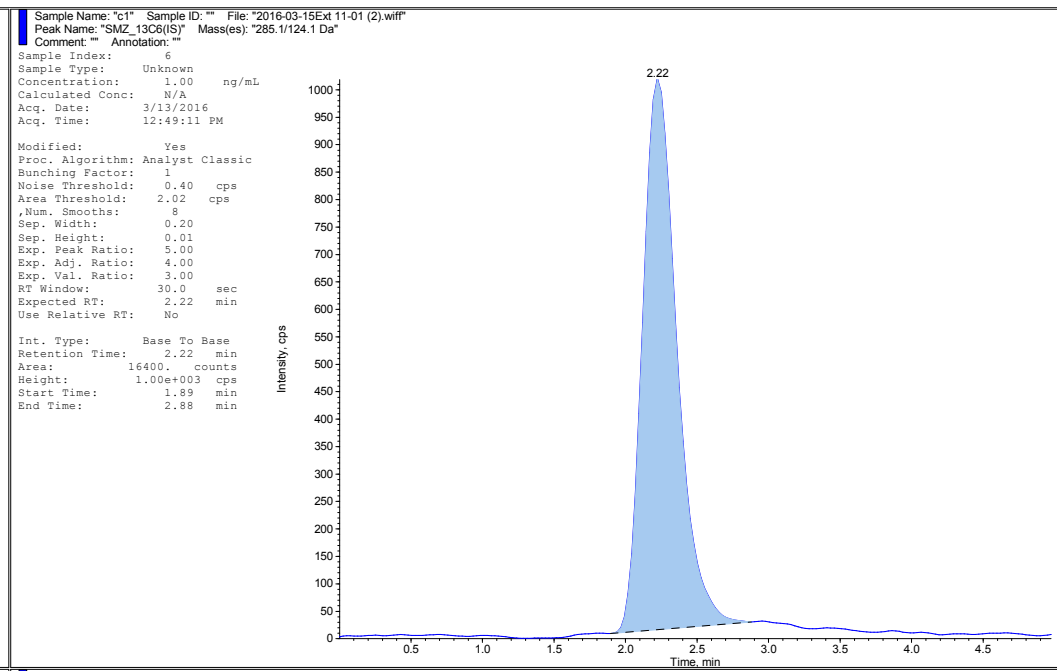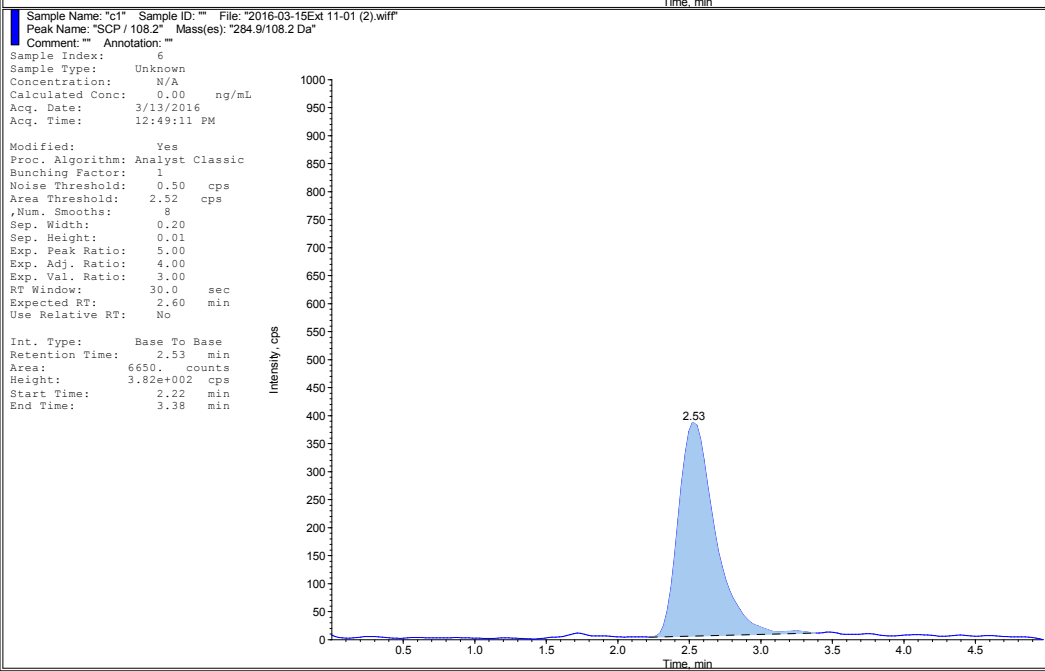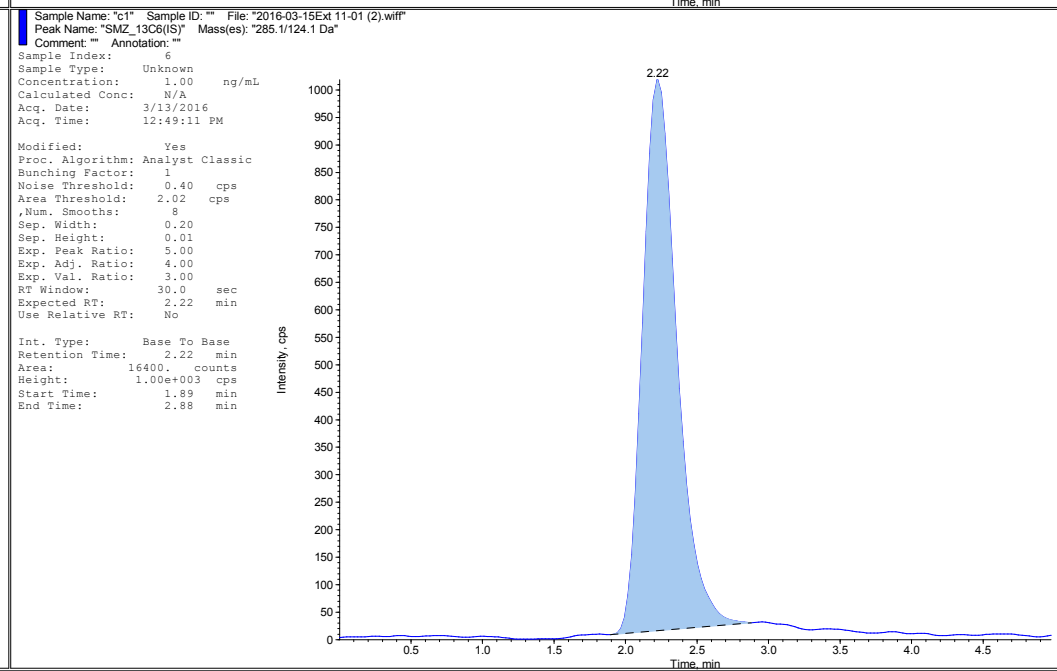

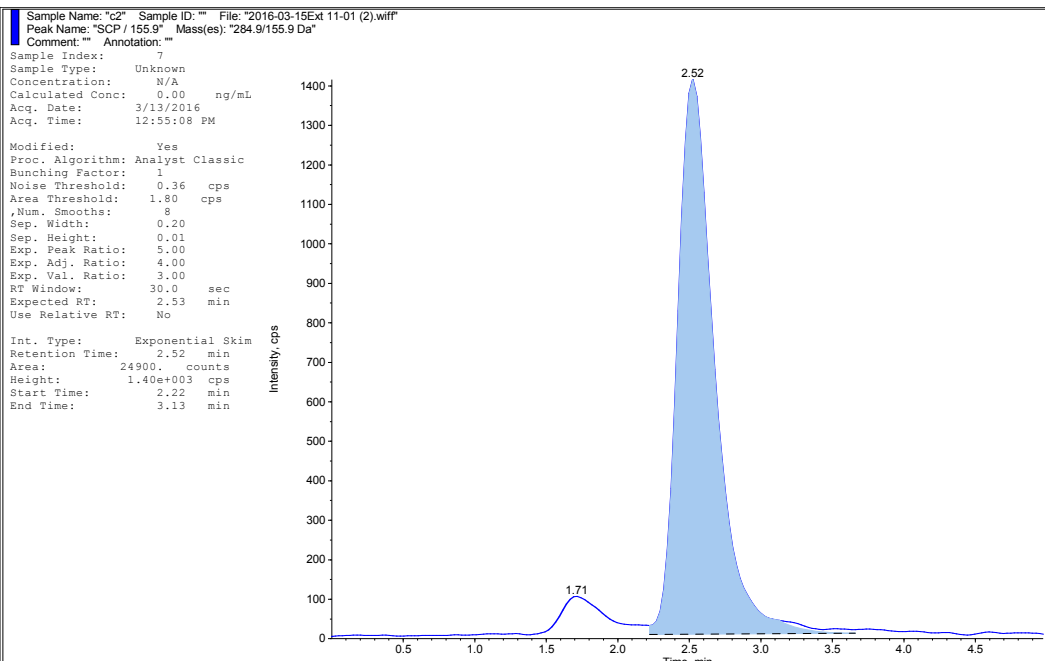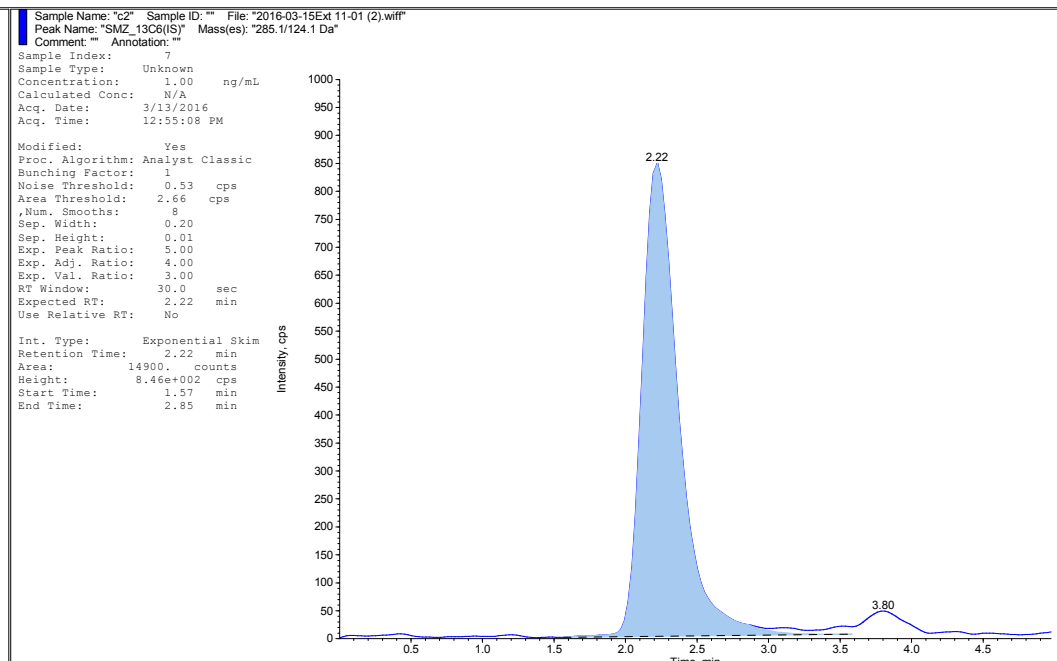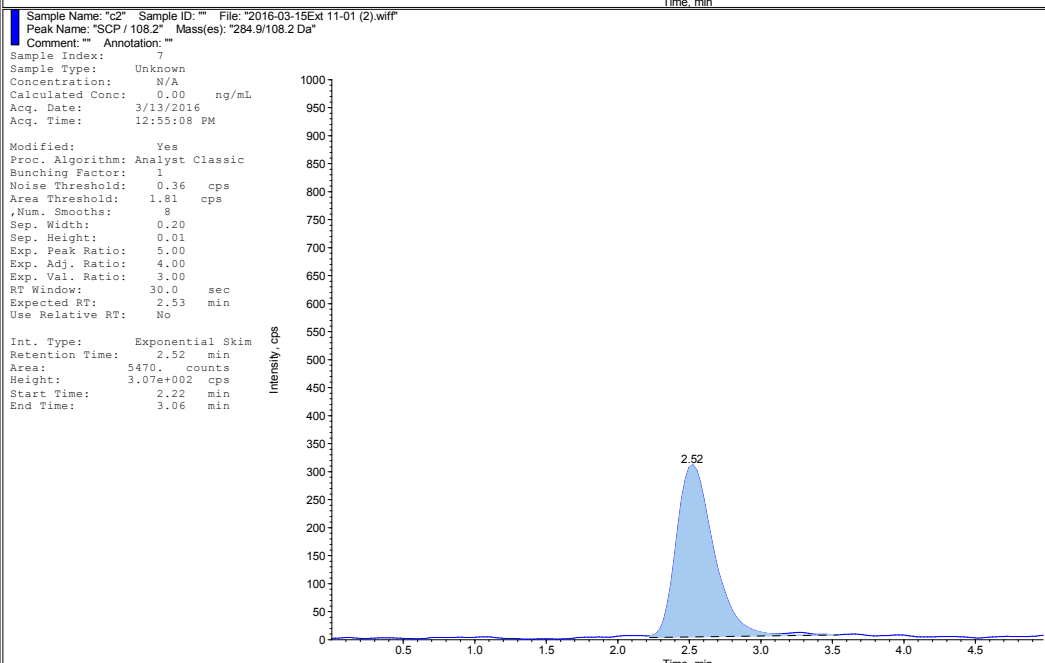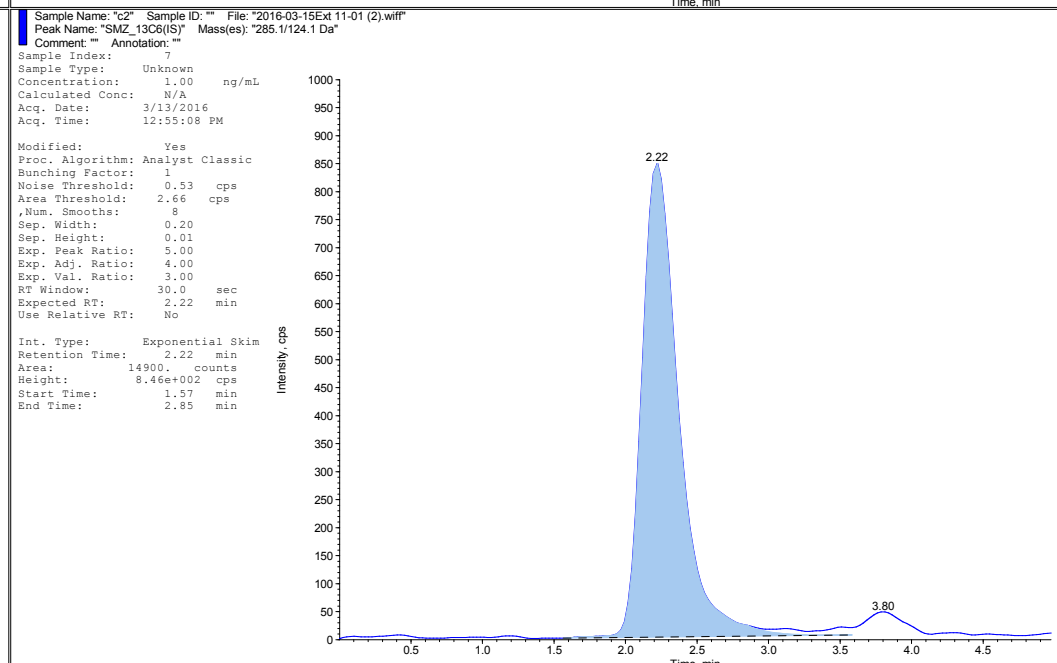

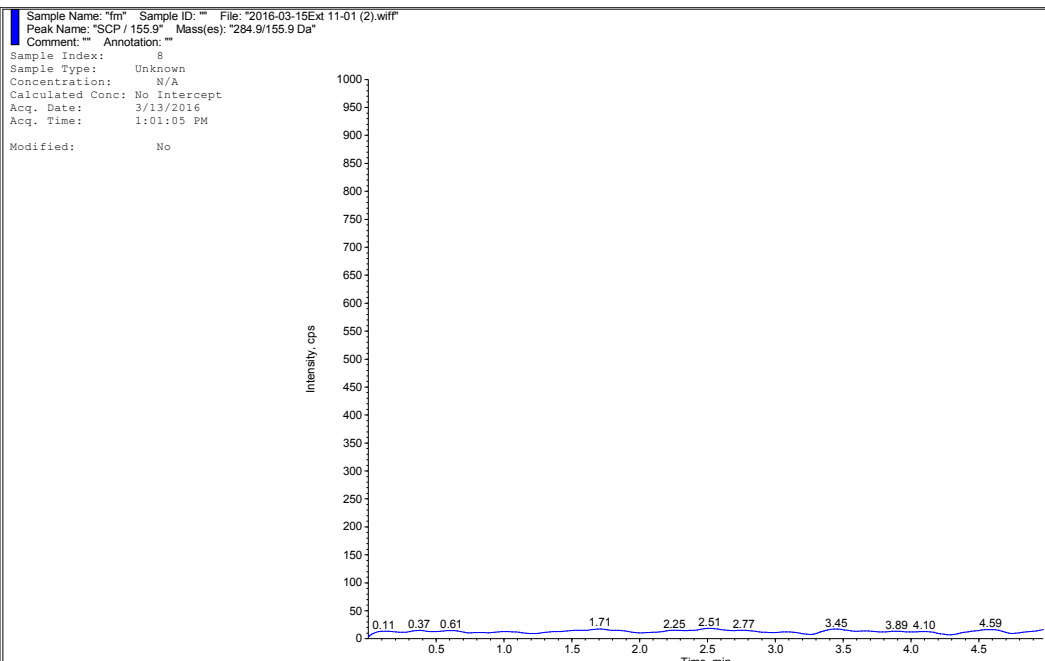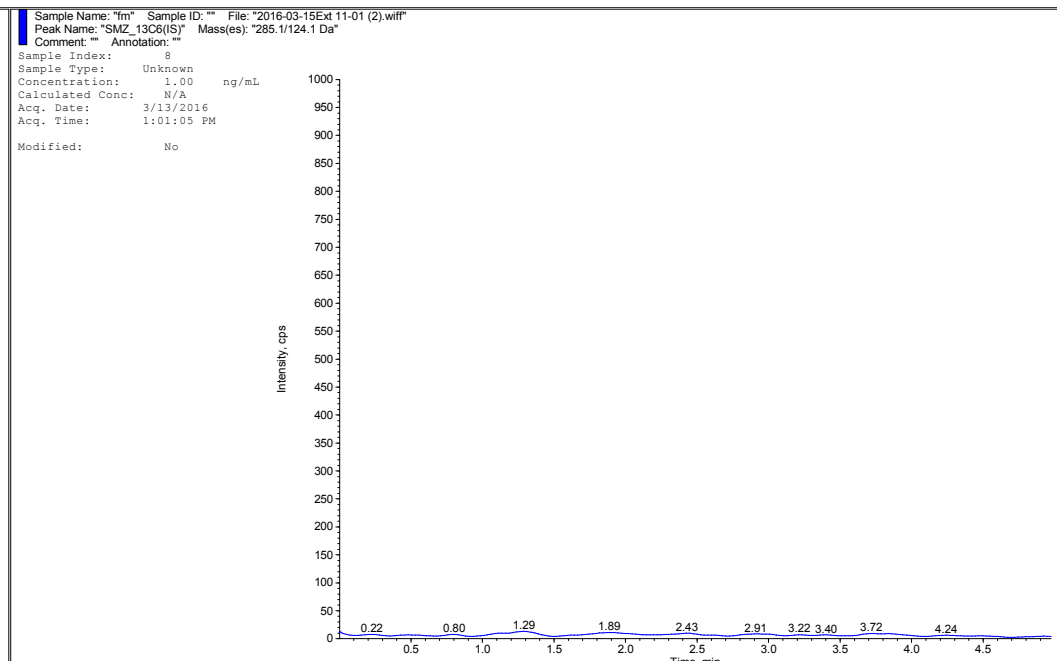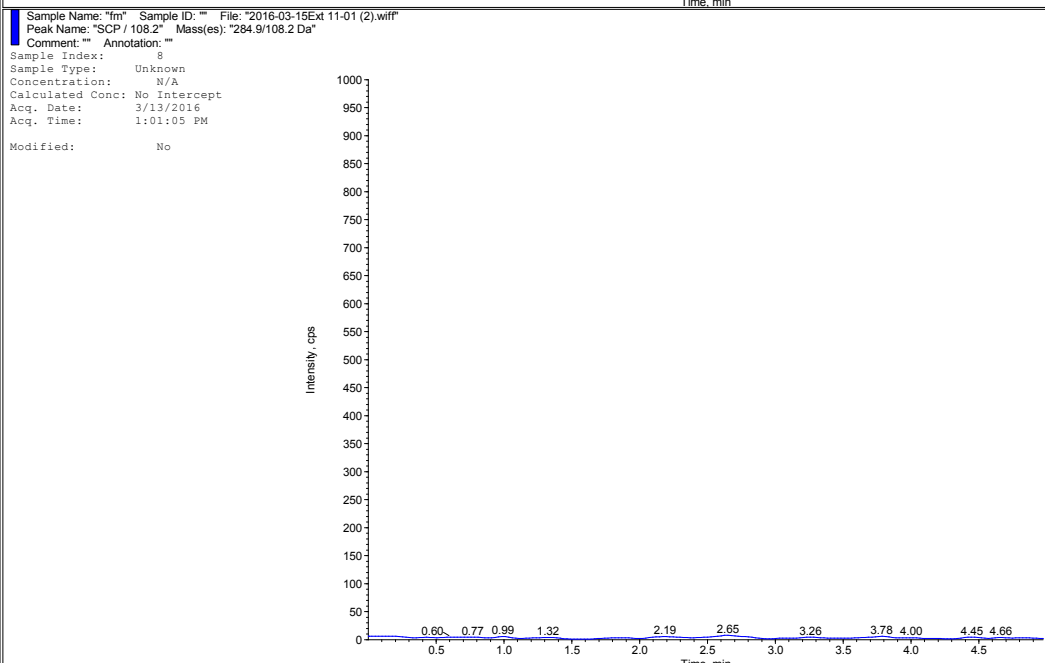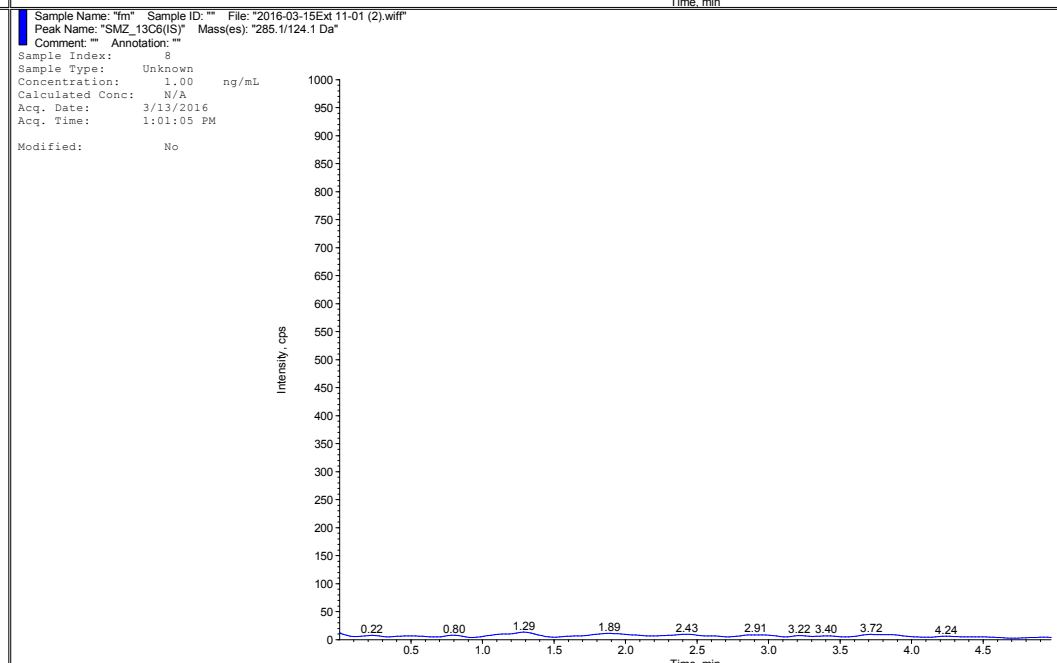

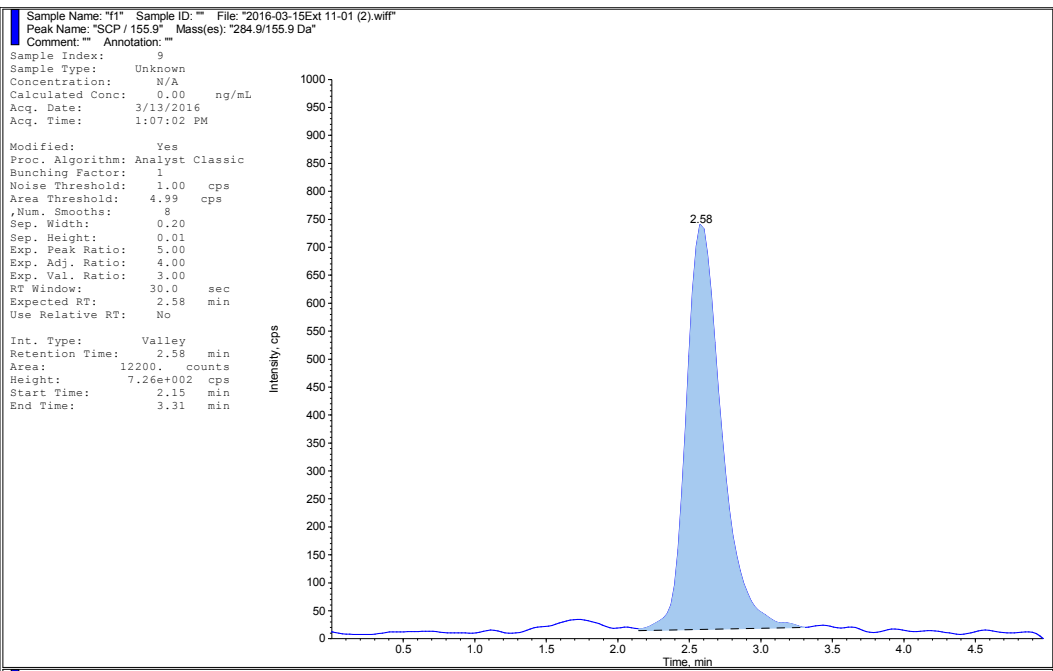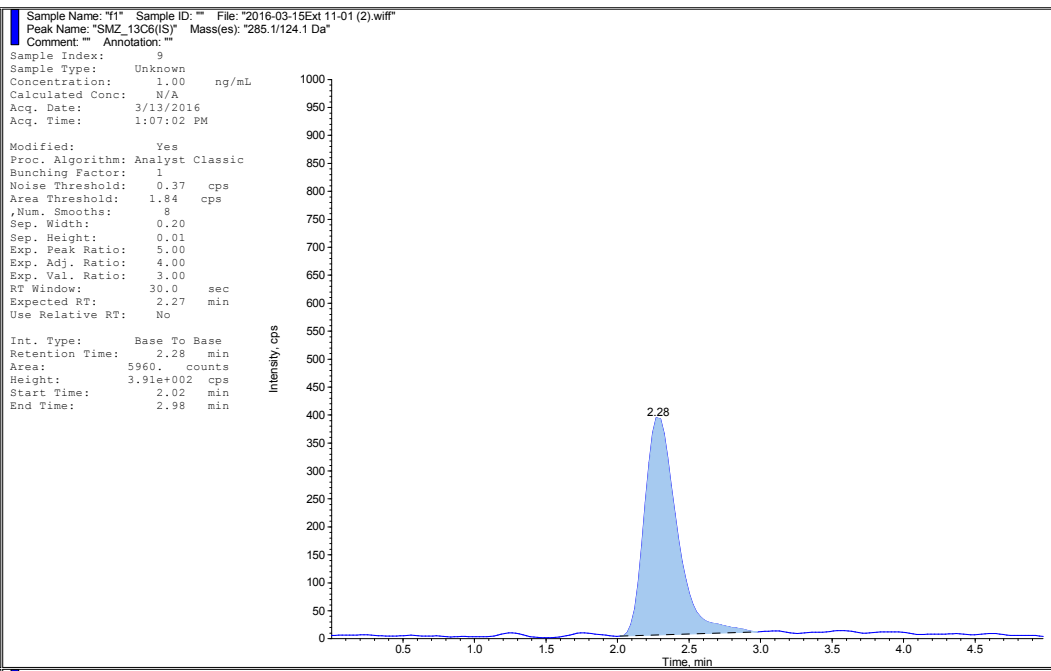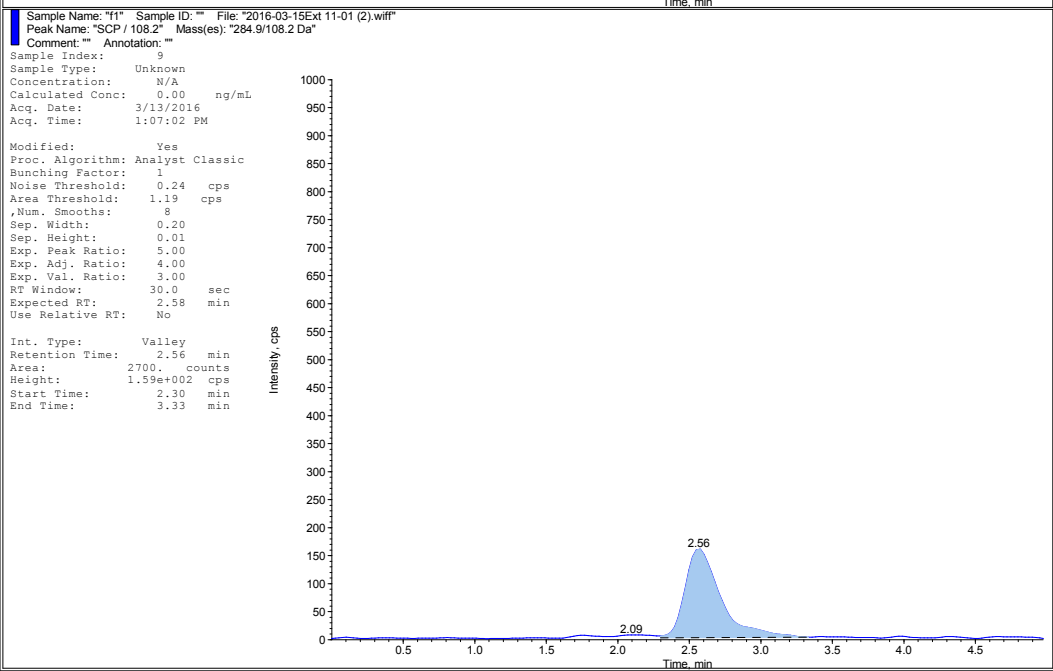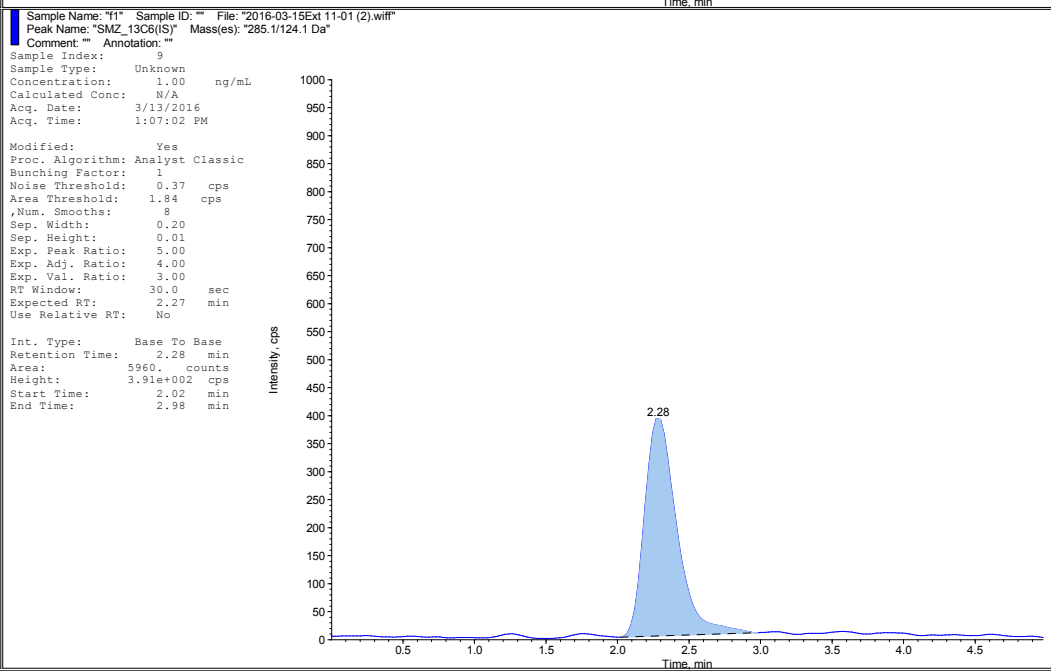

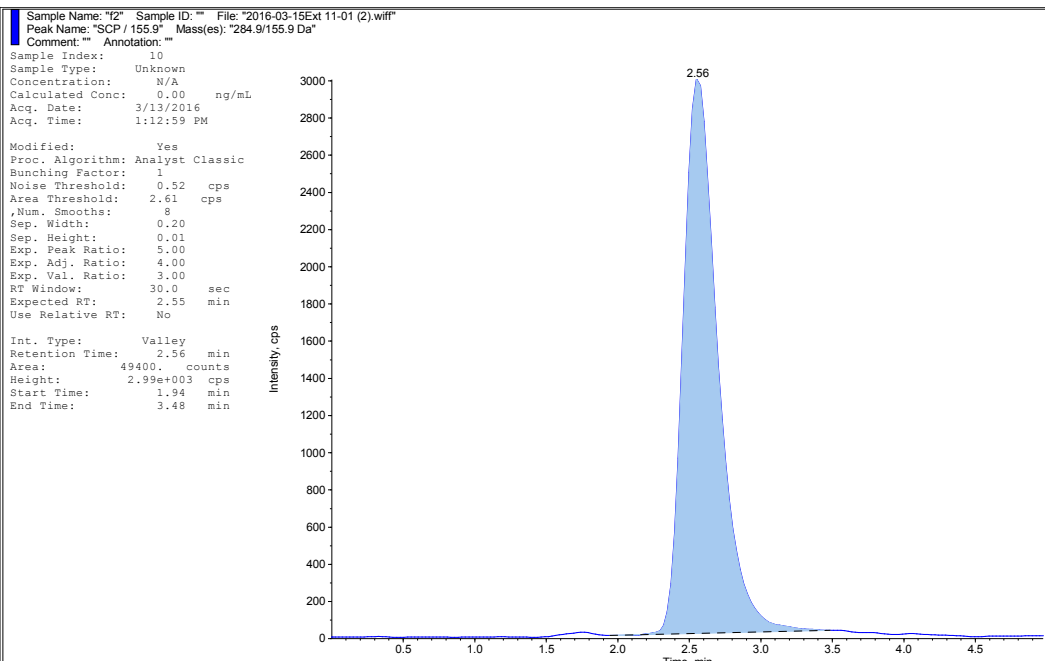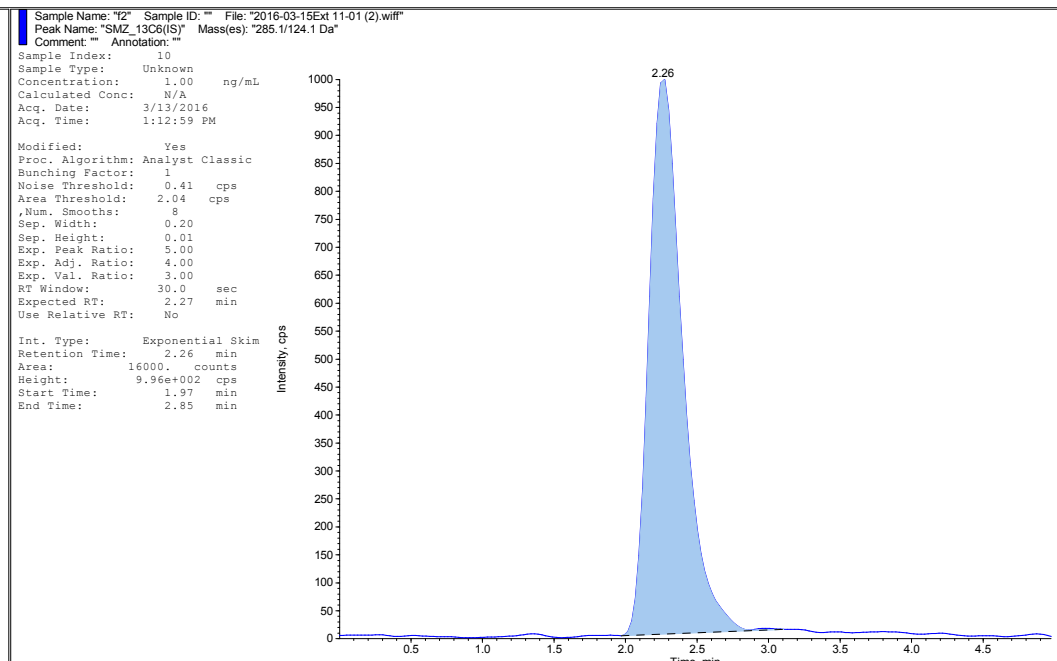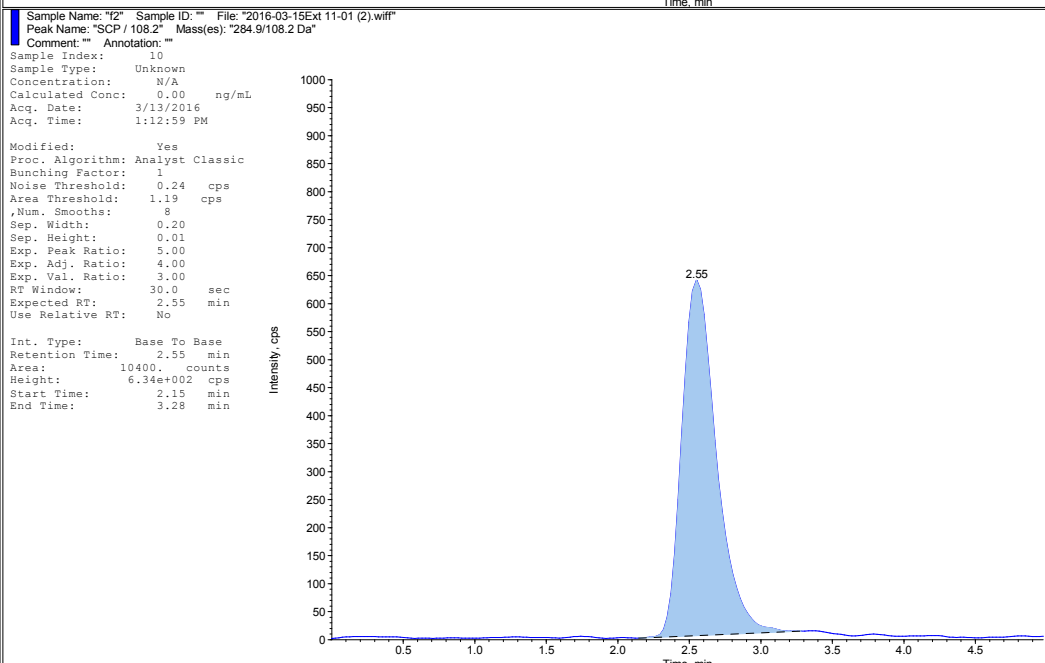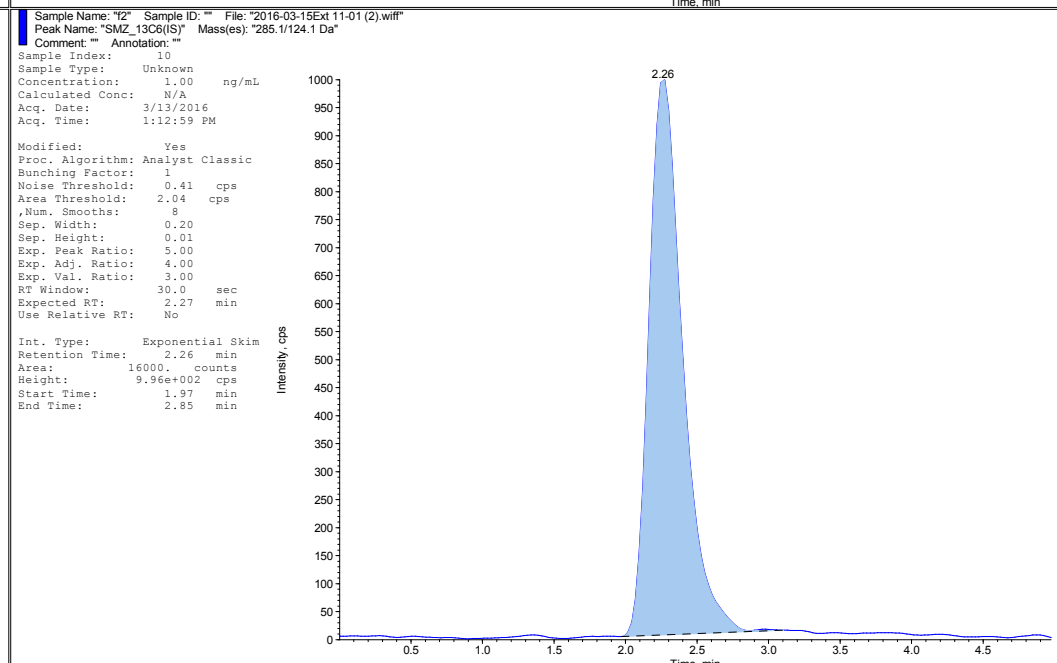

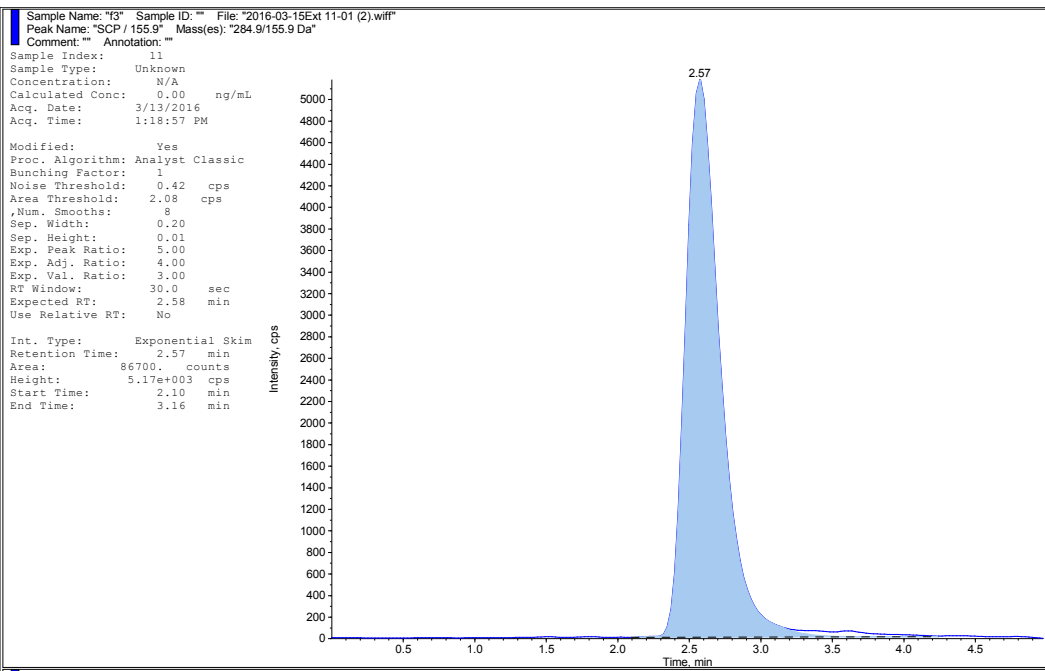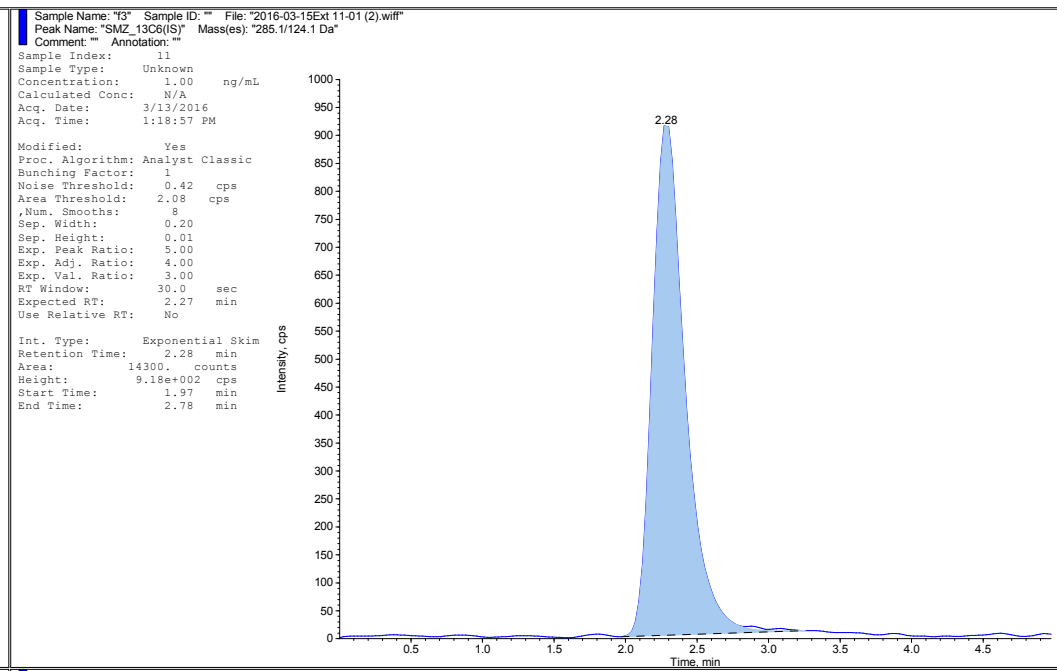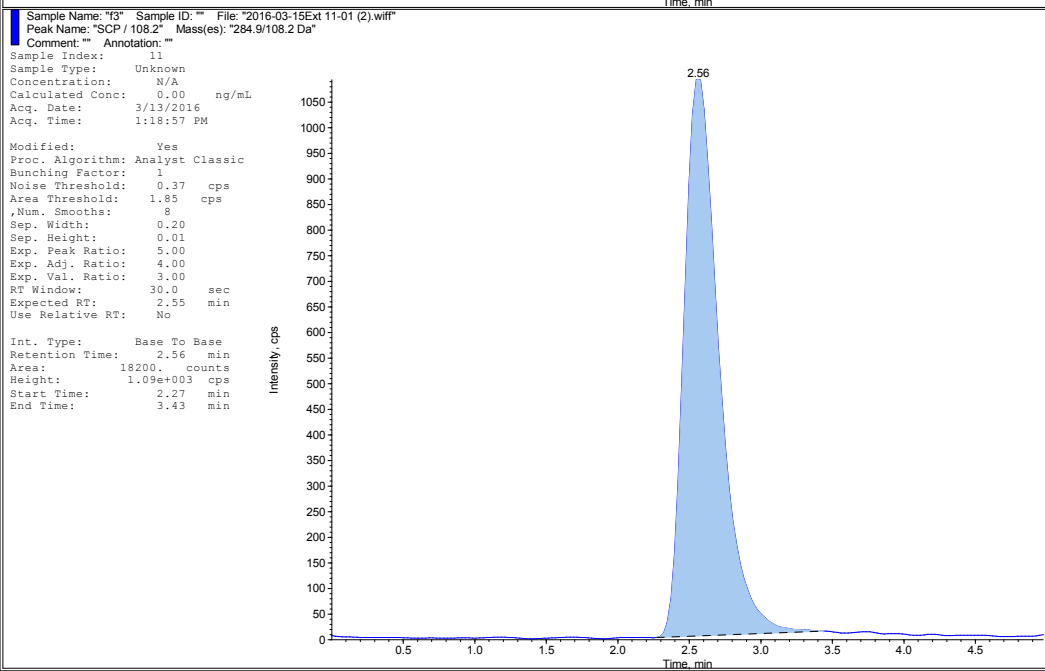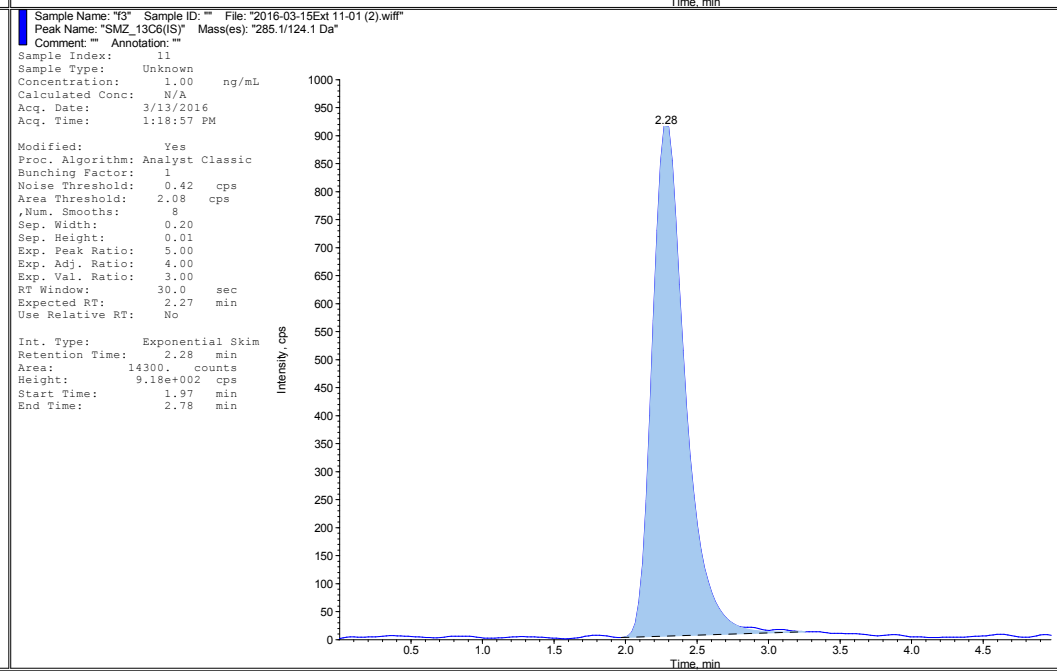

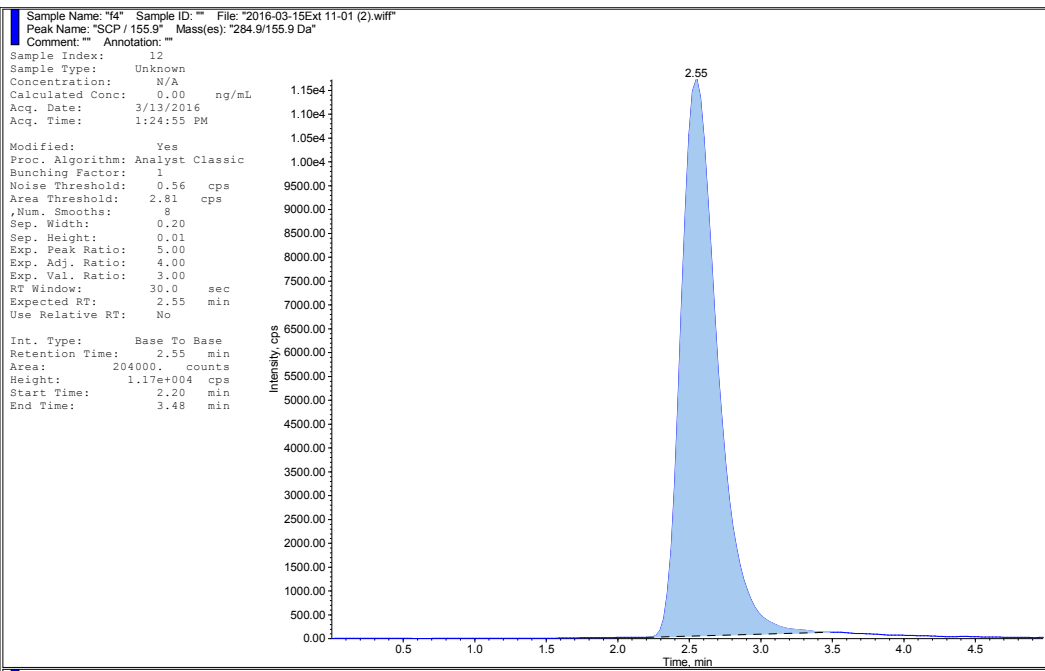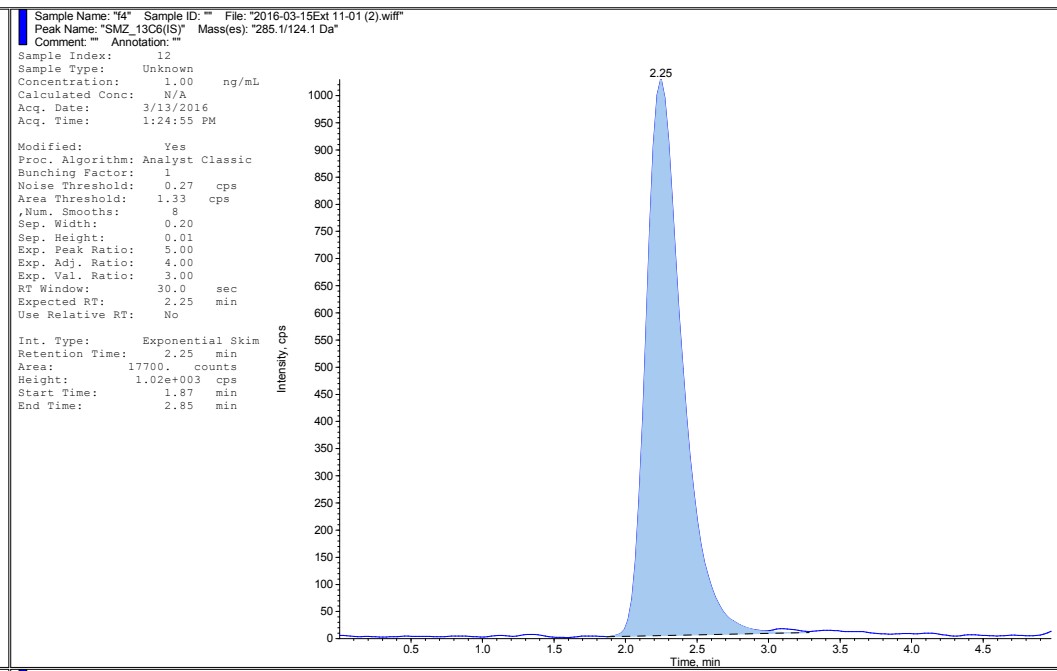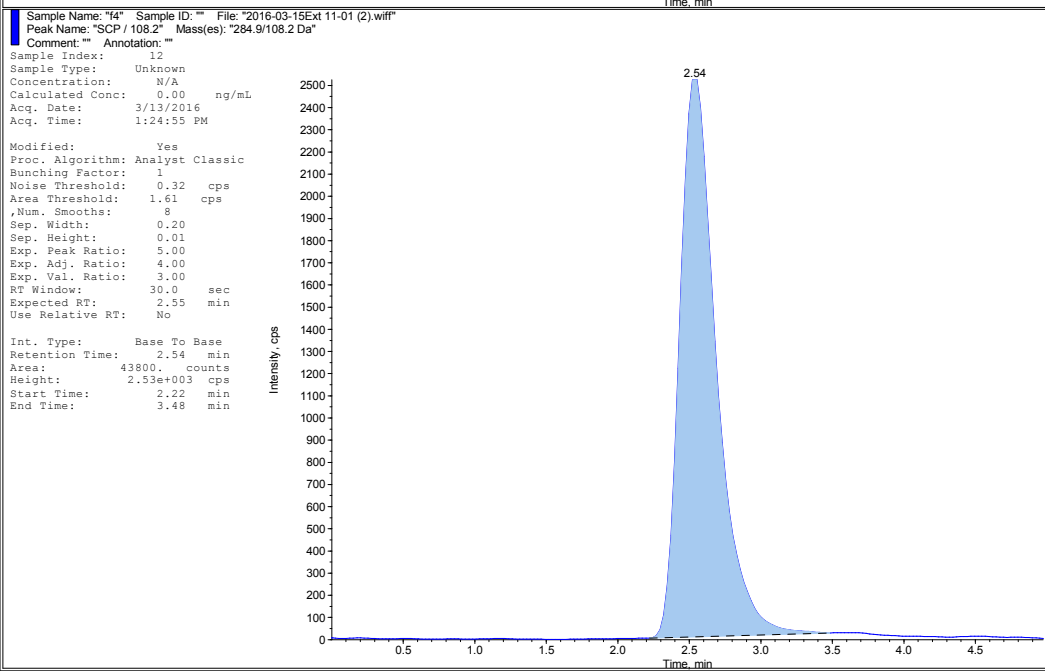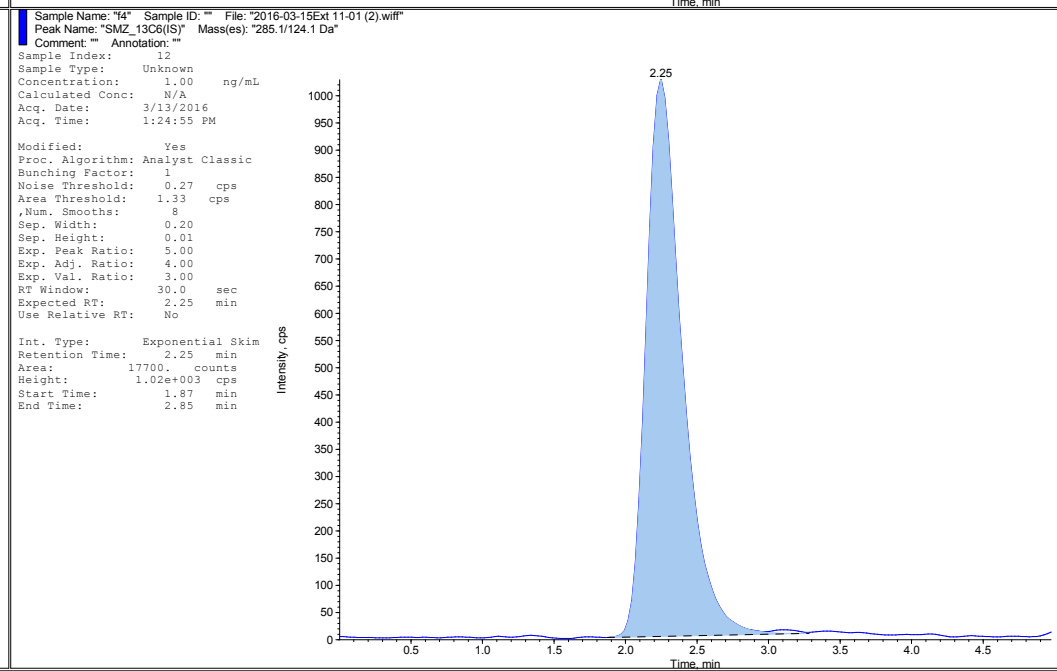

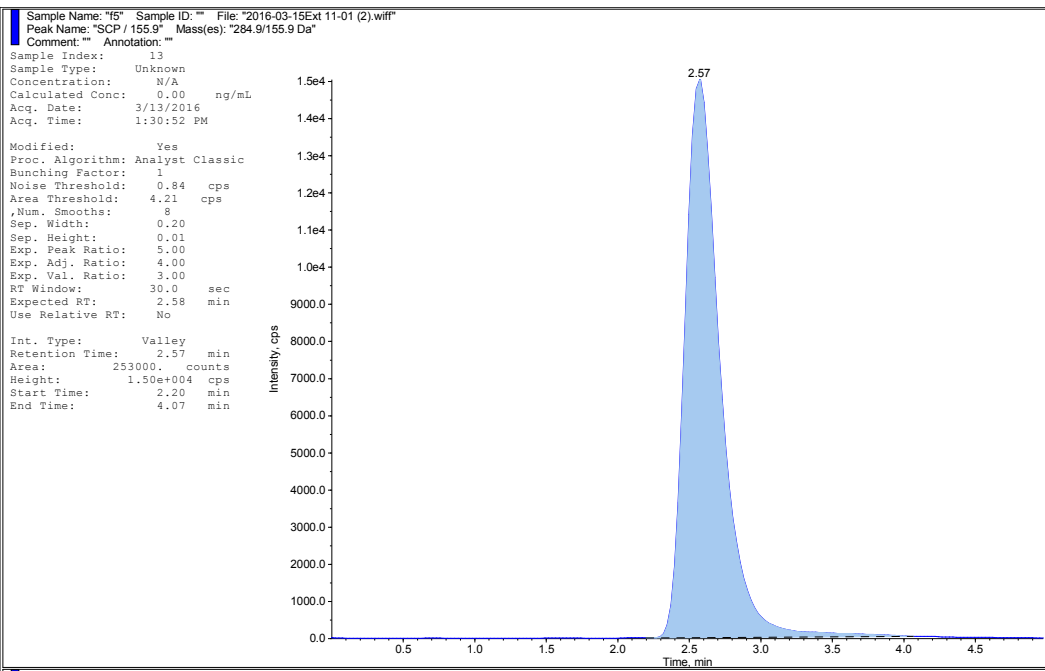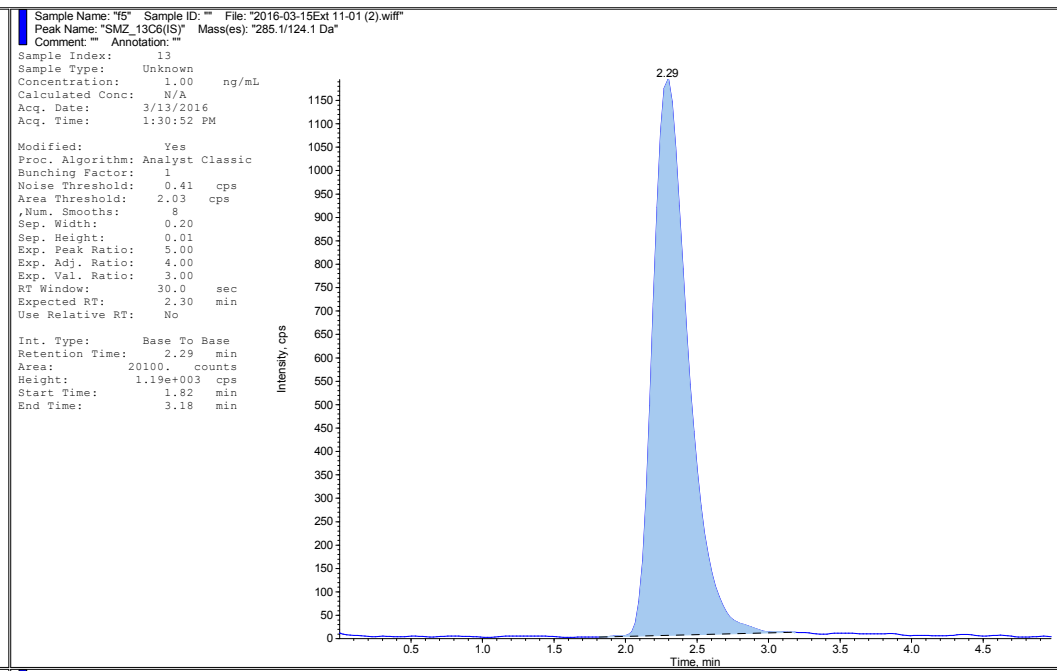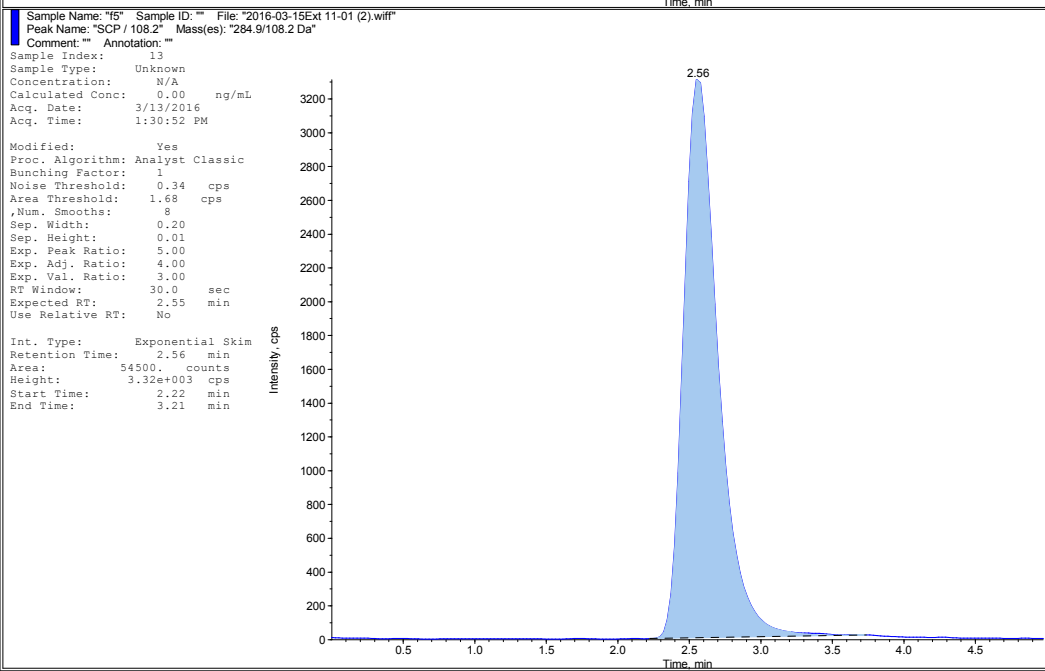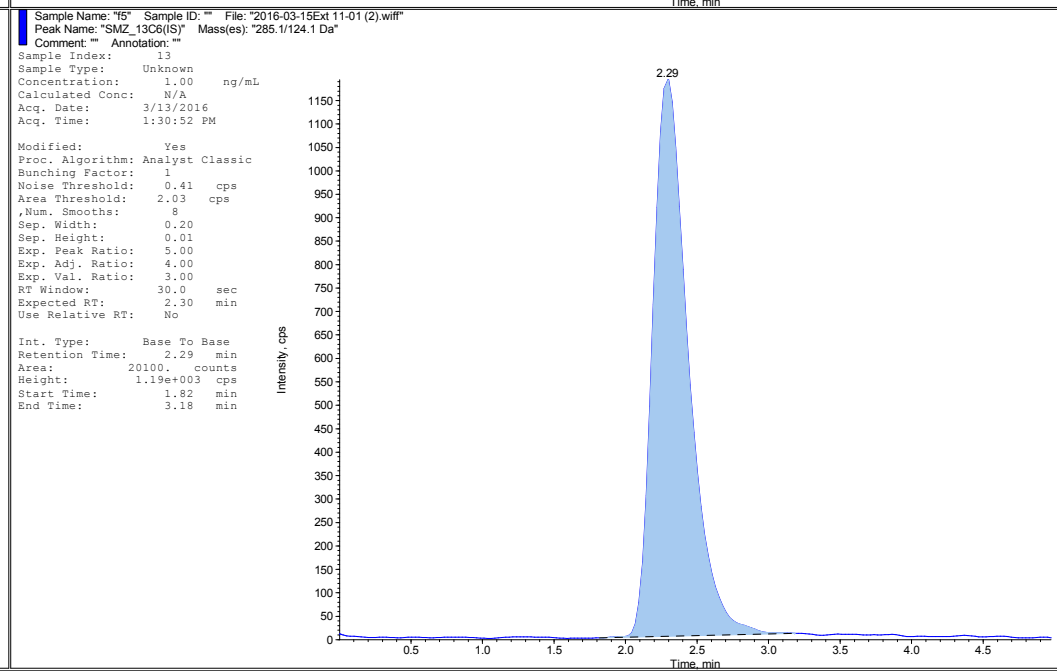

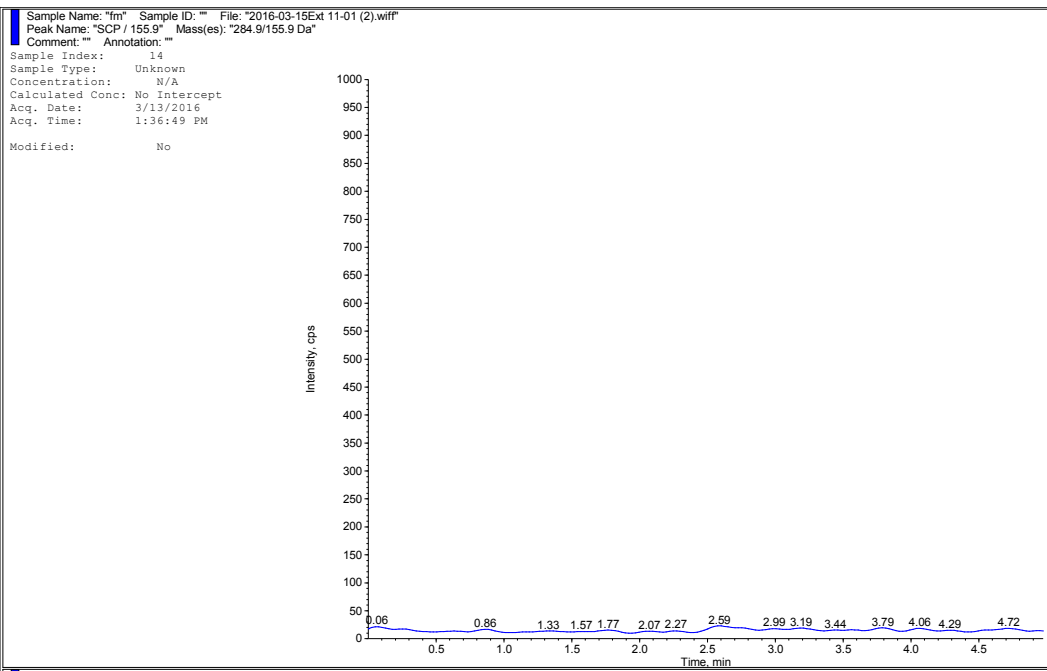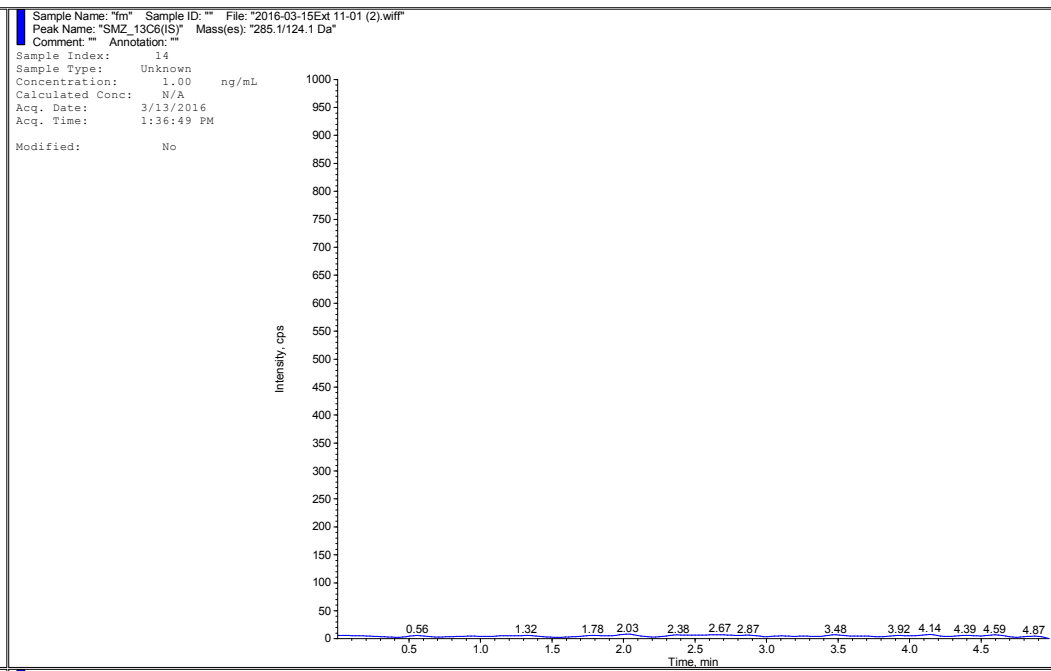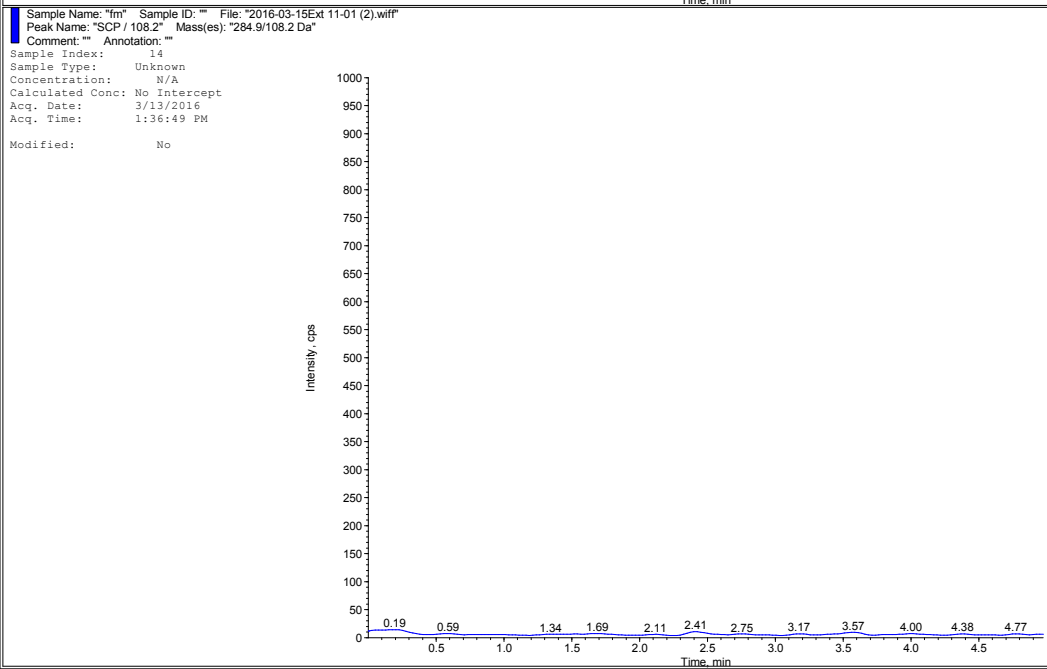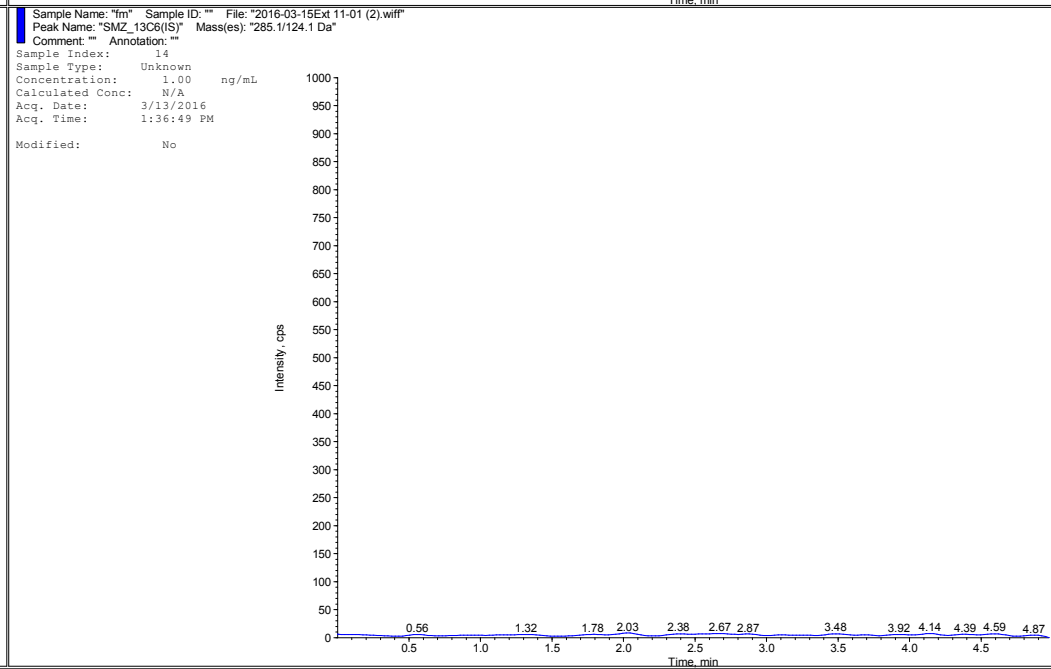

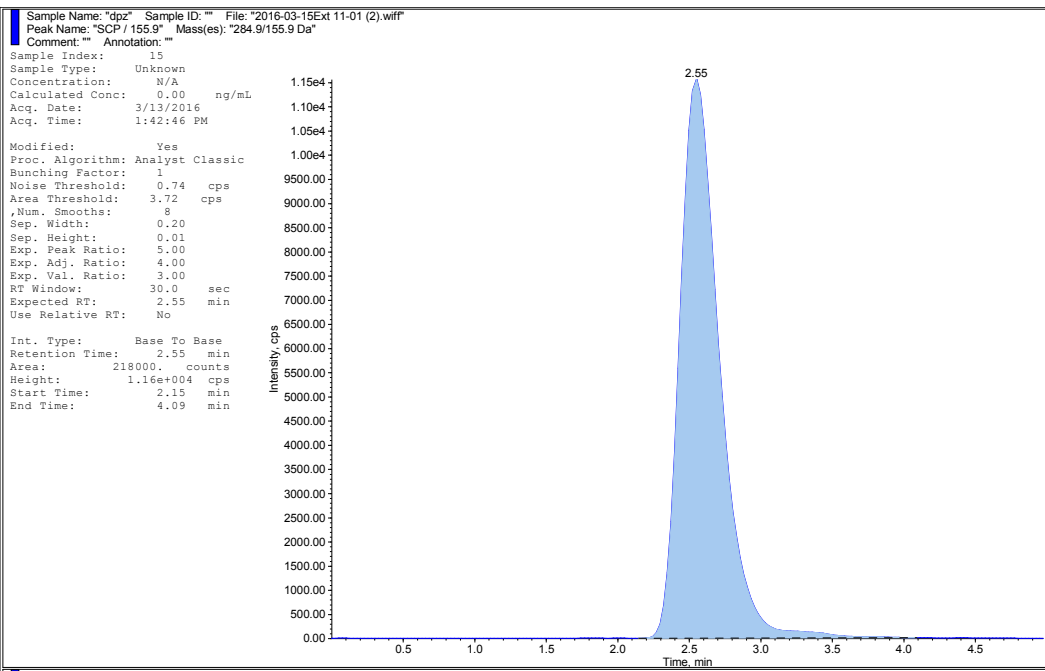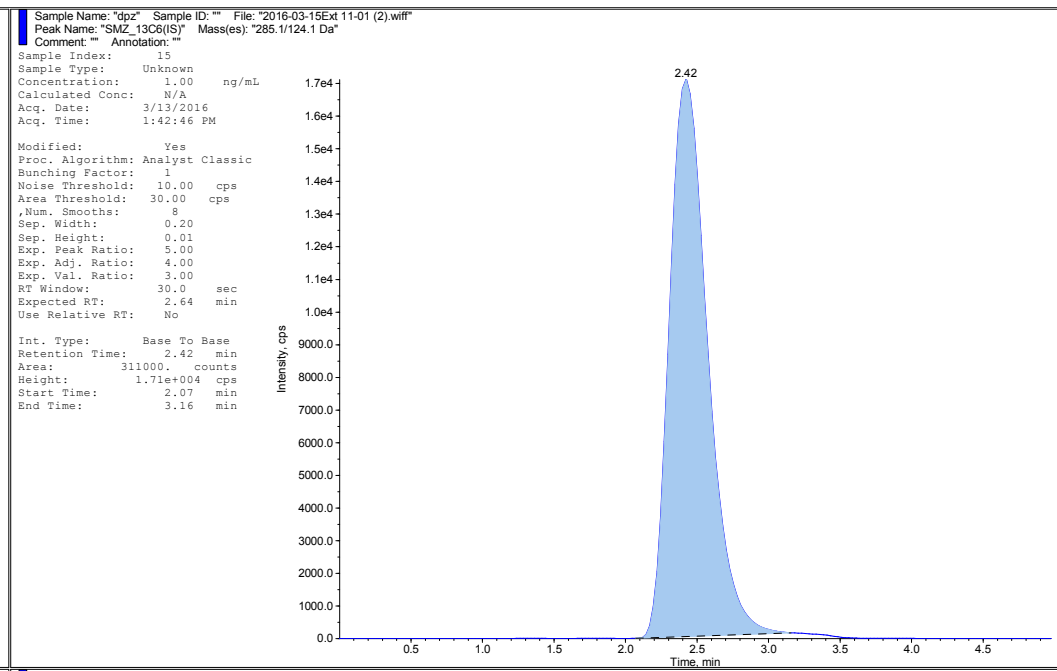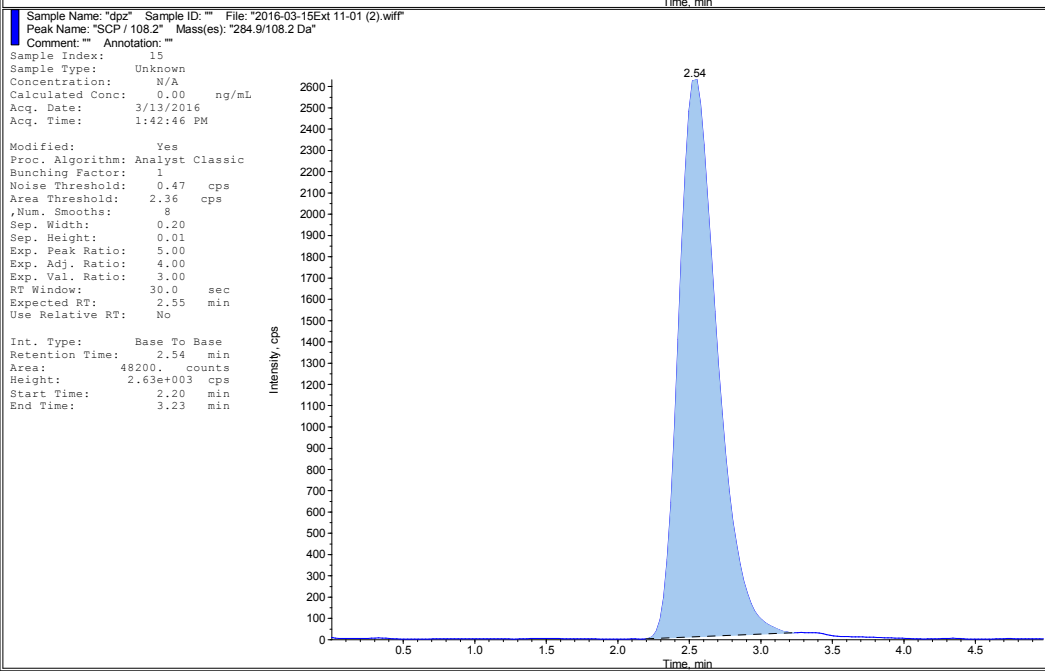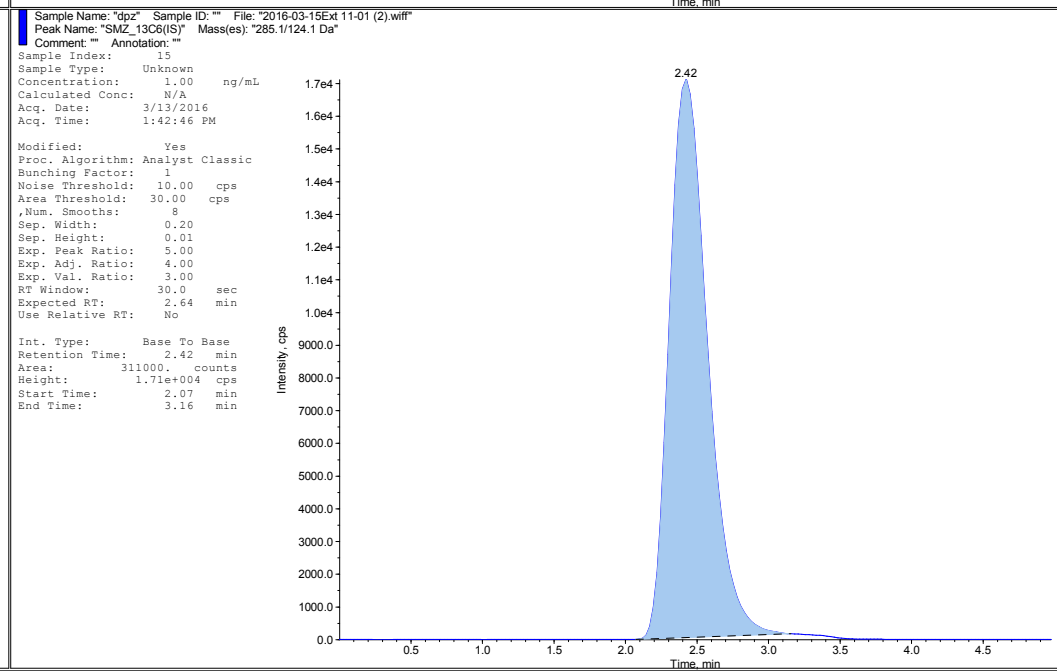

Supplement: S1 Fig — Fortified samples at 10, 20, 40, 80 and 100 μg Kg-1, as well as the positive and negative controls (blank sample to sulfacholopyridazine). Sulfamethazinephenyl-13C6 hemihydrate (SMZ-13C6) as internal Standard at a concentration of 4.8 μg Kg-1. (PDF) [file pone.0200206.s001.pdf]

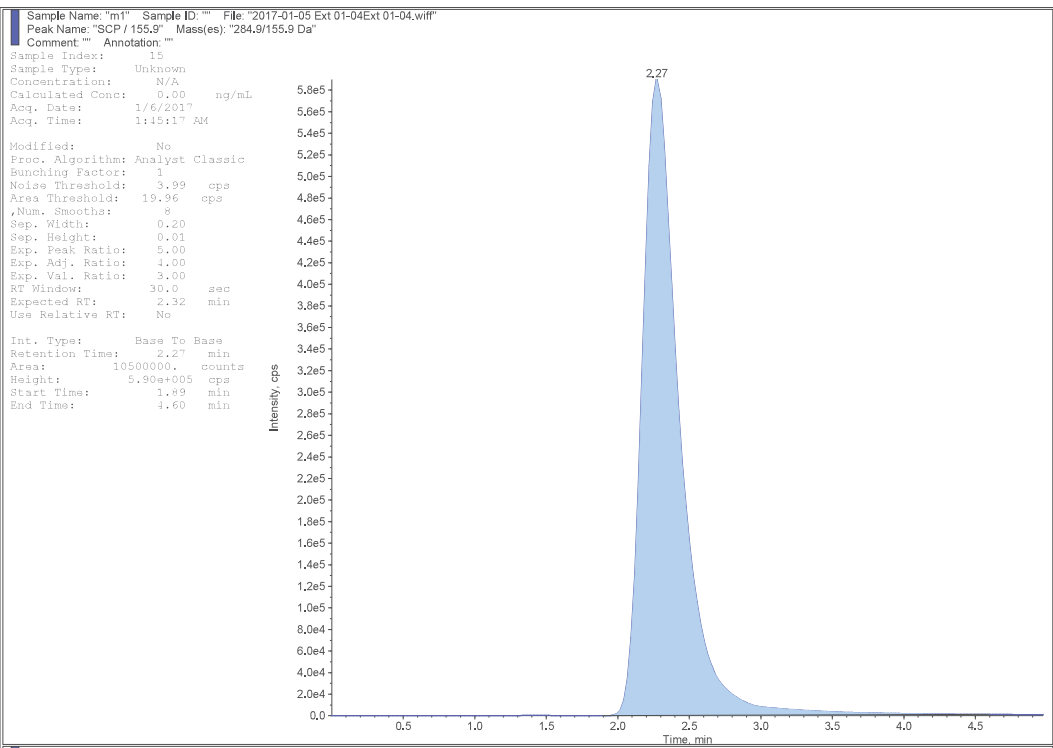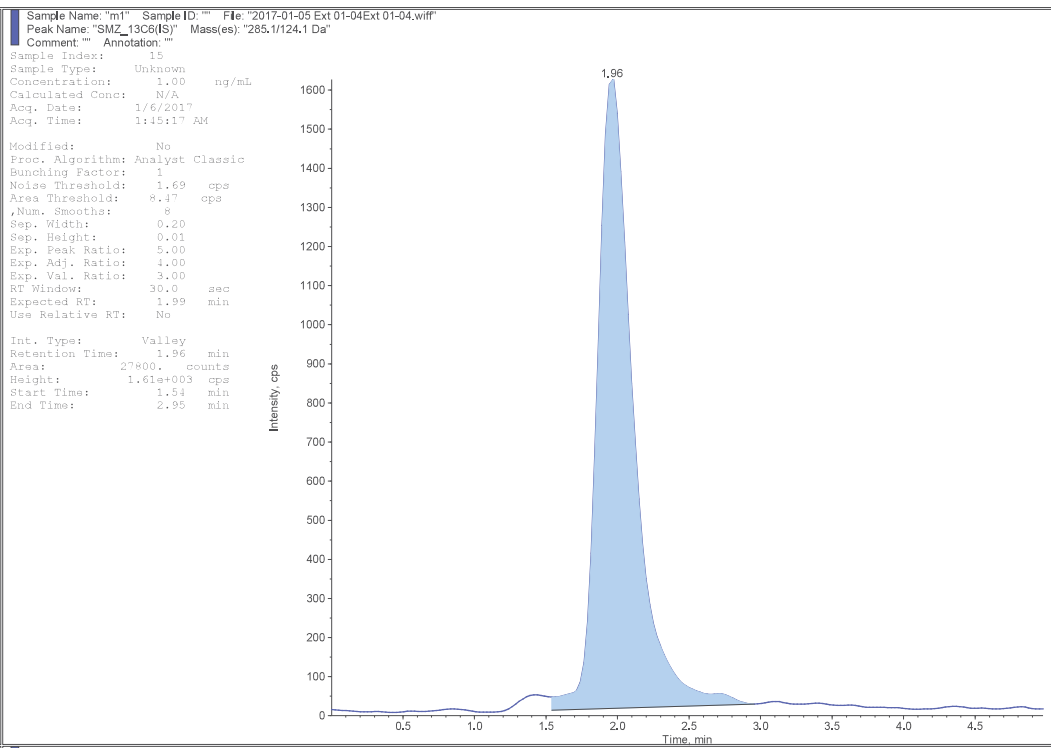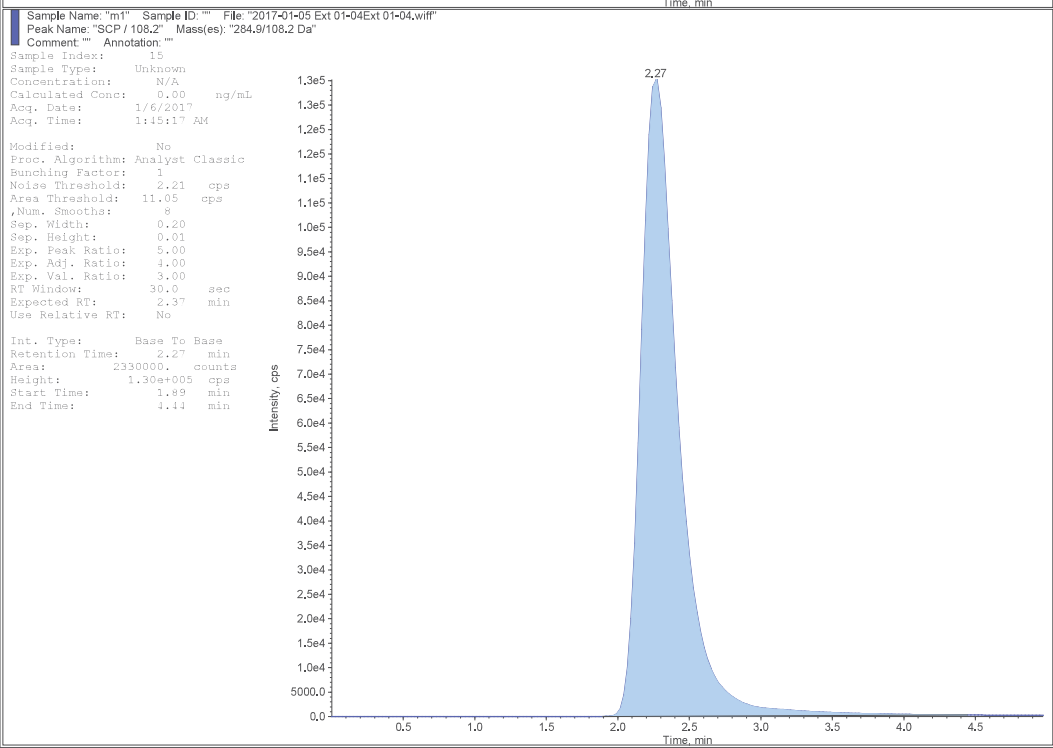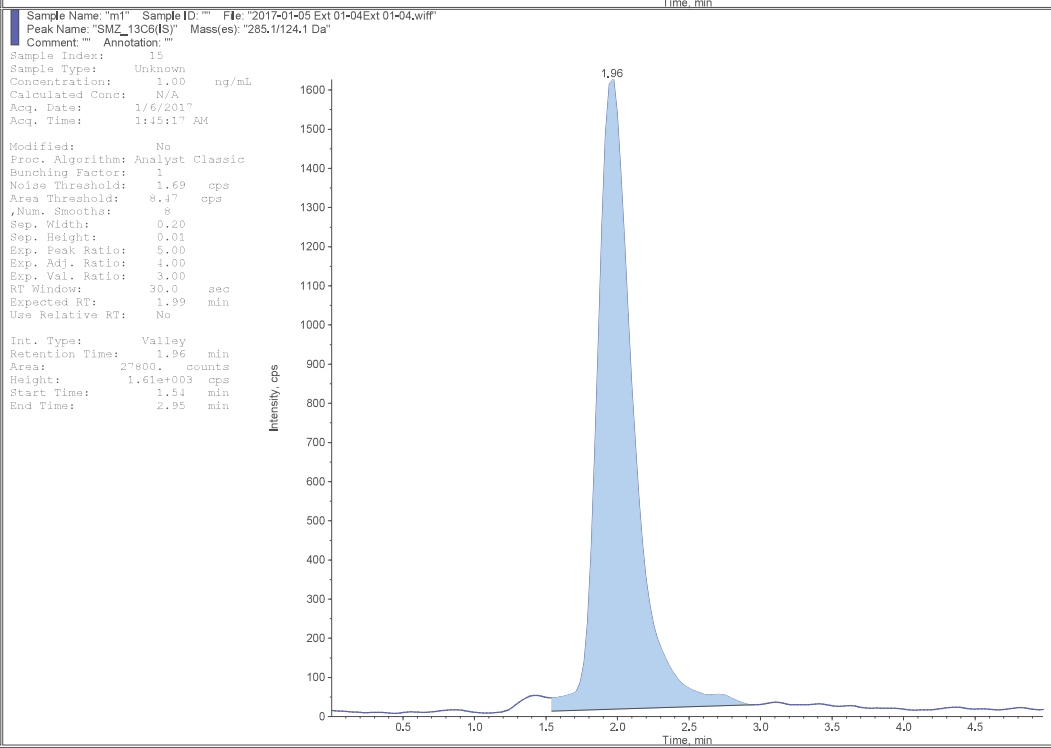

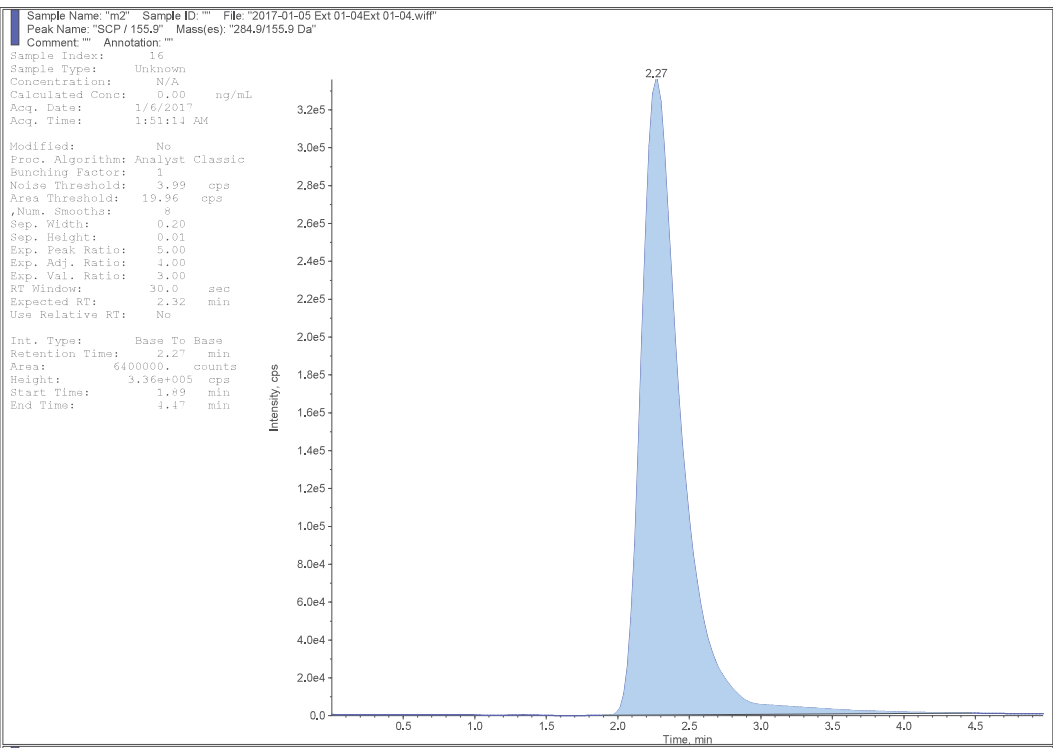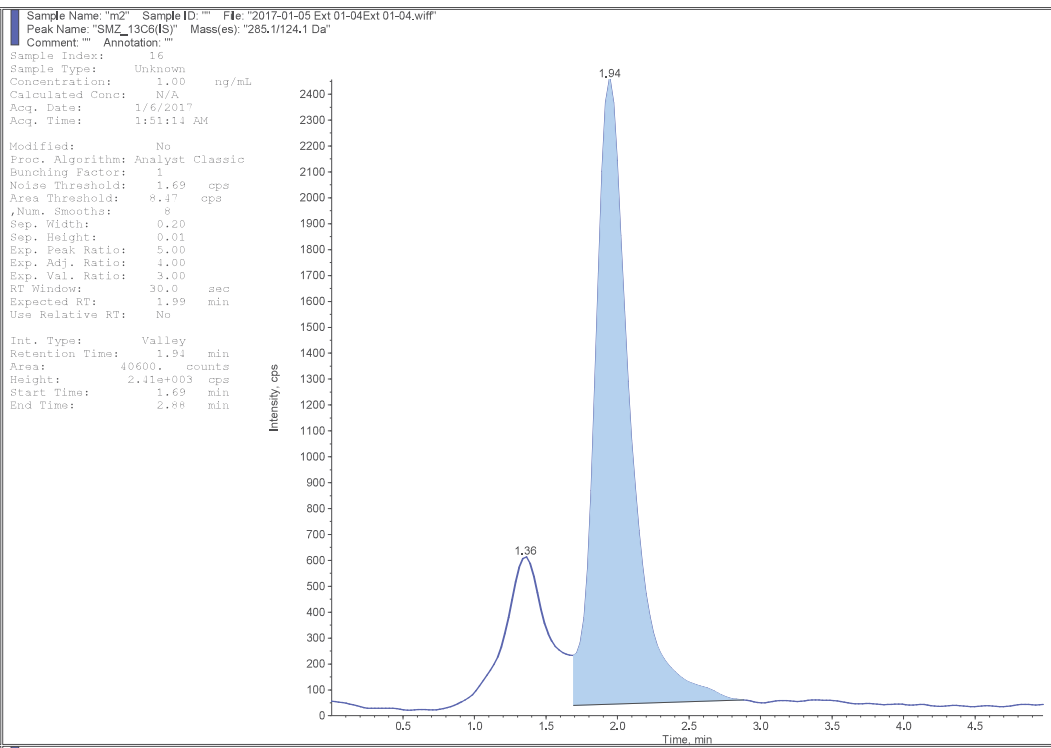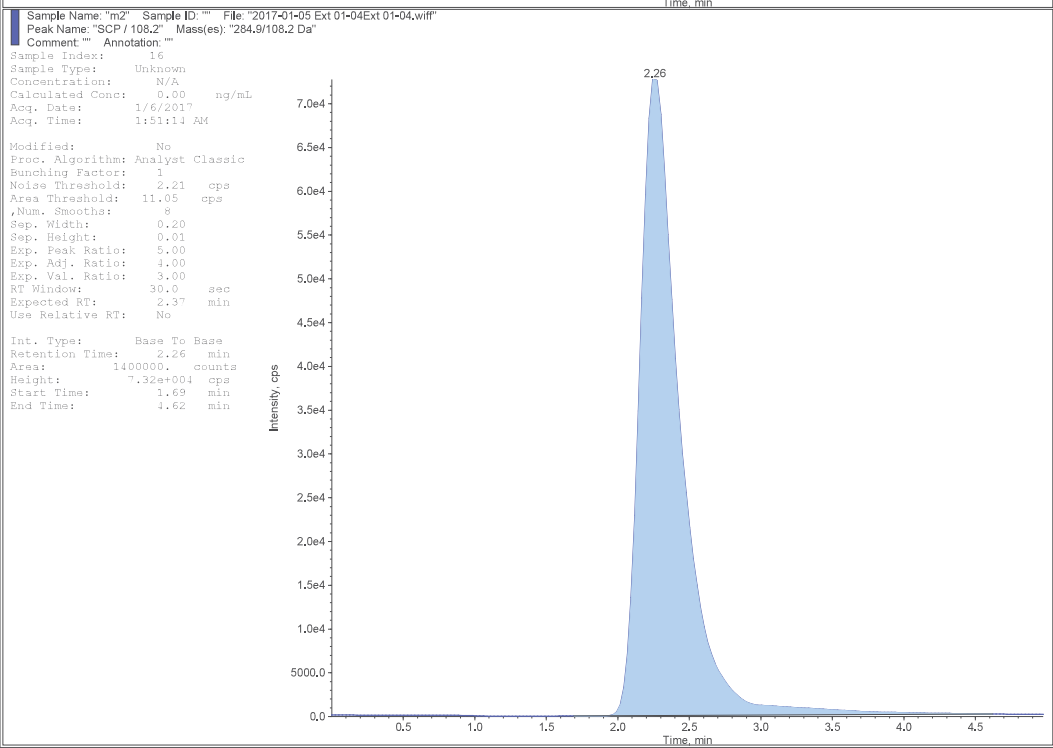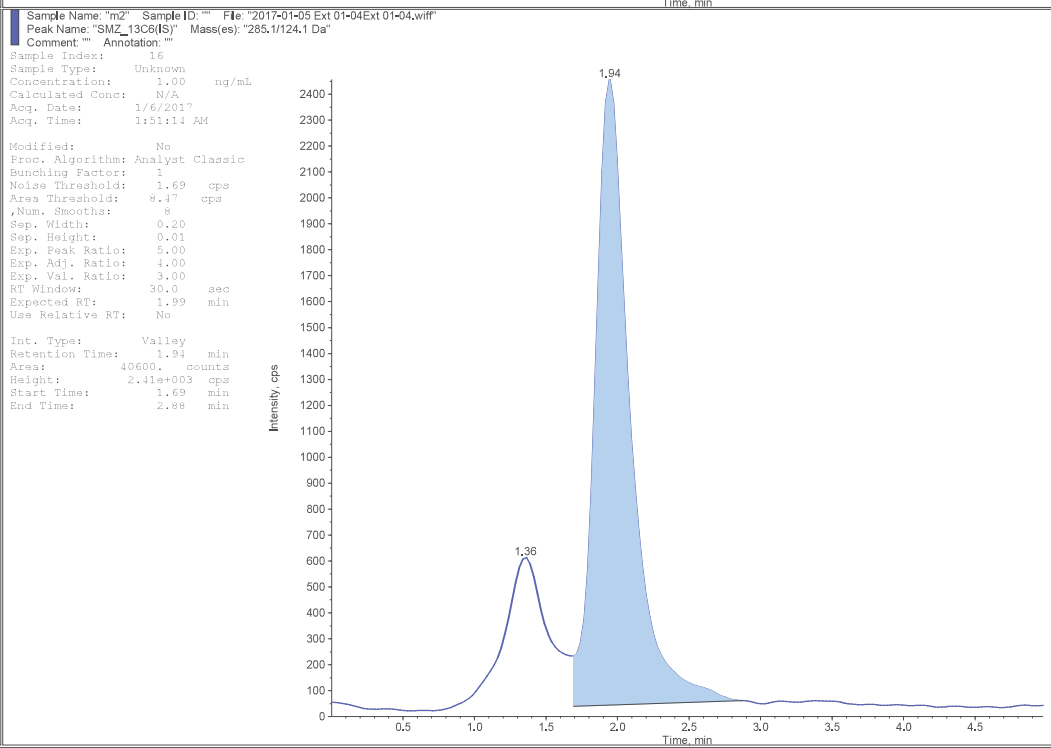

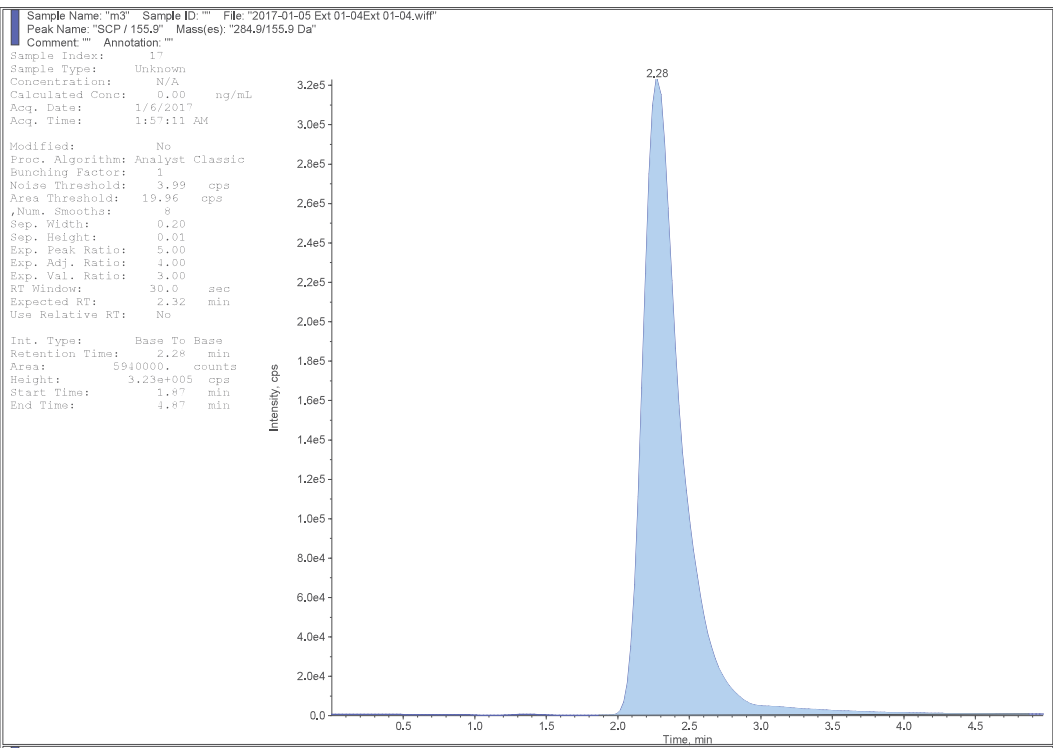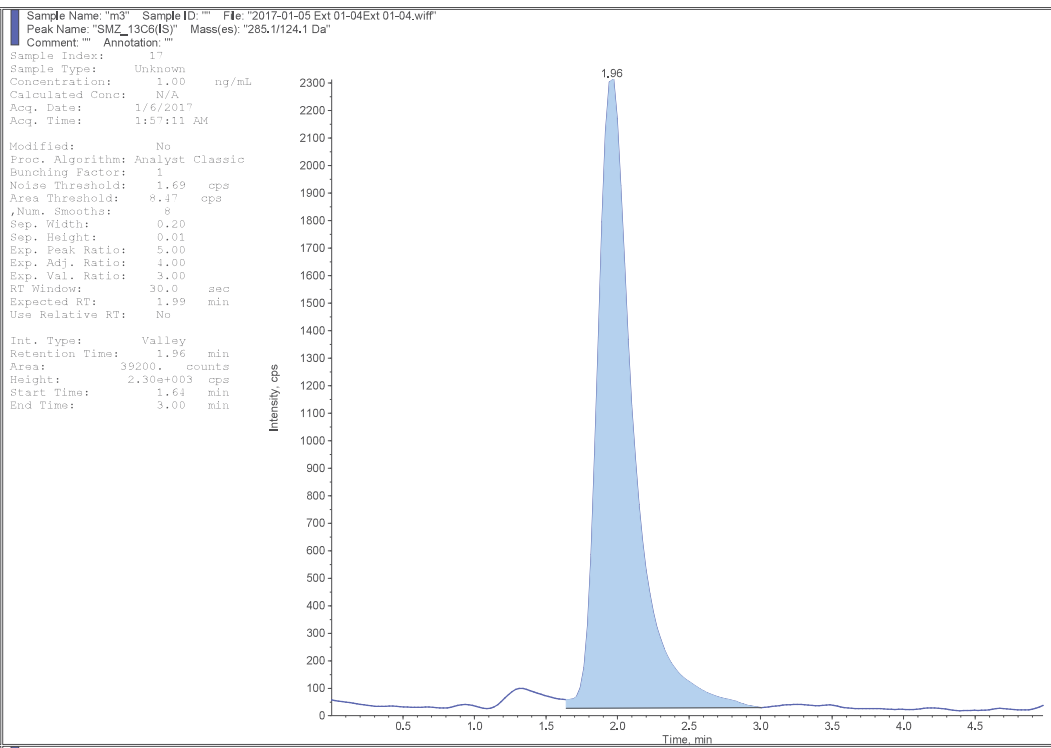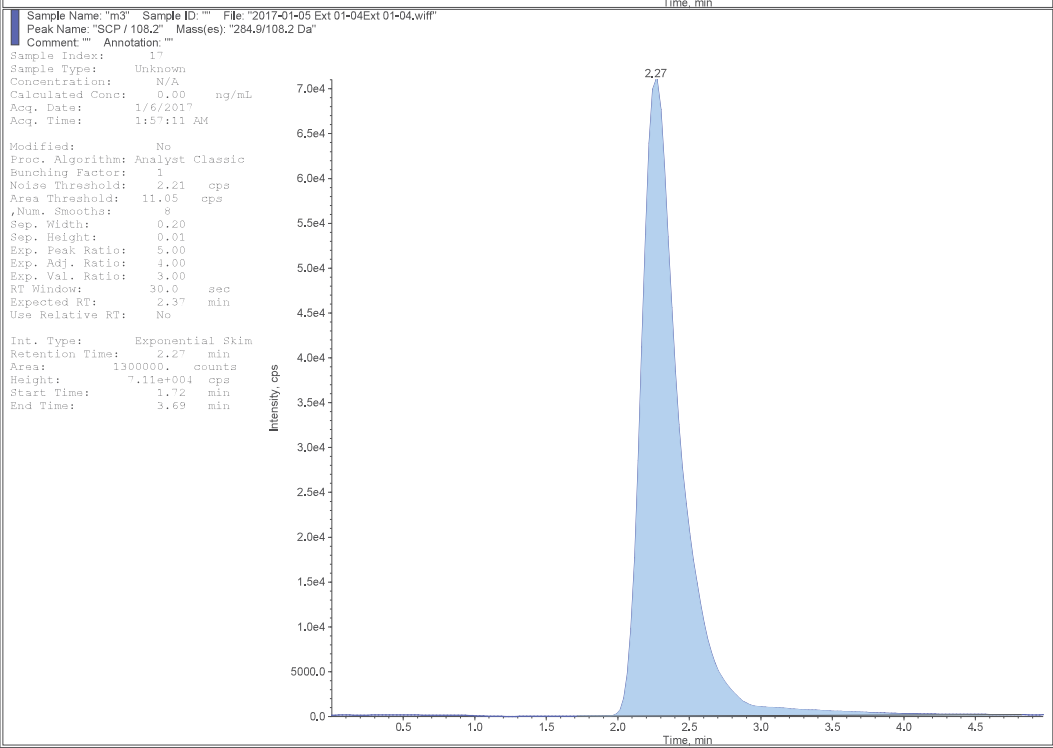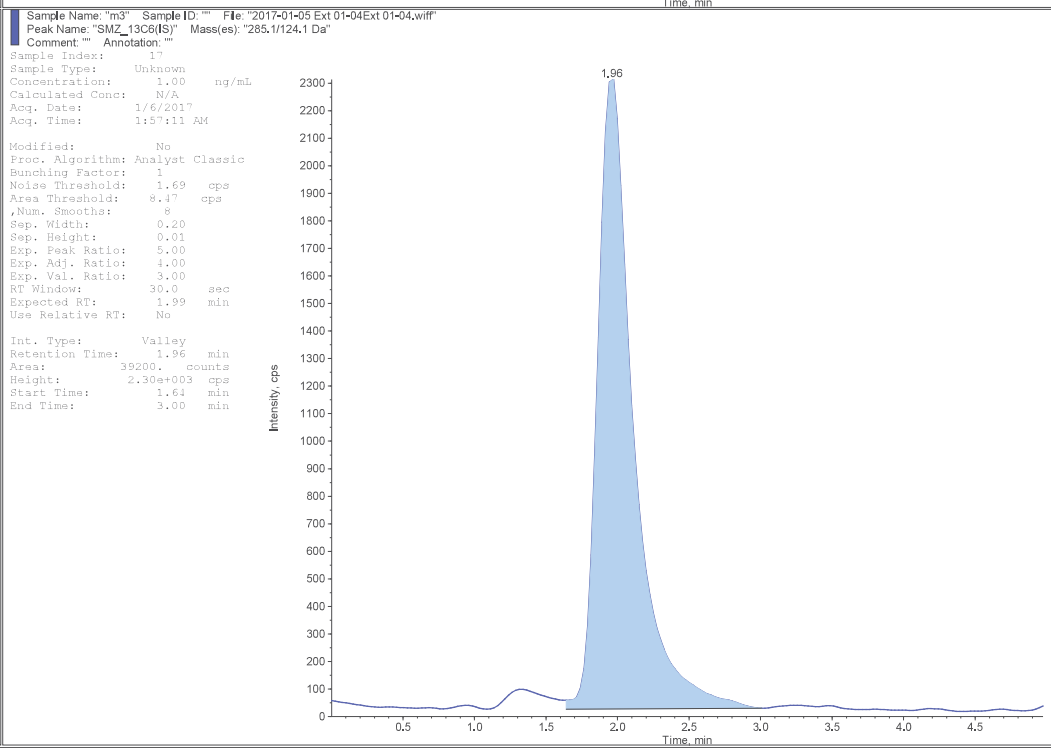

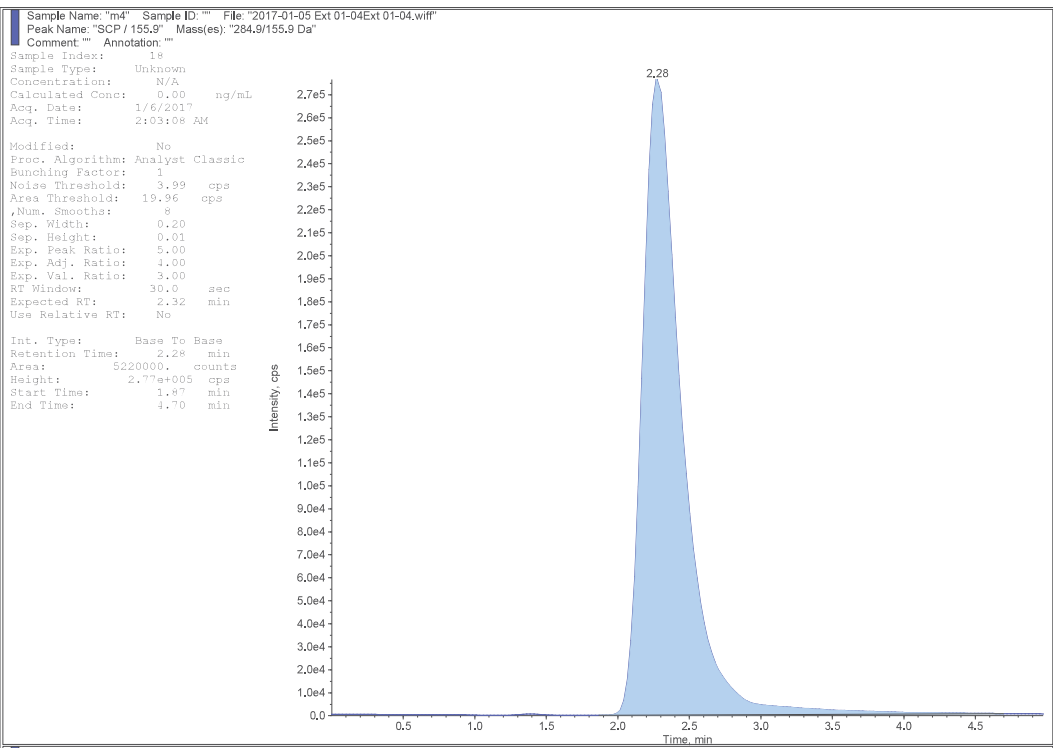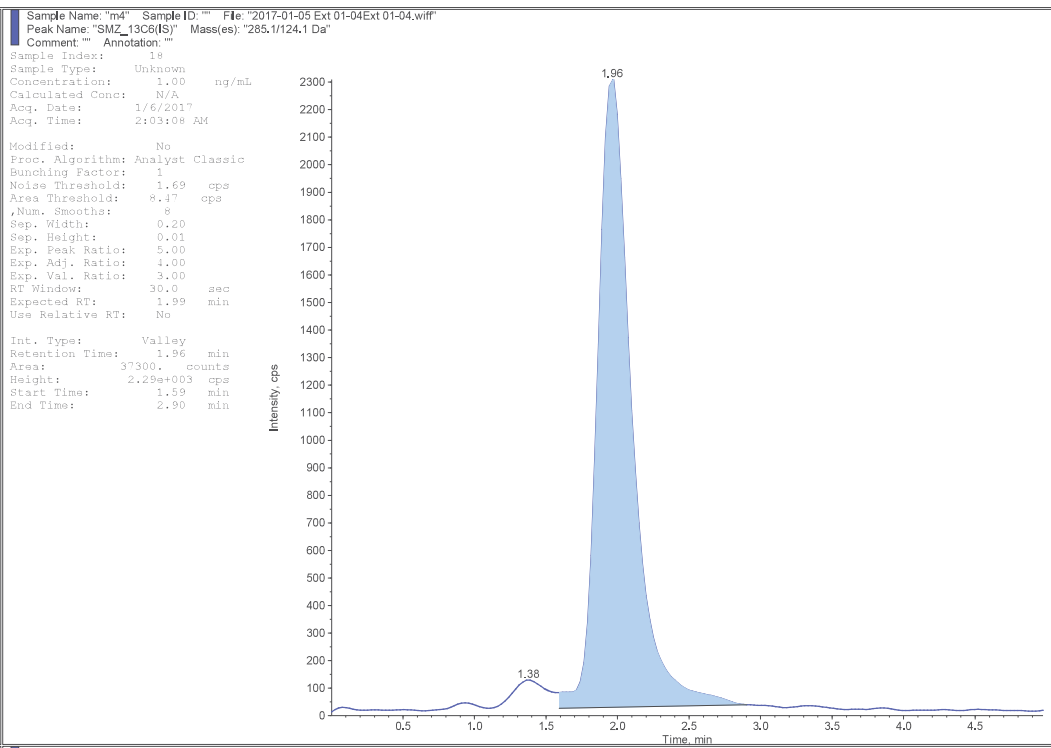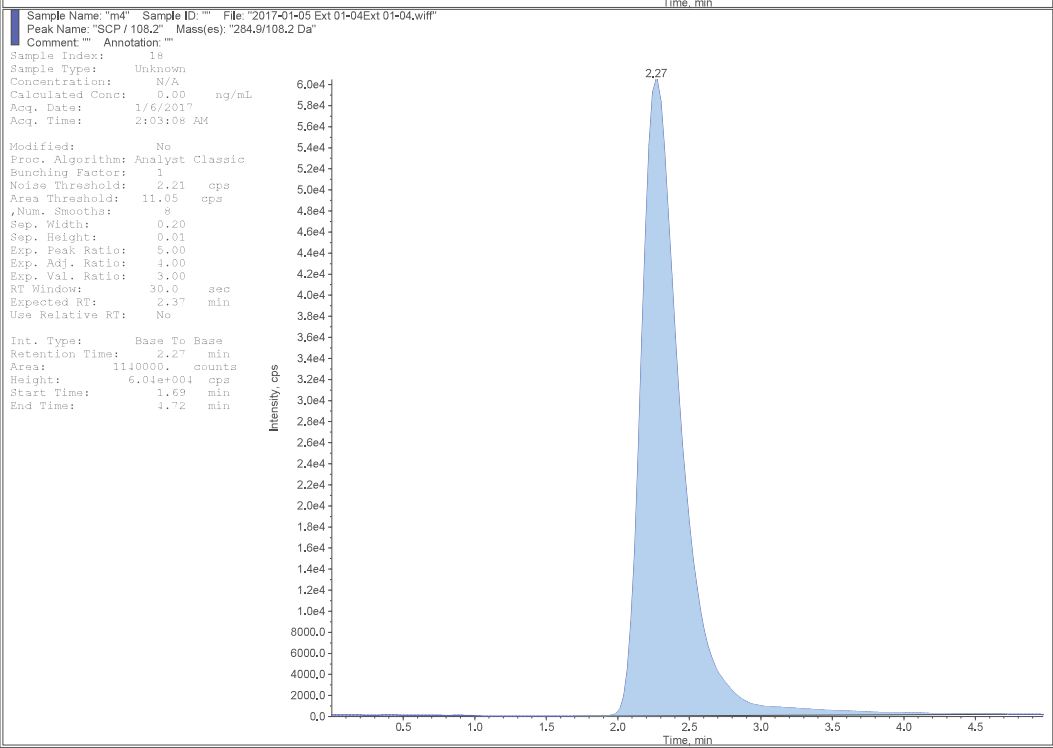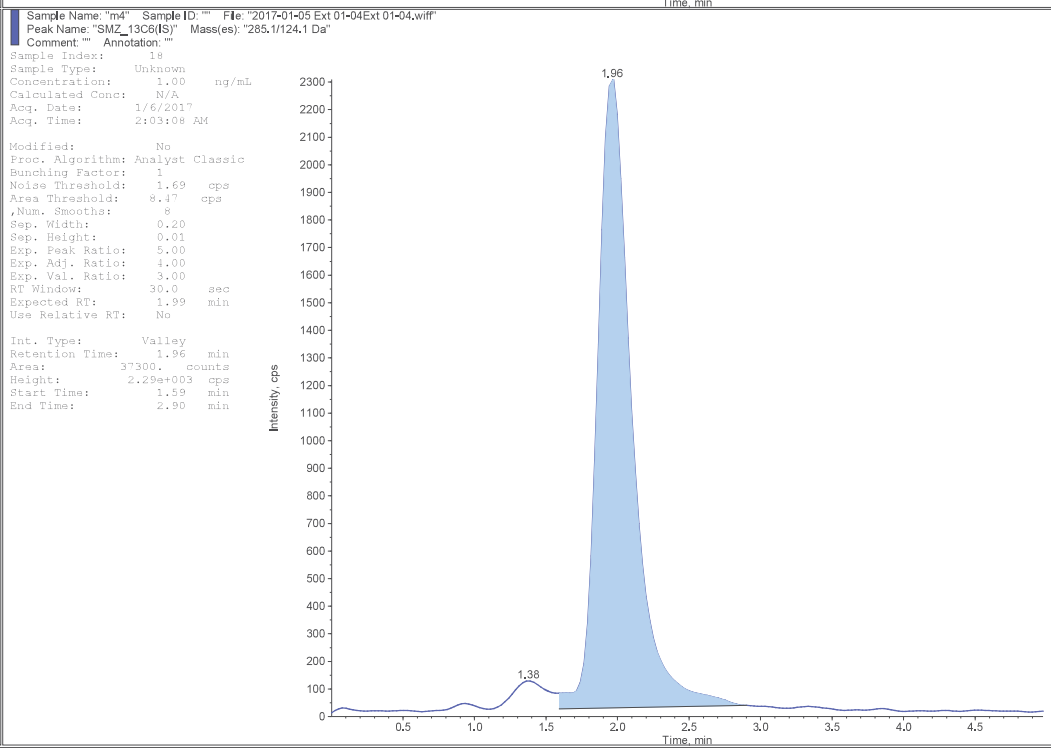

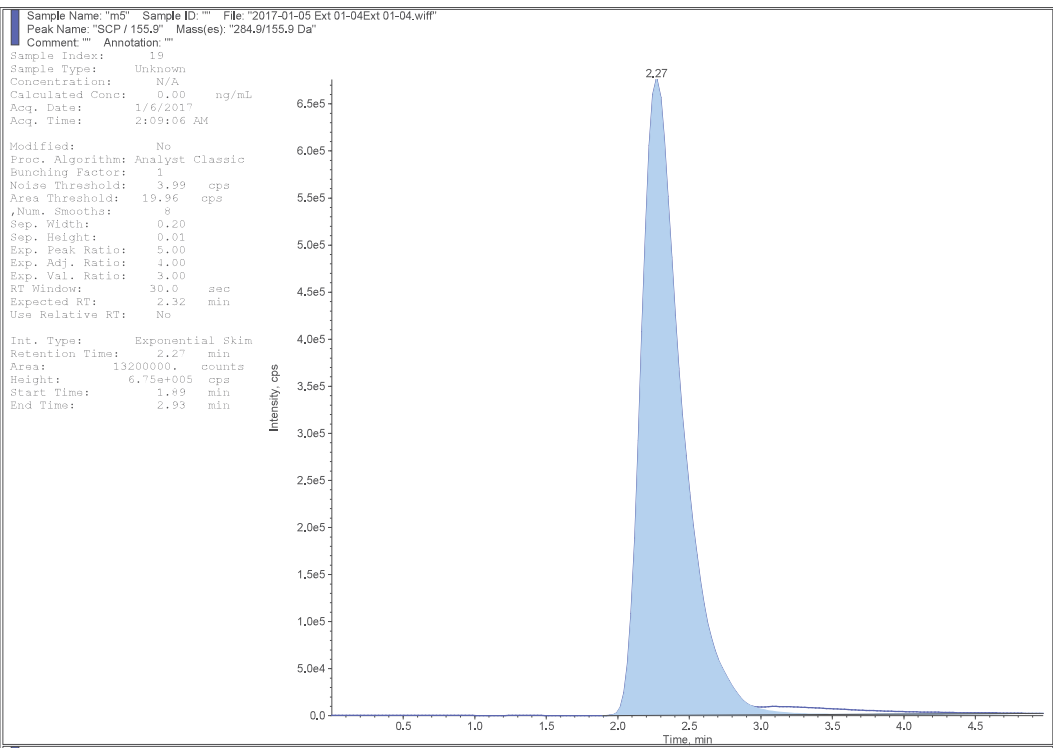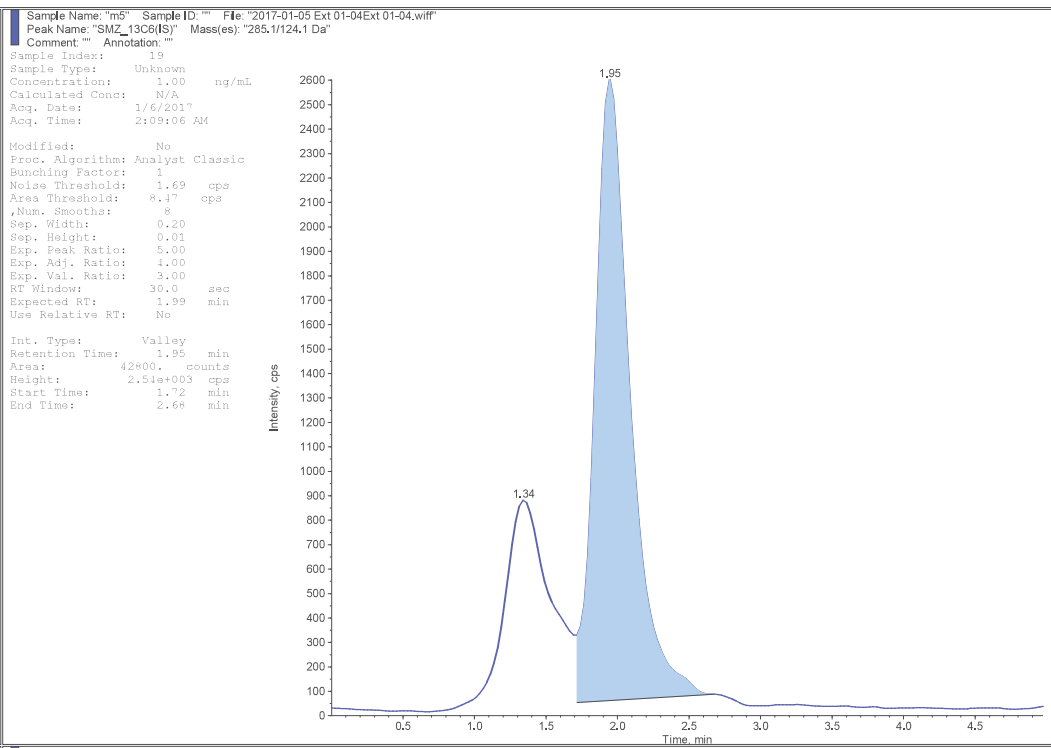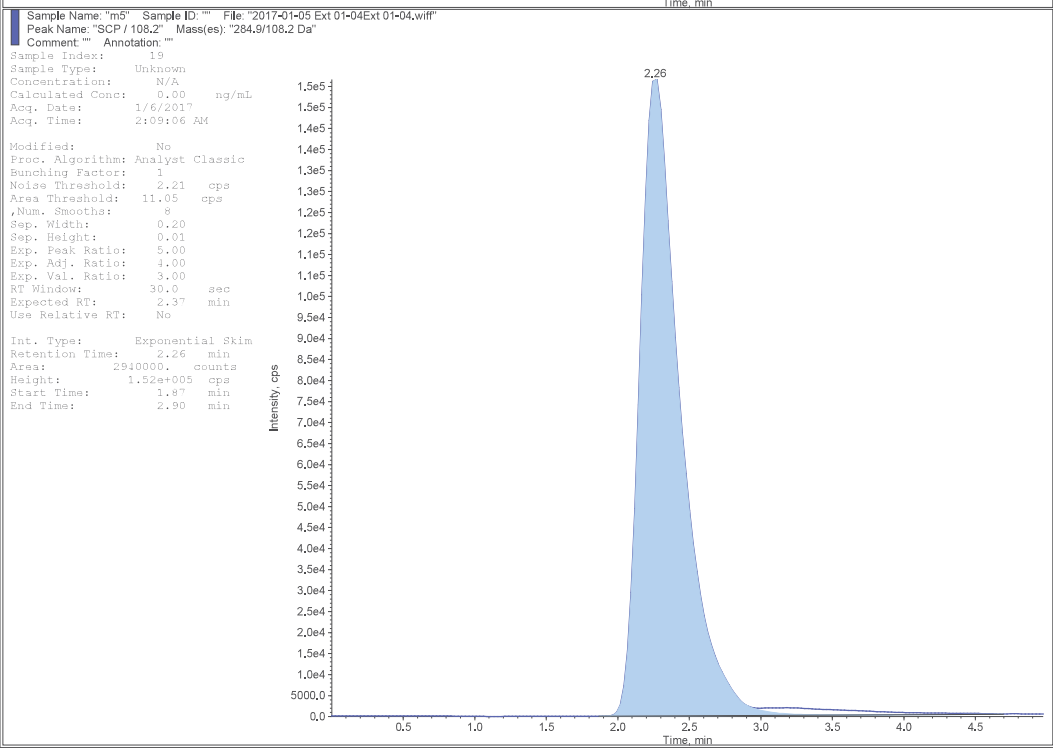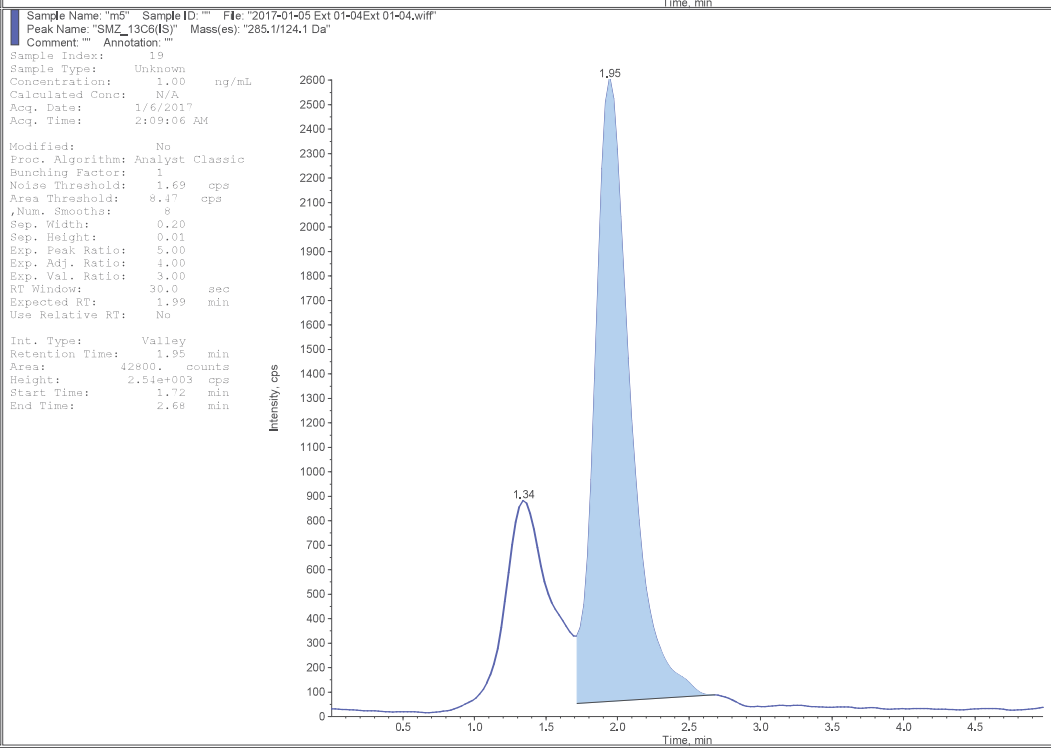

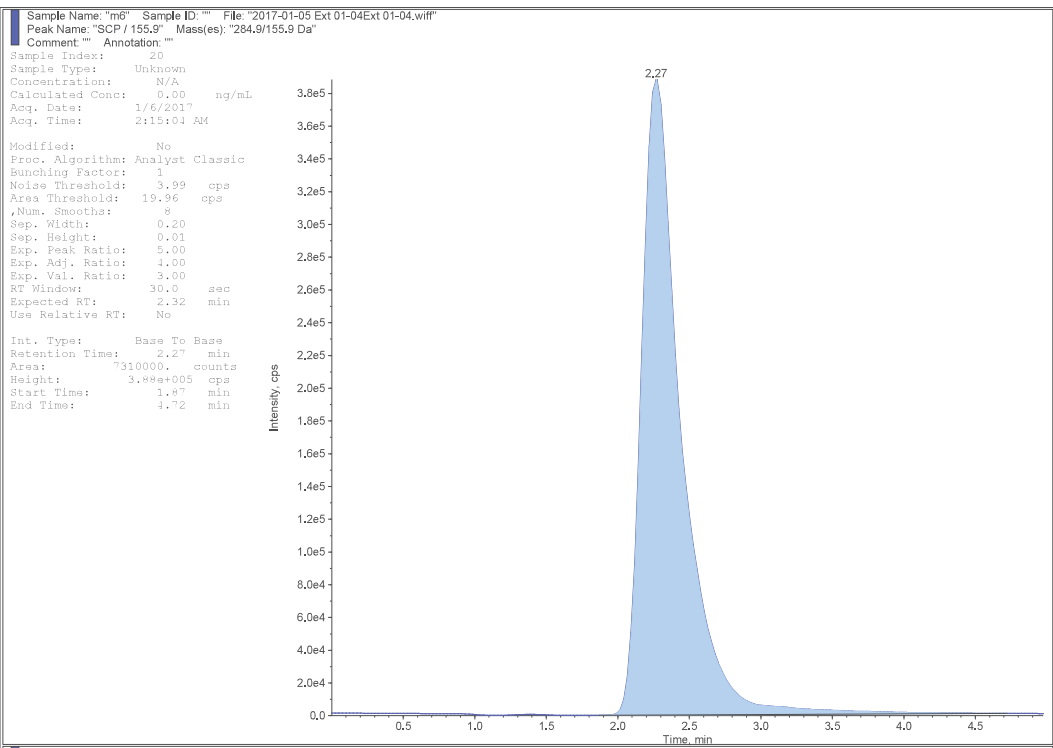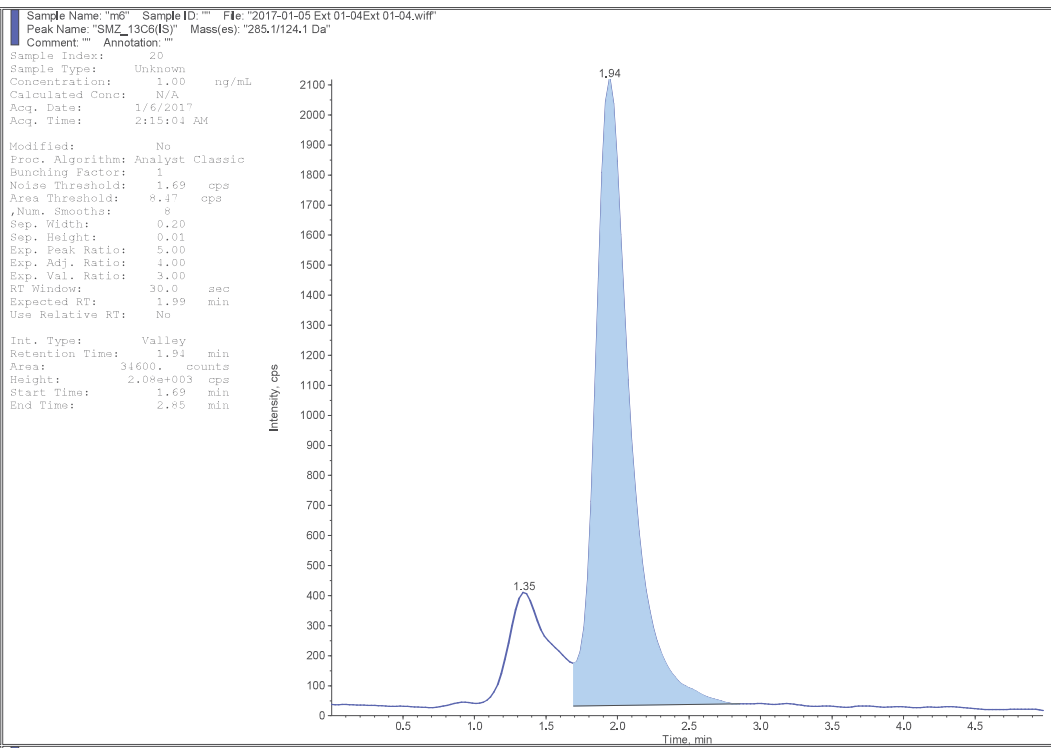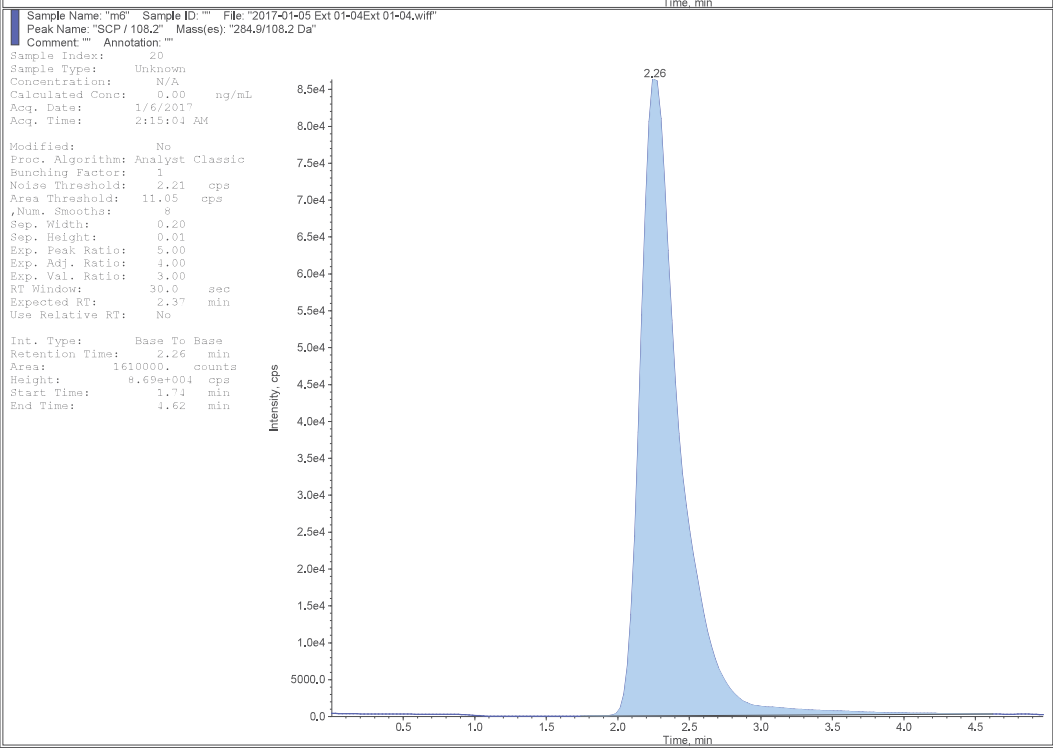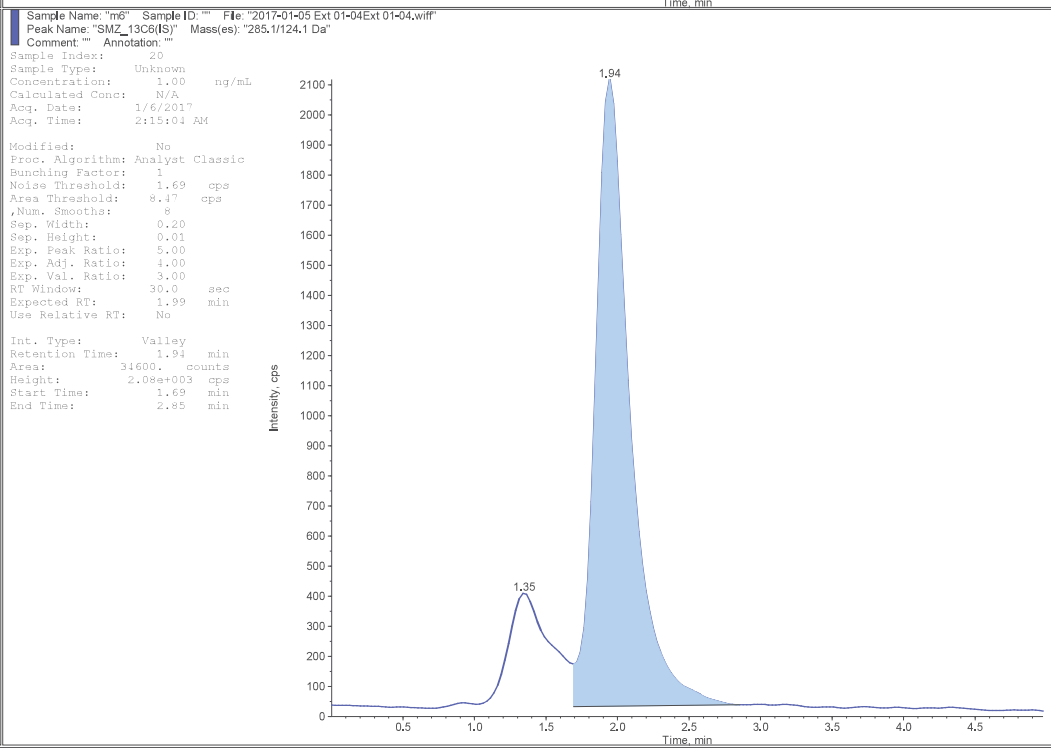

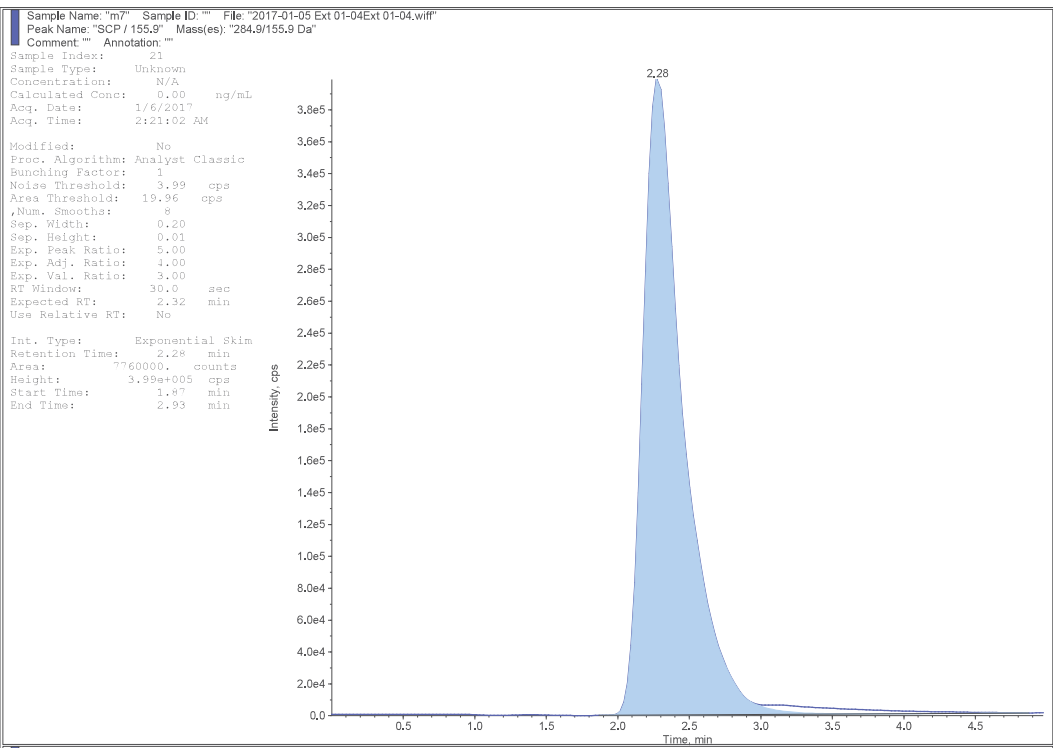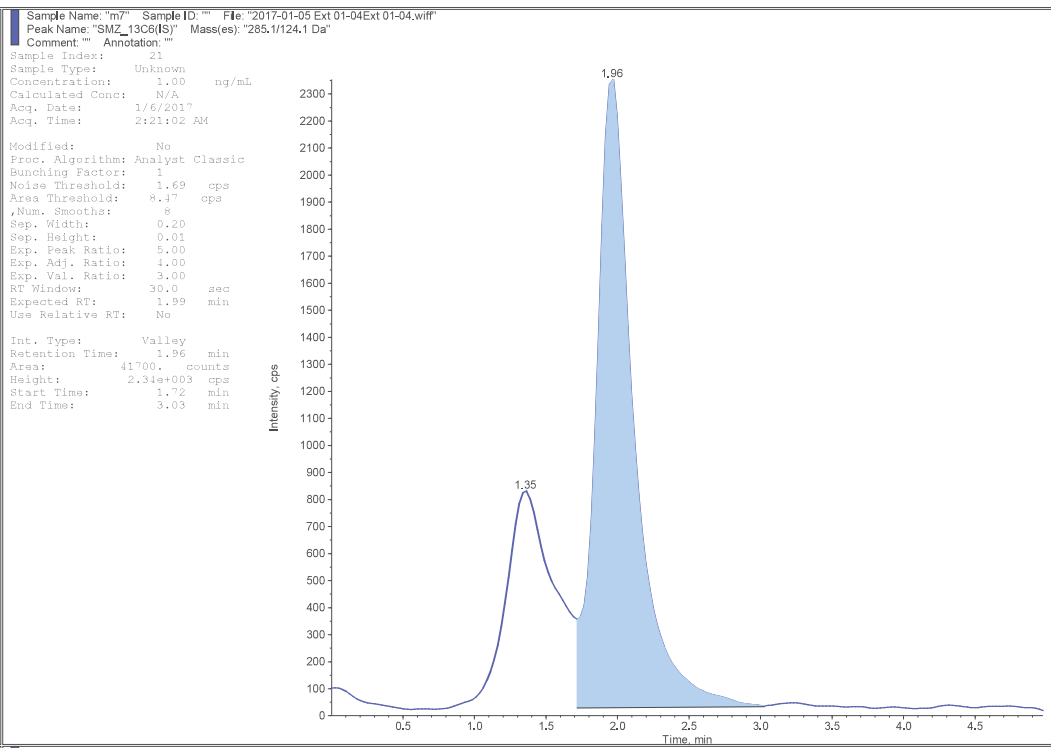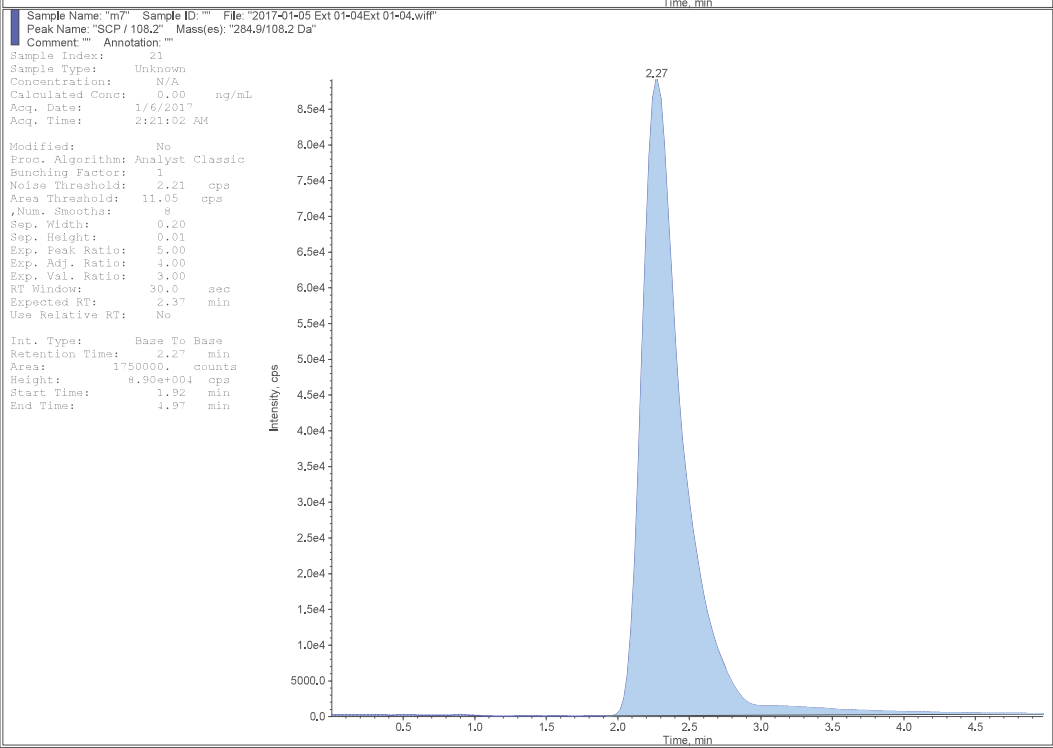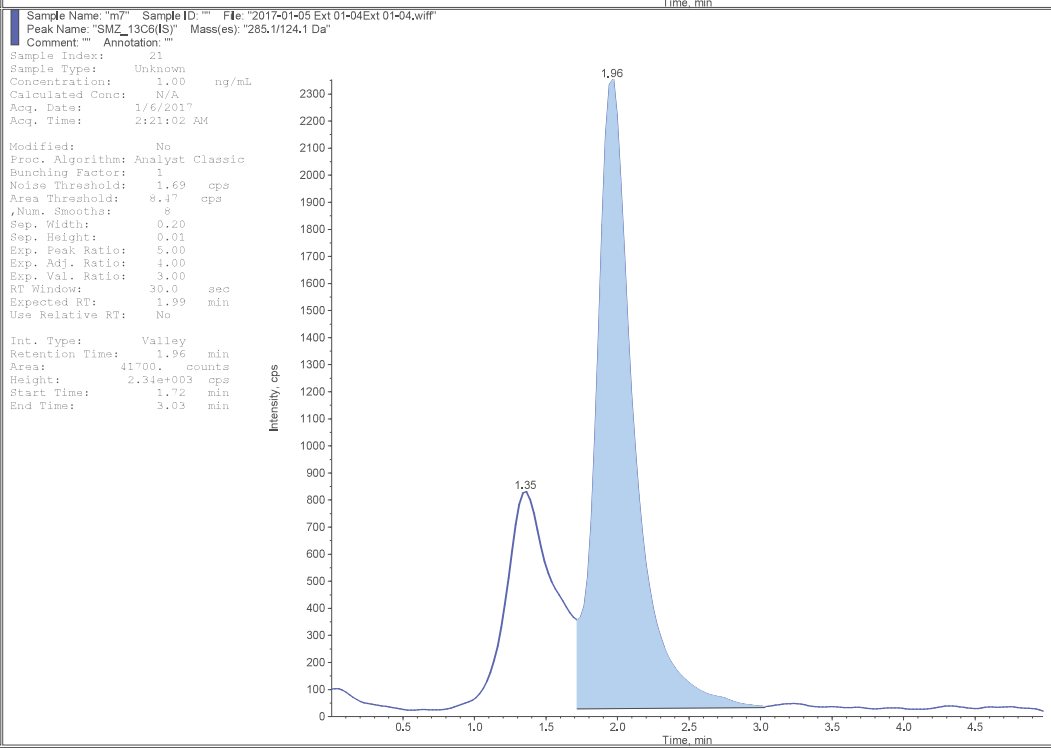

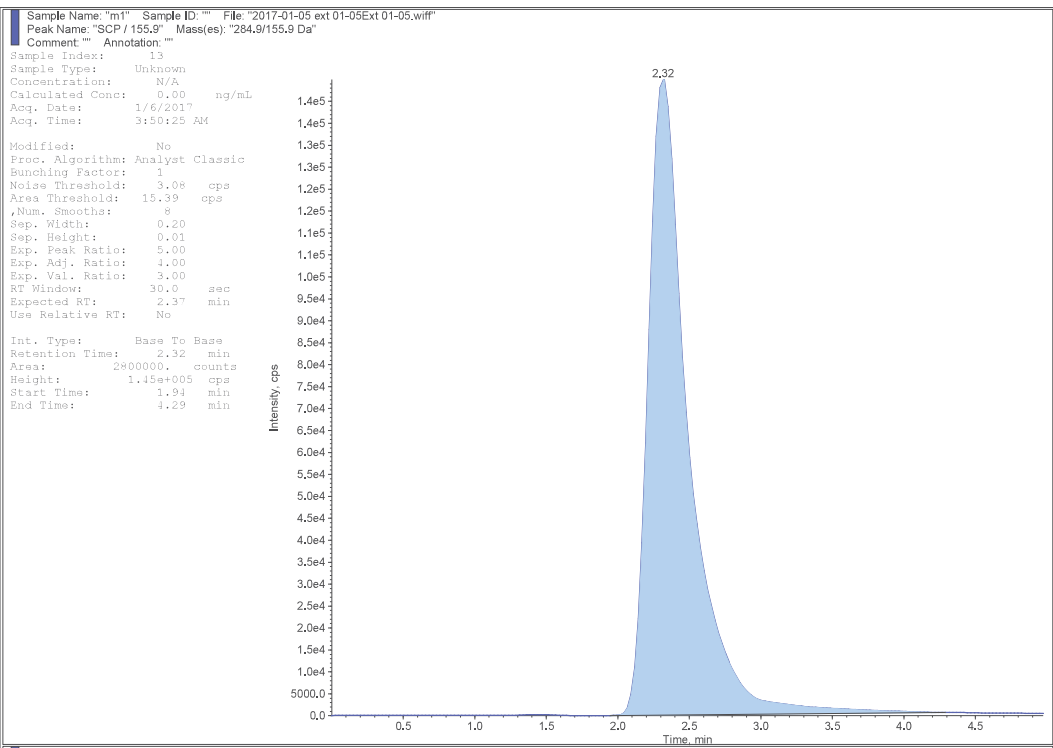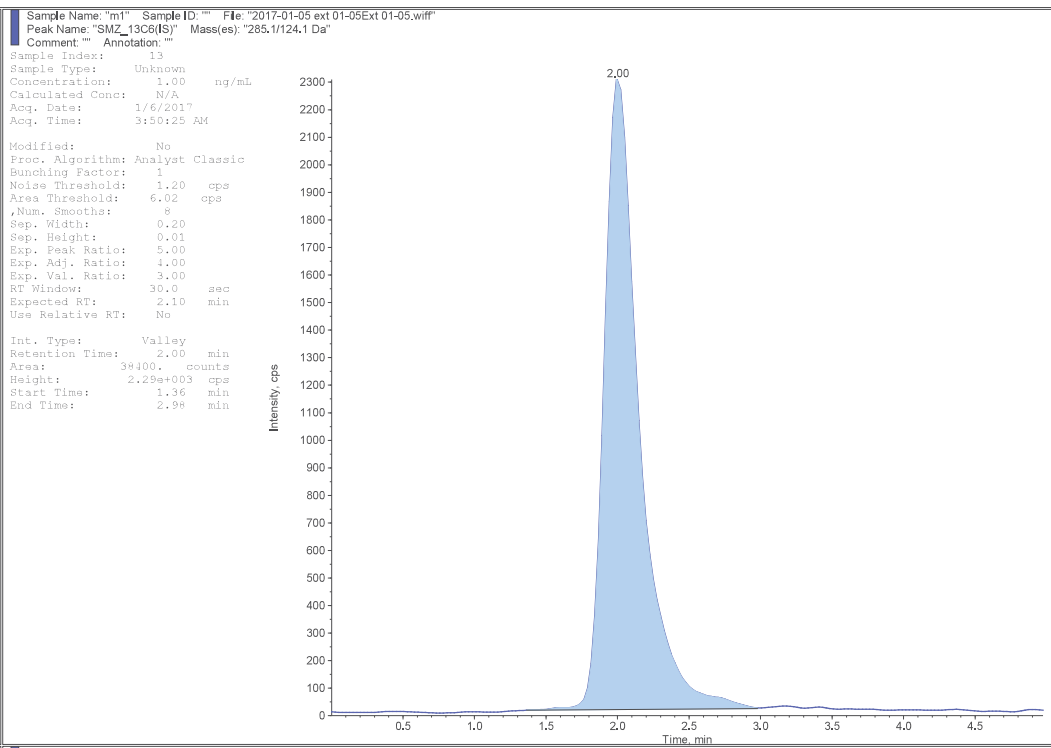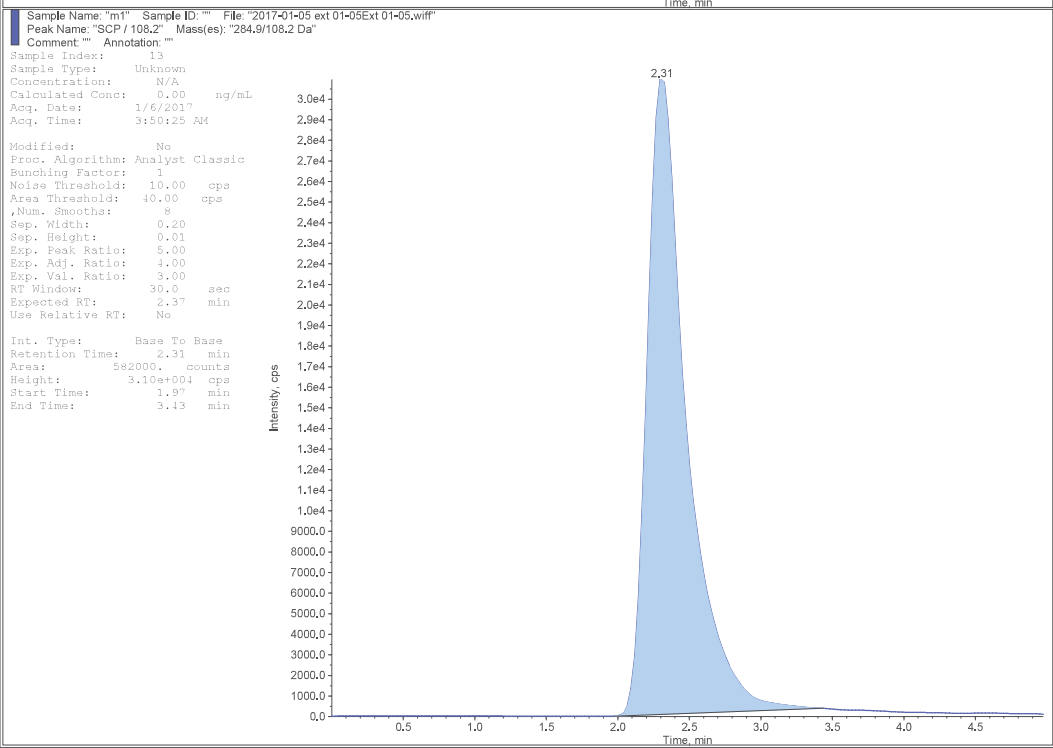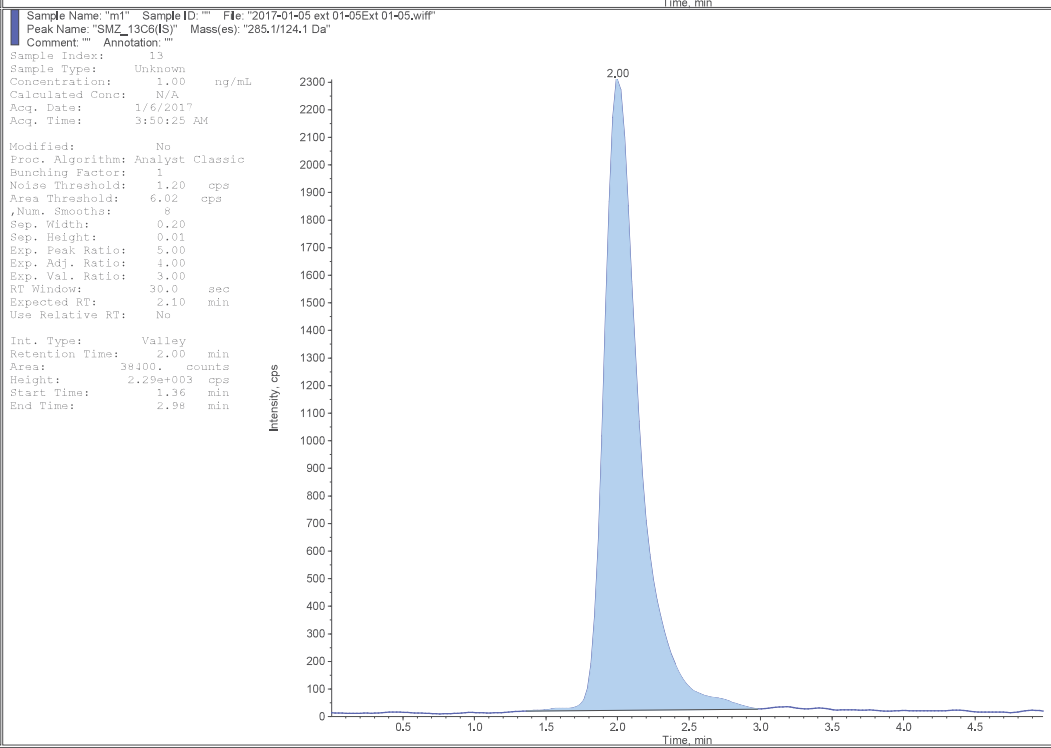

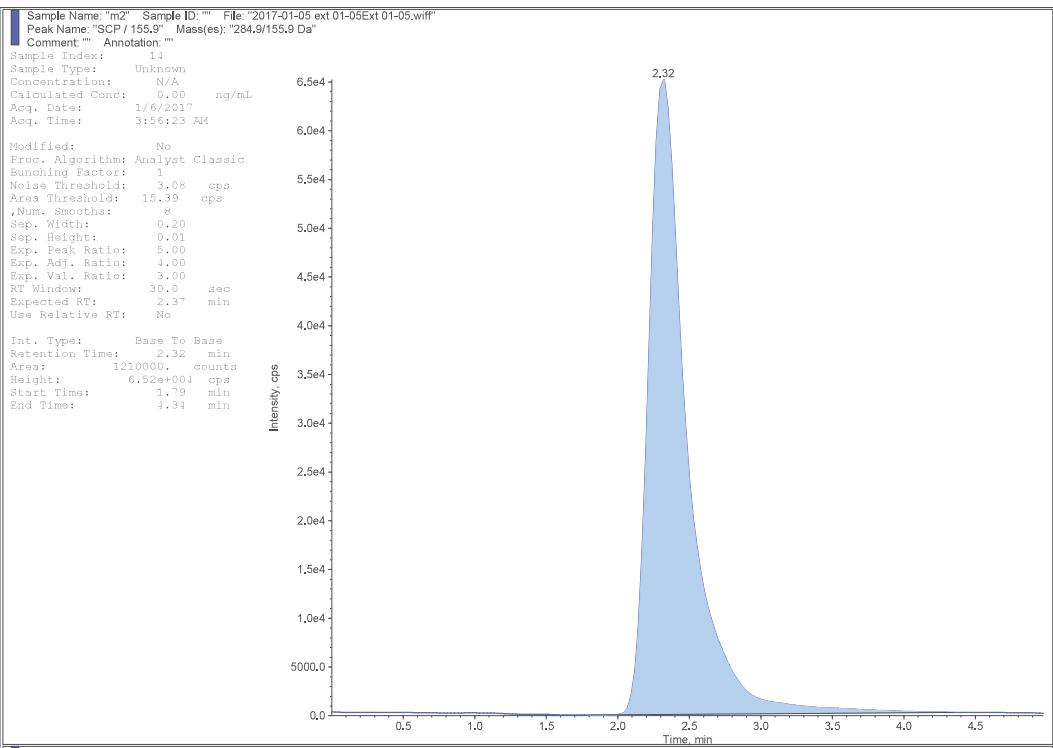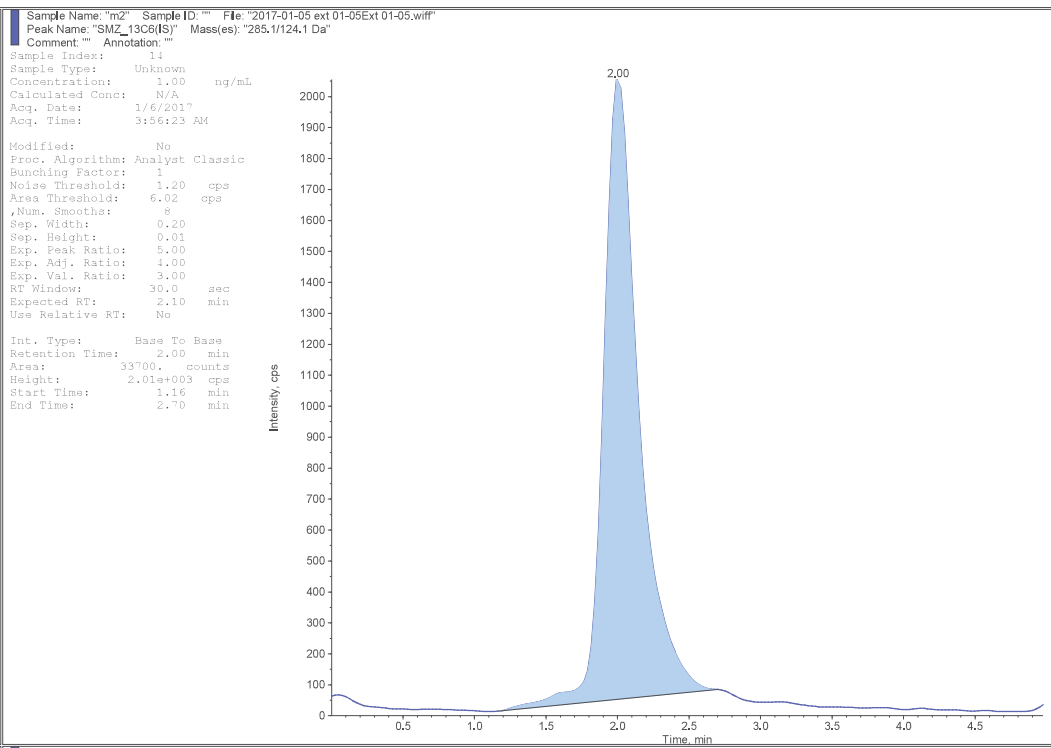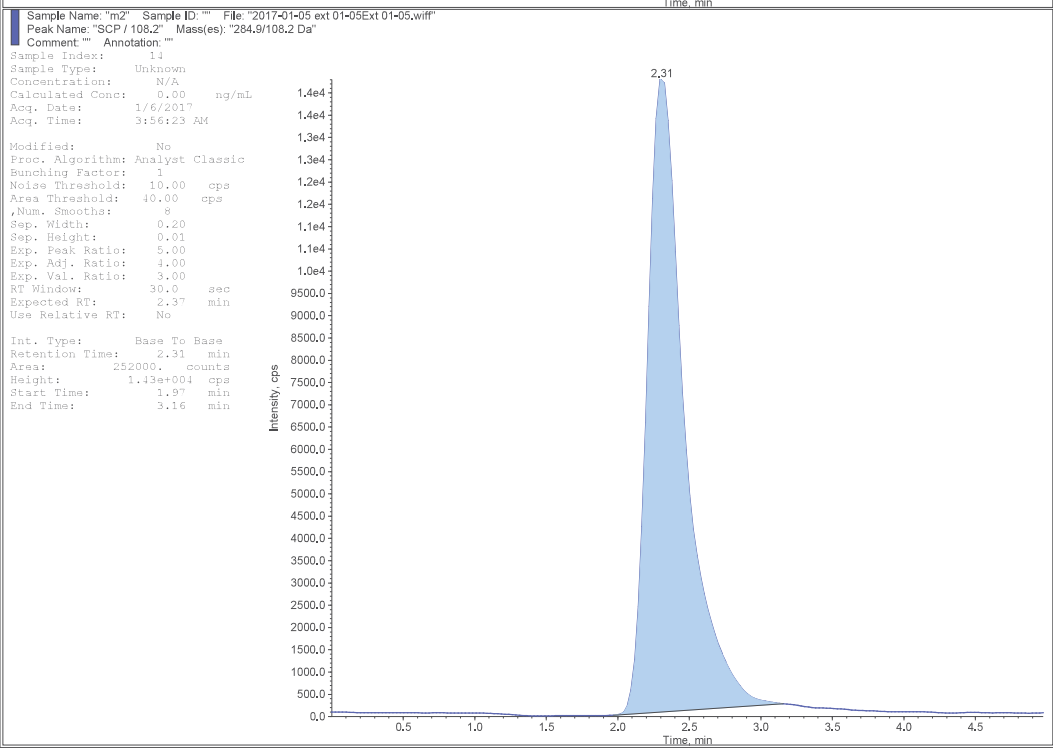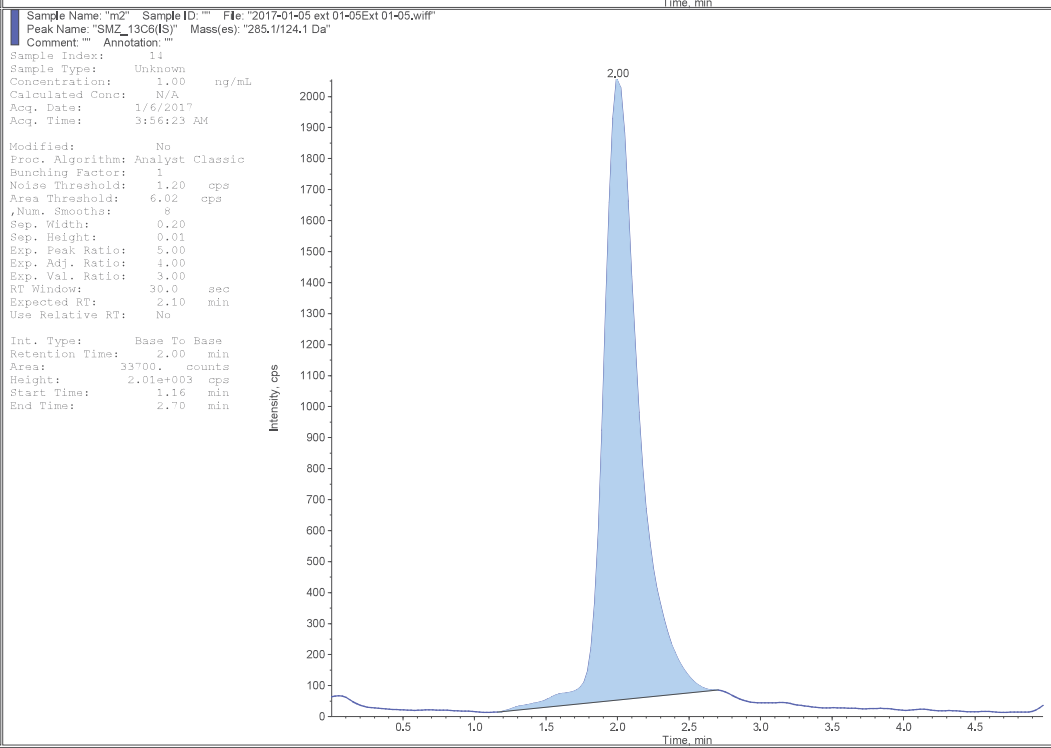

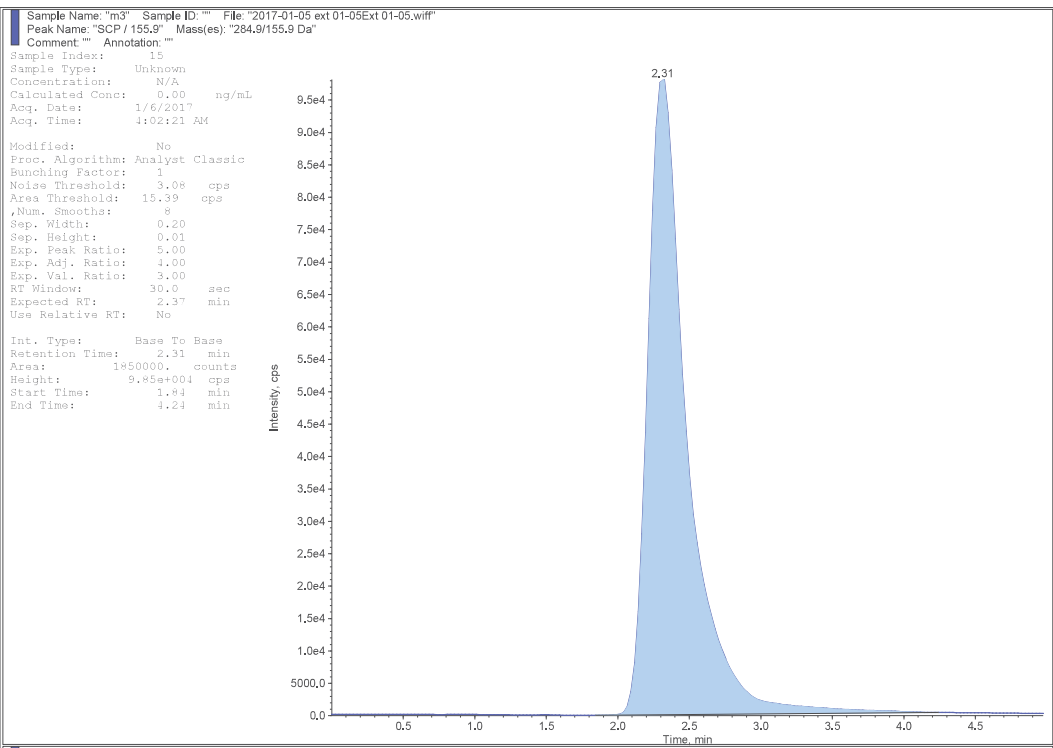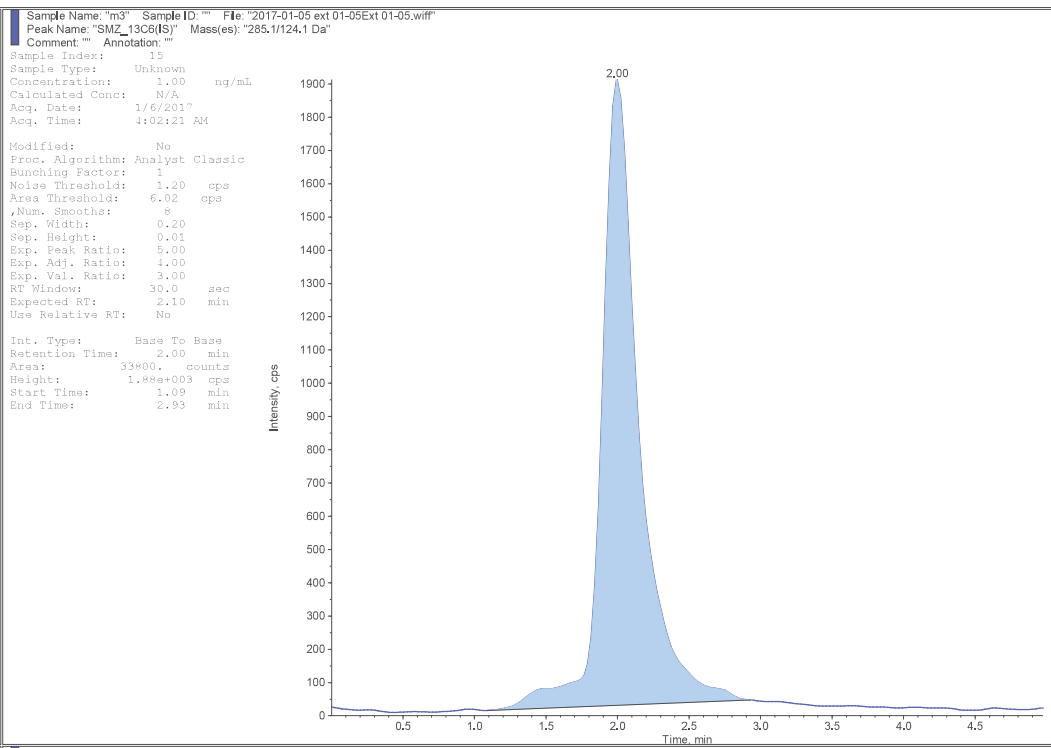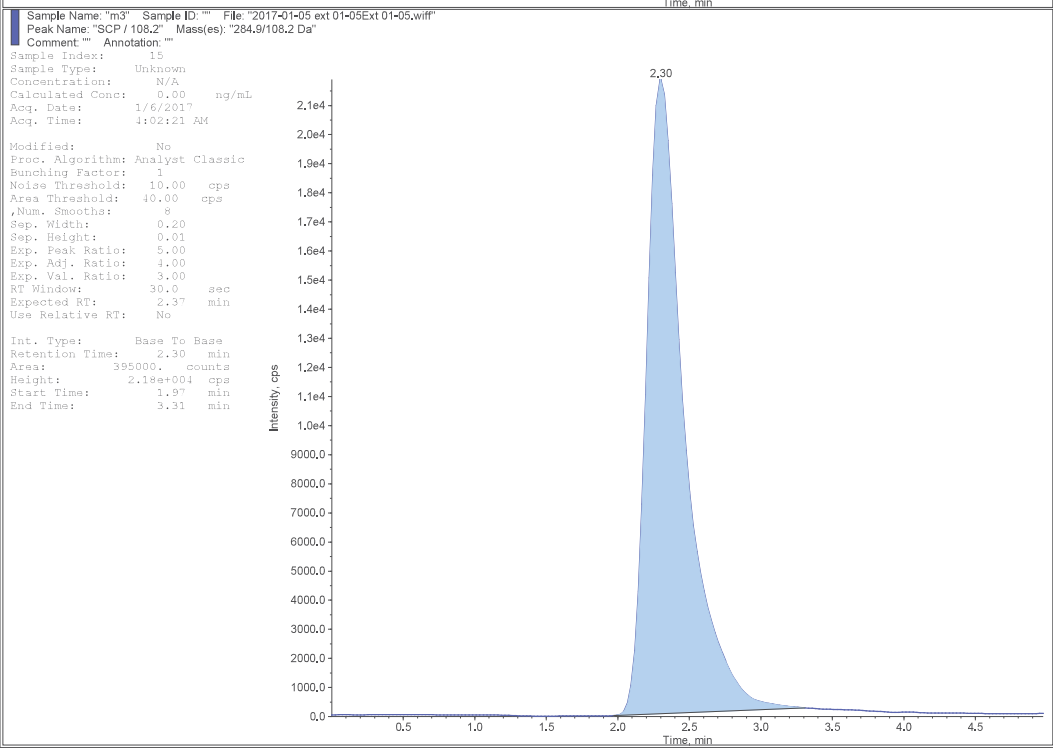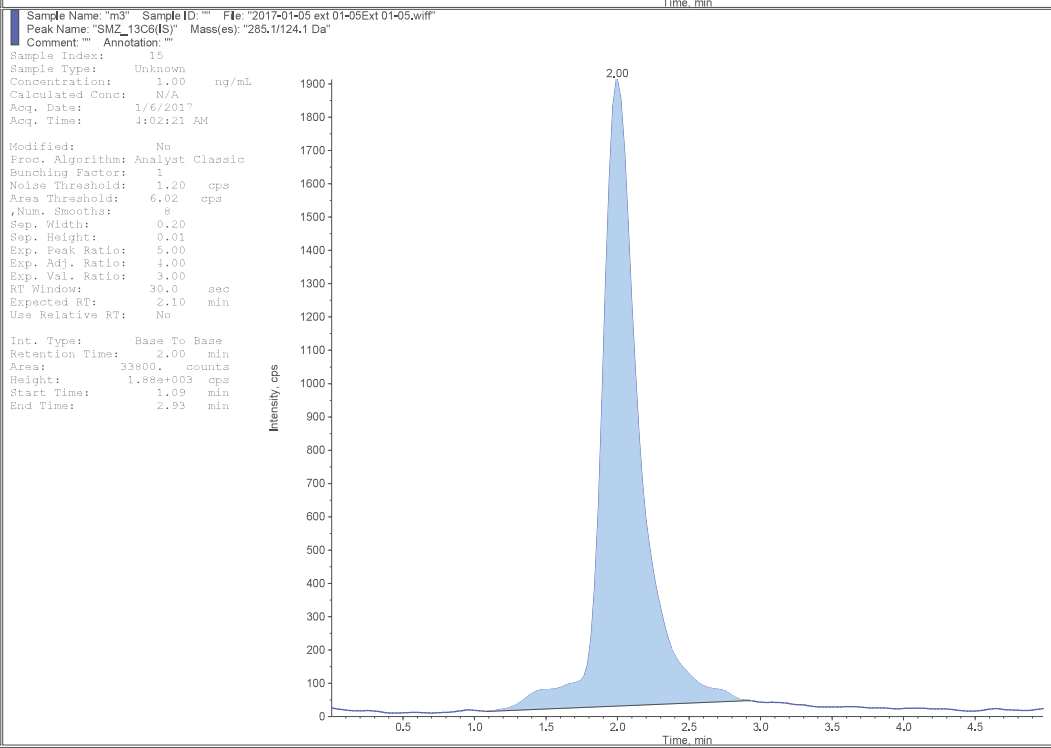

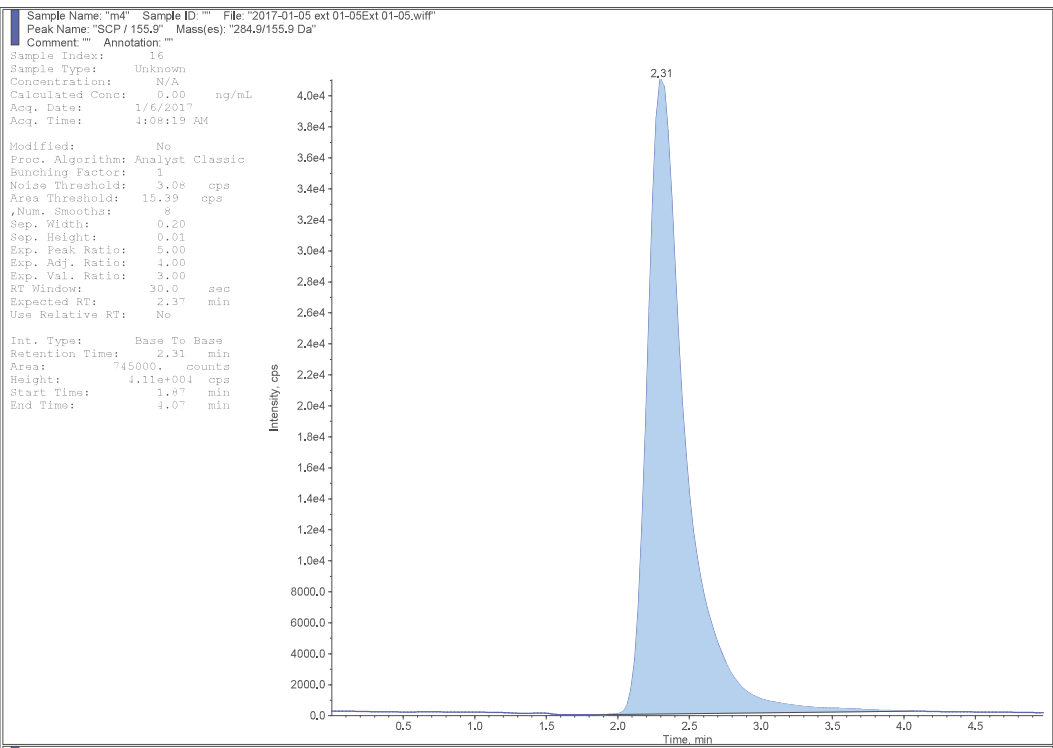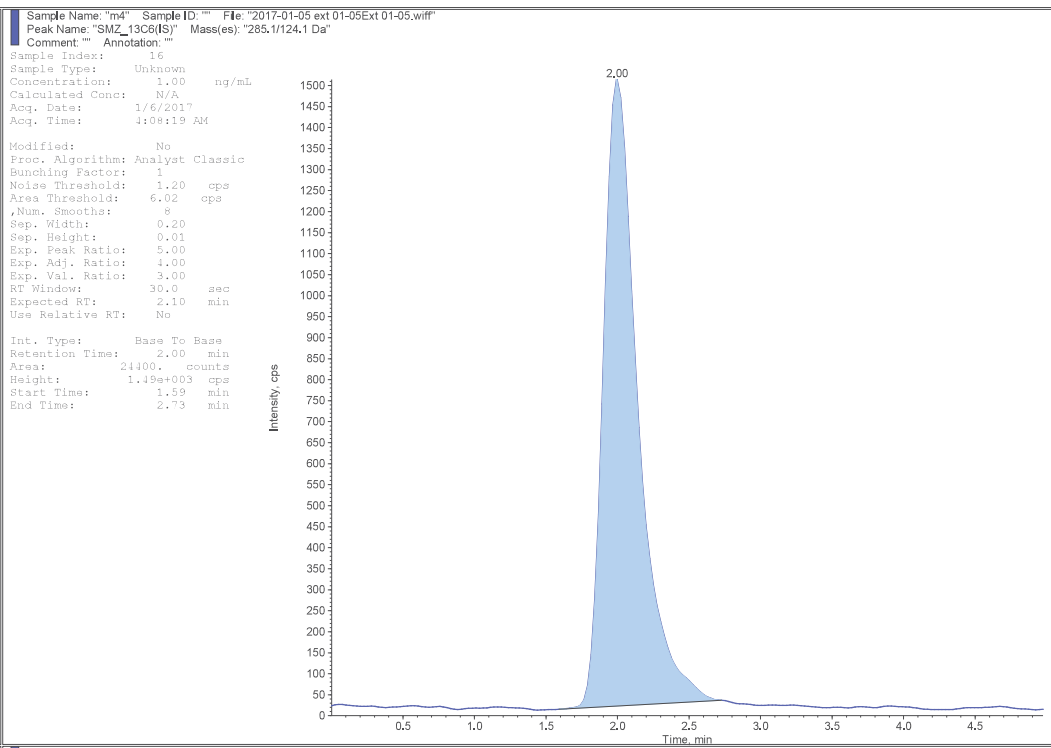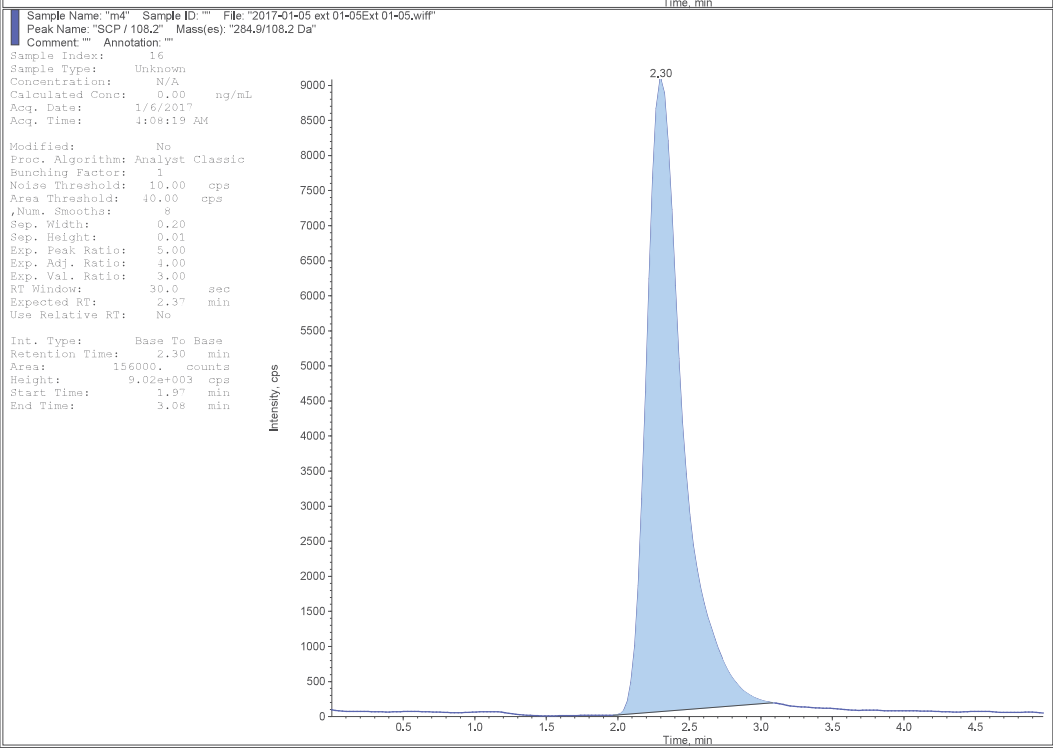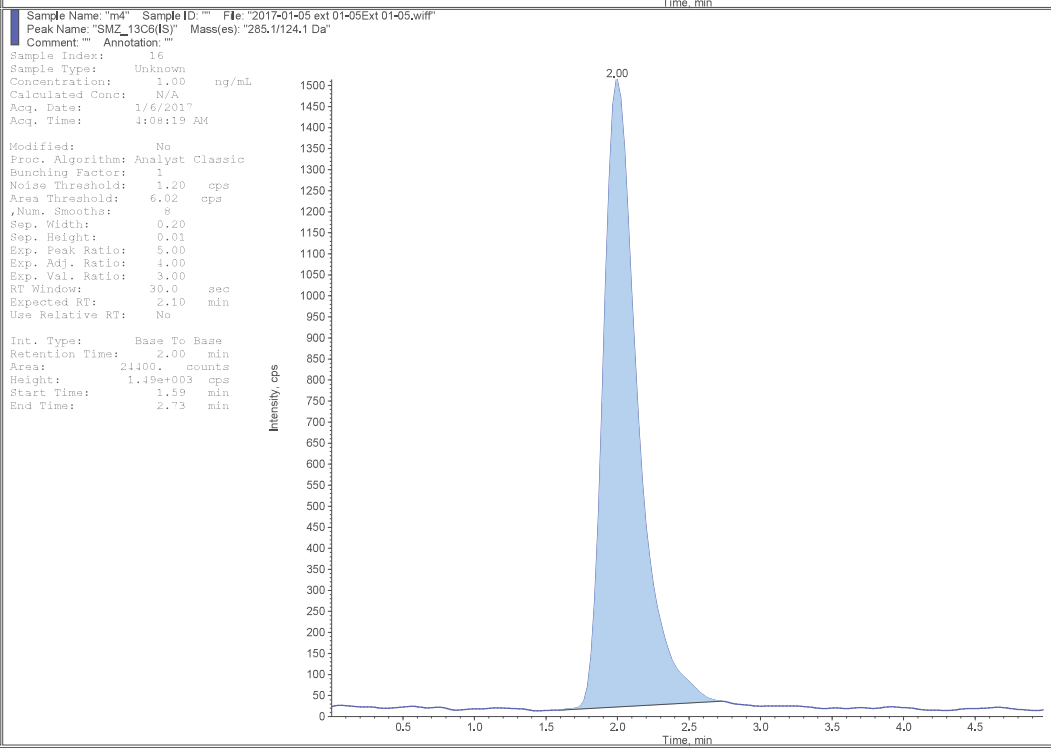

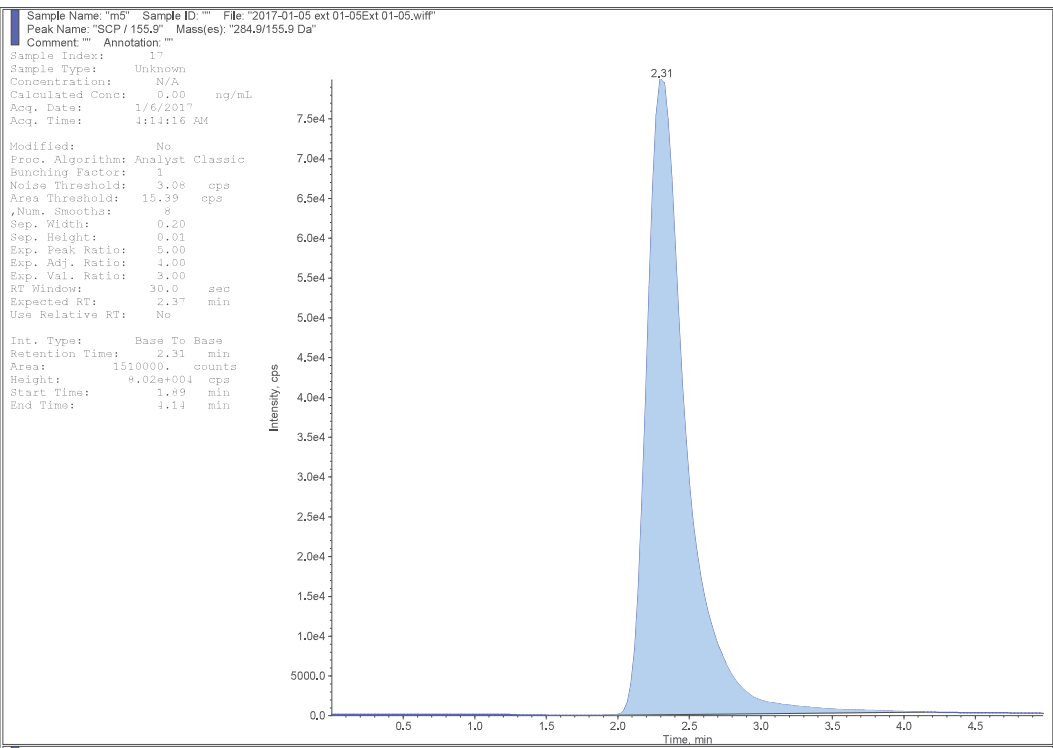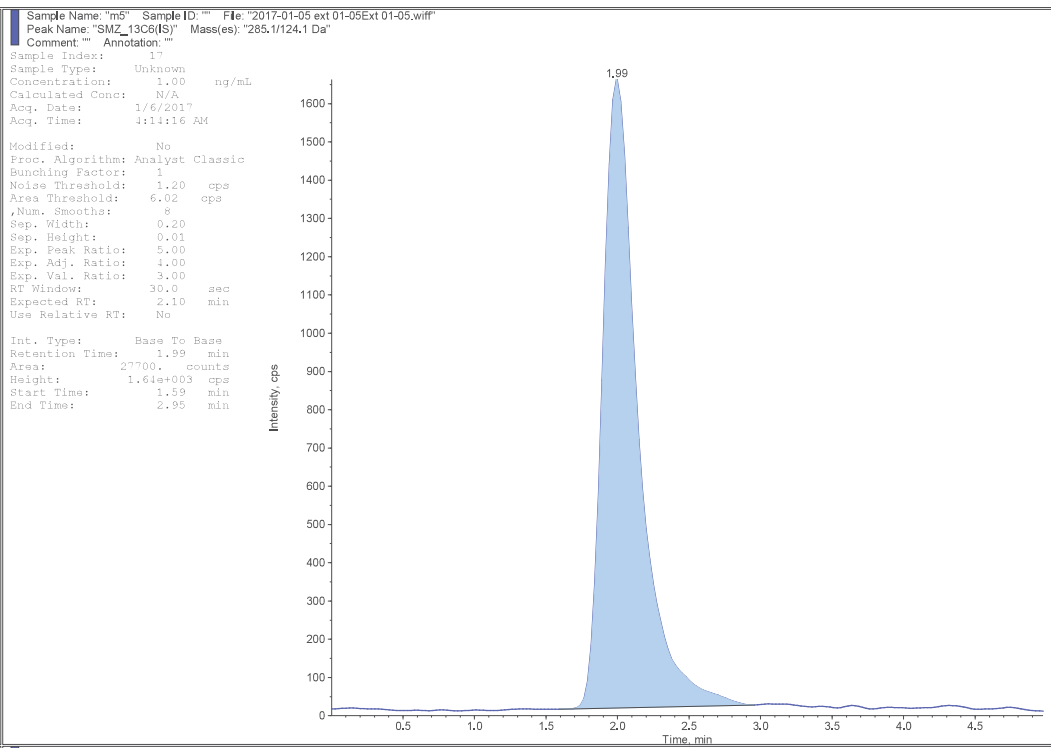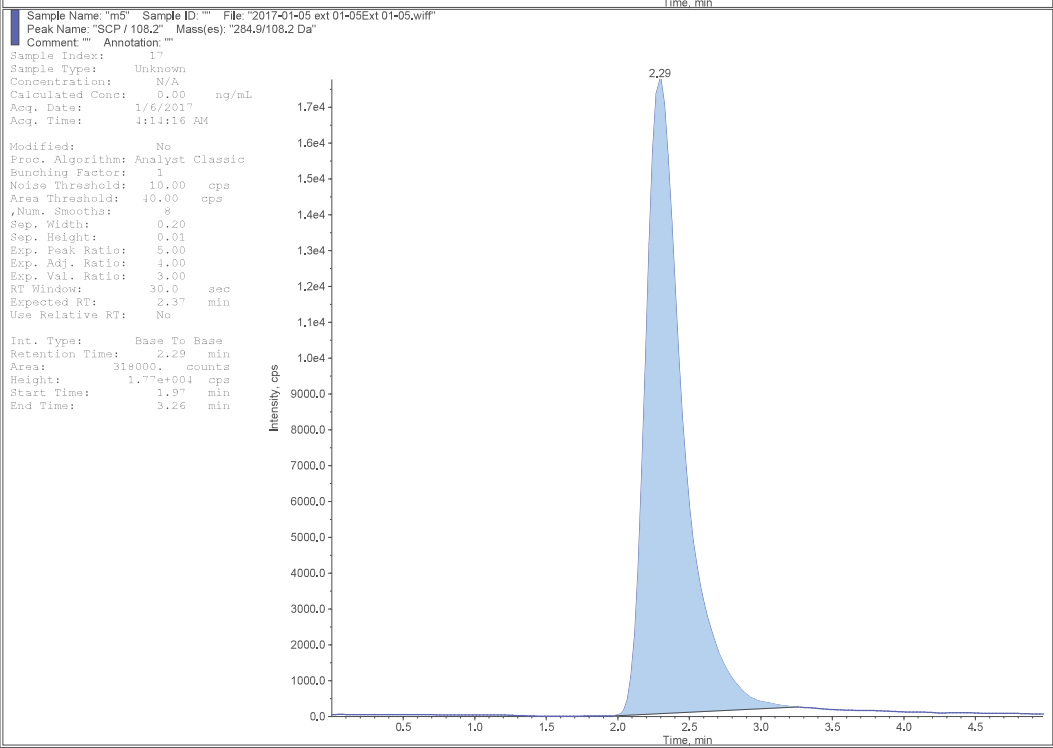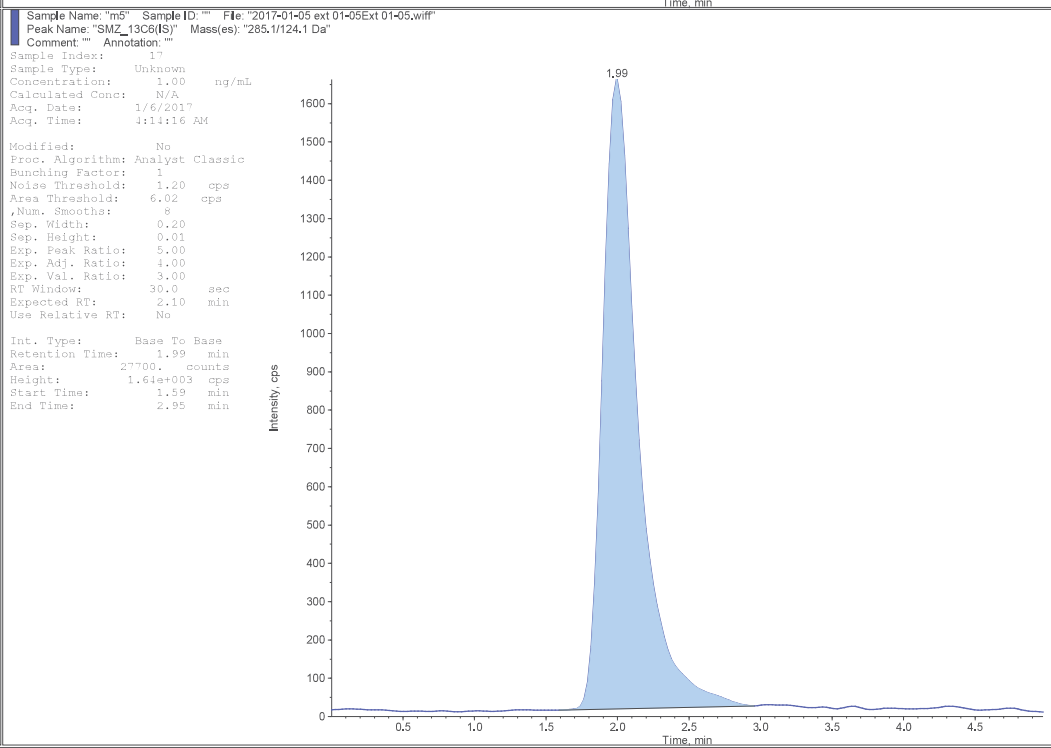

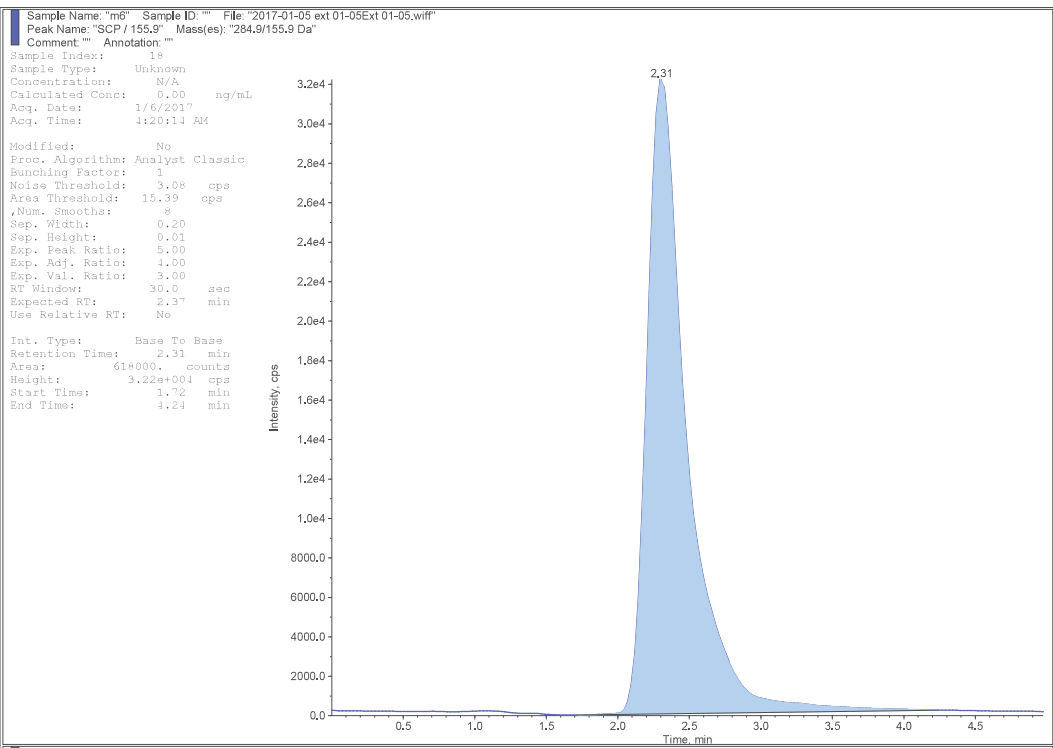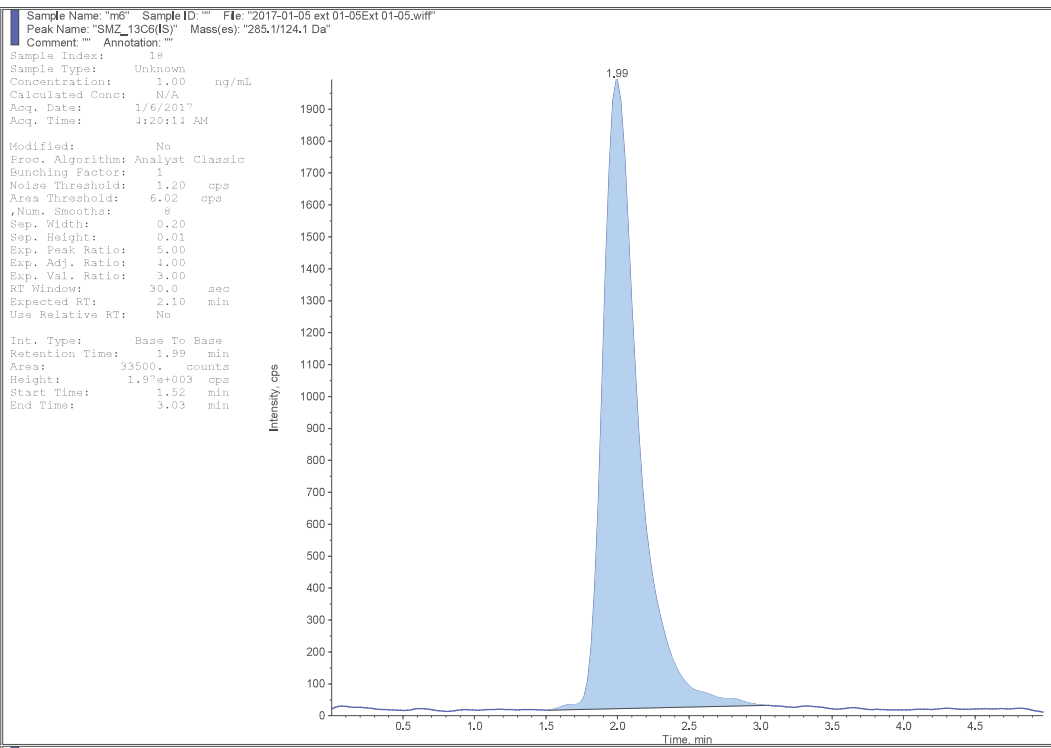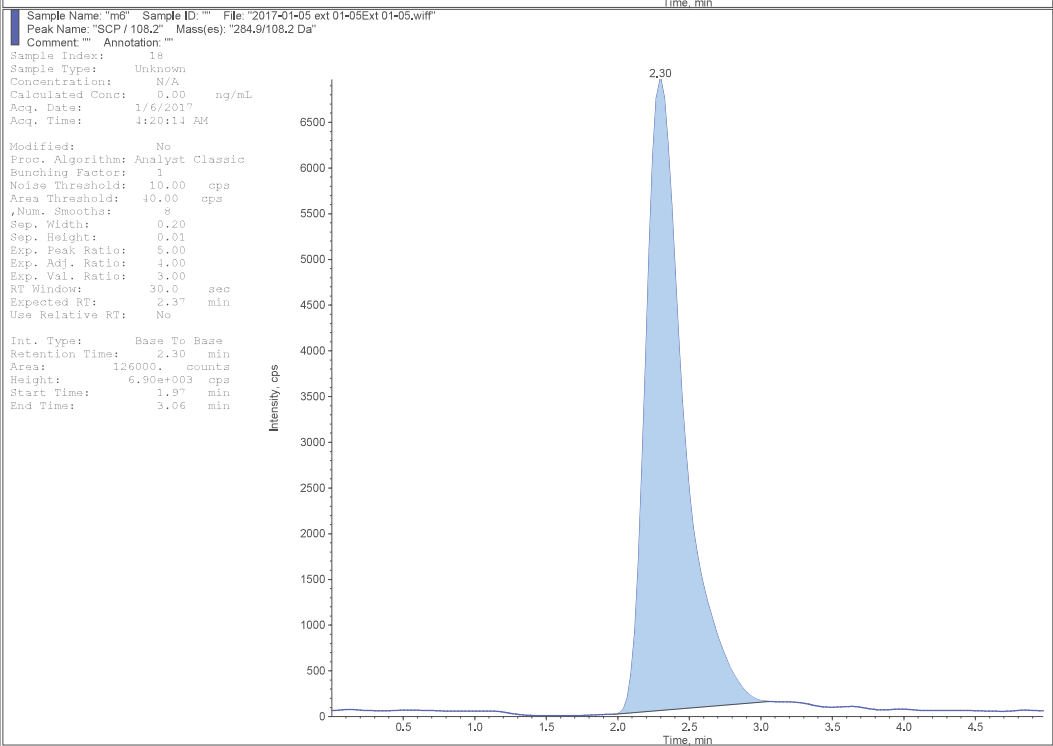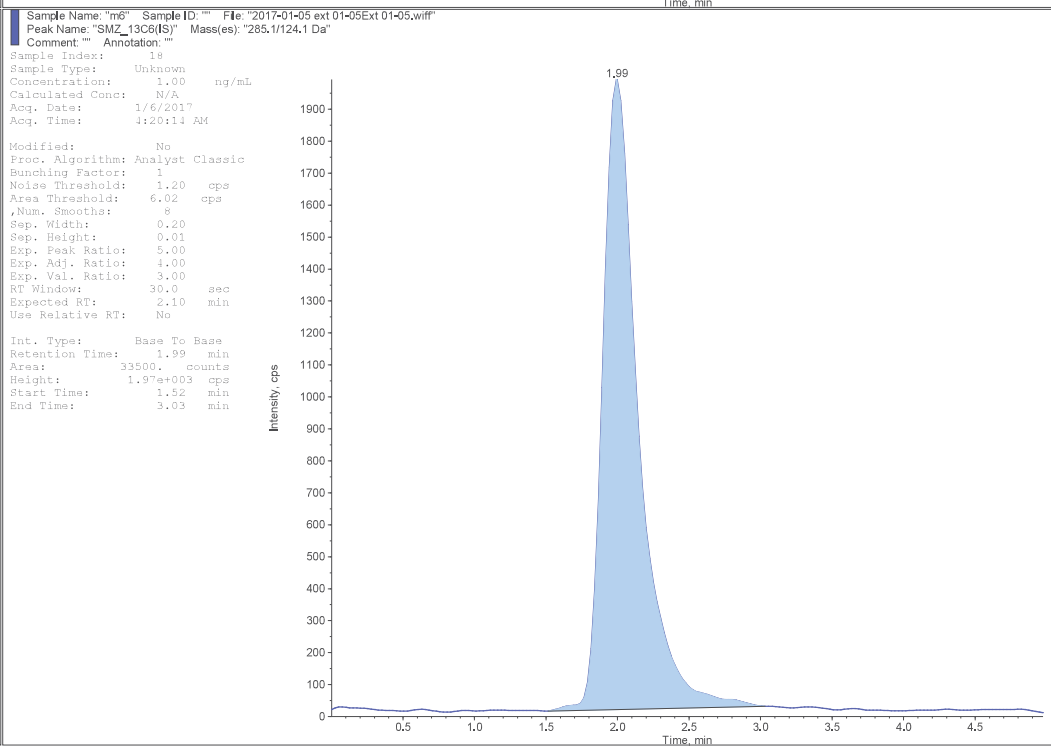

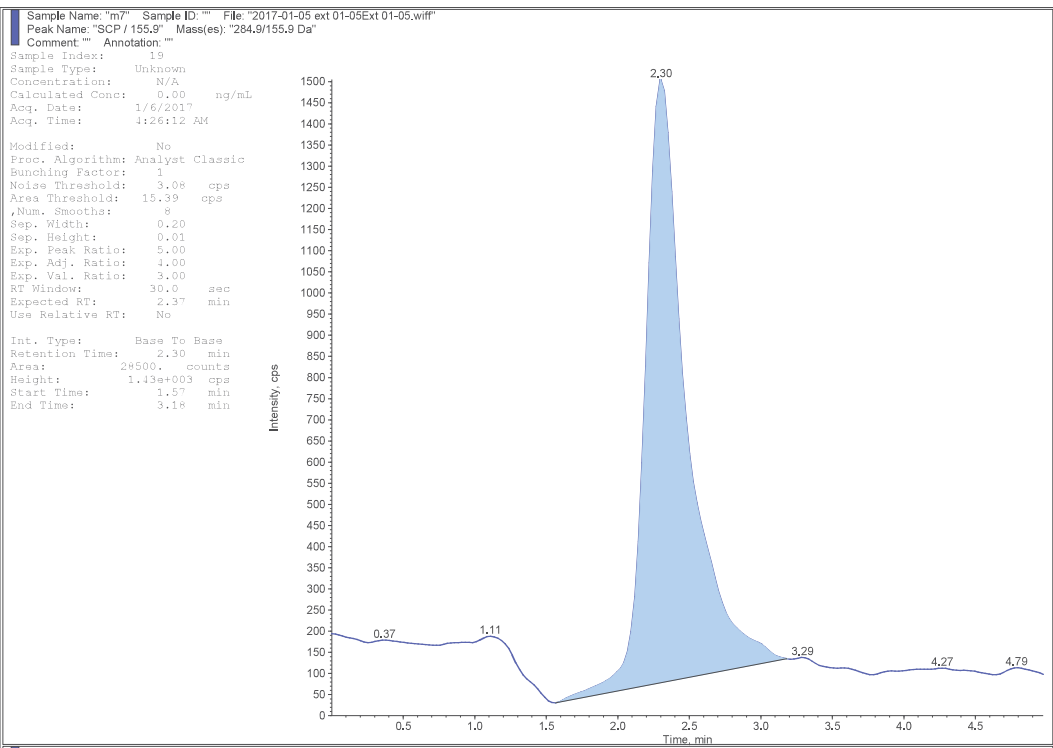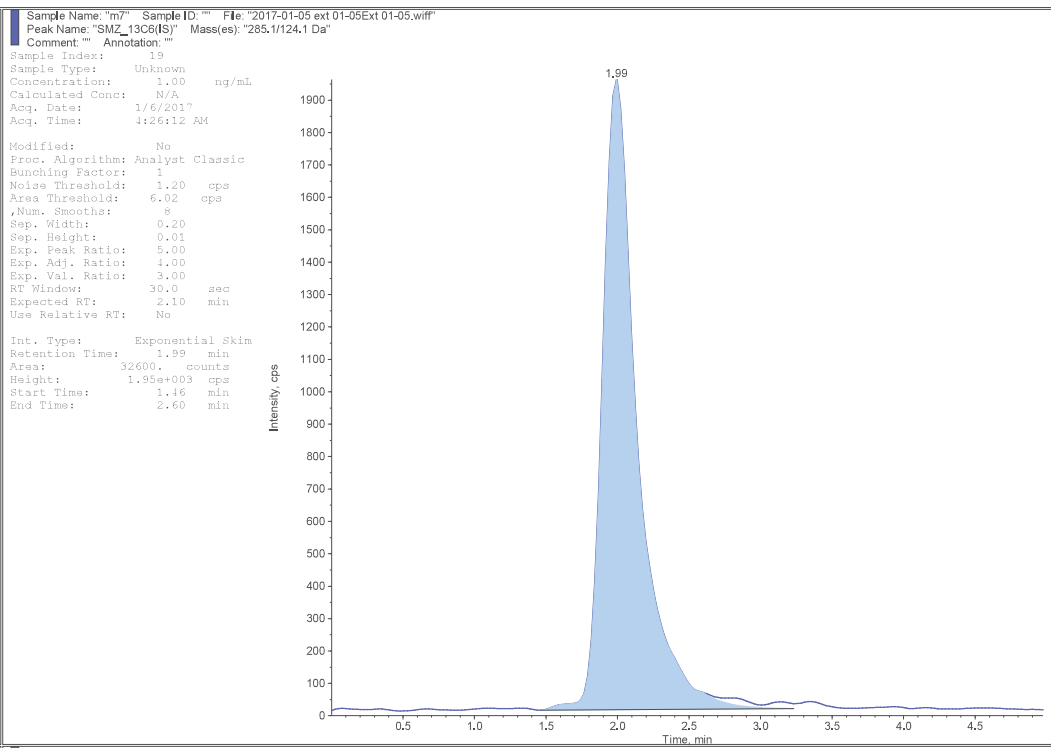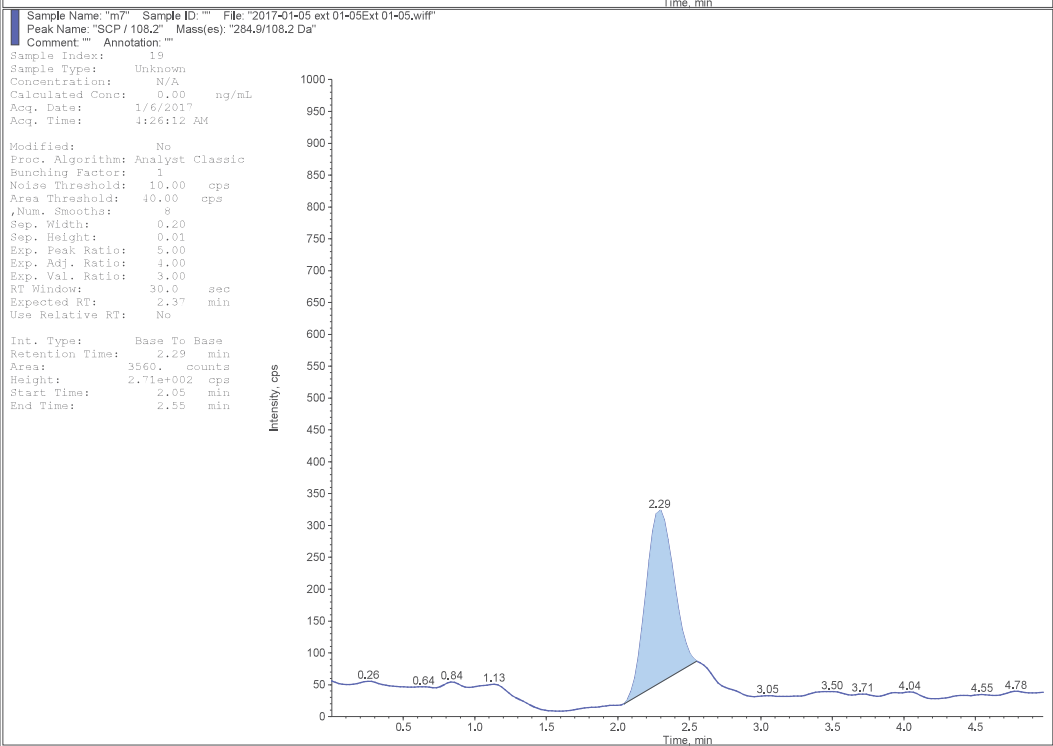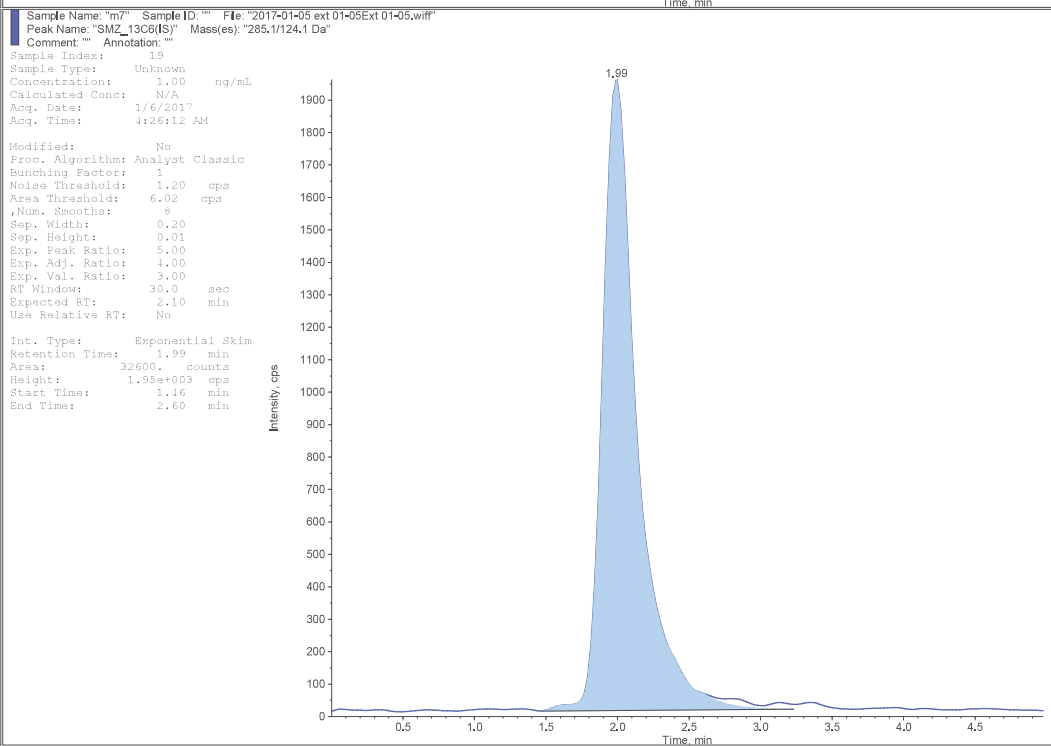

Supplement: S2 Fig — Samples from experimental animals treated with sulfachloropyridazine. Sulfamethazinephenyl-13C6 hemihydrate (SMZ-13C6) was used as Internal Standard for the fortification of the samples. (PDF) [file pone.0200206.s002.pdf]
