# Supplementary material for: Determination of sulfachloropyridazine residue levels in feathers from broiler chickens after oral administration using liquid chromatography coupled to tandem mass spectrometry
Source: PLoS One. 2018 Jul 5;13(7):e0200206. doi: 10.1371/journal.pone.0200206 (PMC6033452; doi:10.1371/journal.pone.0200206)
Supplement: S1 Dataset — Results for retention time, specificity, limit of detection, limit of quantification, linearity, recovery and precision parameters are detailed in this dataset. (PDF) [file pone.0200206.s003.pdf]

In-House Validation of Sulfachloropiridazine in Feathers

**Analytical Methodology**	Sulfachlorpyridazine in feathers
**Analyte**	Sulfaclorpiridazine
**Matrix/specie**	Feathers/Broiler chicken
**Equipment**	HPLC
**Detector**	MS/MS
**Column**	Symetry C8 3.5um 2.1 x 100 mm Waters
**Solvents**	Mobil phase A (0.1% formic acid in methanol (pH 2.9 ± 0.3)). Mobil phase B (0.1% formic acid diluted in water (pH 2.7 ± 0.2)).

Parameters	Validation
**1. Retention Time (RT)**	(SCP) 2,5 min / CV: 1,68%<br>(SMZ 13C6) 2,1 min / CV: 0,73%
**2. Specificity**	There are no interferers in the RT of the analyte
**3. Limit of Detection (LOD)**	10 ng/gr.
**4. Limit of Quantification (LOQ)**	14,6 ng/gr.
**5. Linearity of calibration curve**	R^2 Curve 1= 0,9836; R^2 Curve 2= 0,9957; R^2 Curve 3= 0,9884<br>Slopes CV: 0,62%
**6. Recovery (Average)**	10 ng/gr: 98,38%; 40 ng/gr: 100,60%; 100 ng/gr: 99,92%
**7. Precision :**	
**7.1. Repeatability**	10 ng/gr: 23,3%; 40 ng/gr: 5,3%; 100 ng/gr: 0,7%
**7.2. Intra-laboratory reproducibility**	10 ng/gr: 26,2%; 40 ng/gr: 10,6%; 100 ng/gr: 1,4%

Retention Time
Sulfachloropyridazine

To determine the Retention Time of an analyte, 6 pure drugs are injected. There should not be a difference greater than 5% between the injections.

Analyte	Retention Time (min)								
	1	2	3	4	5	6	Promedio	Desv. Est.	CV
Sulfachloropyridazine (284.9/155.9)	2.54	2.54	2.54	2.47	2.46	2.46	2.502	0.0422	1.68%
Sulfametazina 13C6 (285.1/124.1)	2.1	2.09	2.1	2.13	2.12	2.12	2.110	0.0155	0.73%

Limit of Detection and Limit of Quantification
Sulfachloropyridazine

Sample	Concentration (ng/gr)	Area Ratio	Quantified Concentration (ng/gr)
**1**	10	2.55	5.08
**2**	10	4.28	9.59
**3**	10	3.41	7.32
**4**	10	4.57	10.35
**5**	10	3.59	7.79
**6**	10	5.39	12.49
**7**	10	5.14	11.84
**8**	10	5.09	11.71
**9**	10	4.85	11.08
**10**	10	6.15	14.48
**11**	10	5.37	12.44
**12**	10	5.32	12.31
**13**	10	4.93	11.29
**14**	10	4.80	10.95
**15**	10	4.70	10.69
**16**	10	5.18	11.95
**17**	10	6.17	14.53
**18**	10	6.27	14.79
**19**	10	6.34	14.98
**20**	10	5.43	12.60
**Promedio**		4.977	11.414
**Desv. Estándar**		0.976	2.790
**CV (%)**		19.62	24.44

**LC = LD + 1,64*LD**	
**LD (ppb)**	10
**LC (ppb)**	14.6

Sulfachlorpyridazine		
**Sample**	**Concentration (ng/gr)**	**Area Ratio**
F1	10	5.09
F2	20	8.05
F3	40	16.80
F4	80	26.50
F5	100	42.30
	**Slope**	0.3828
	**Intercept**	0.6072

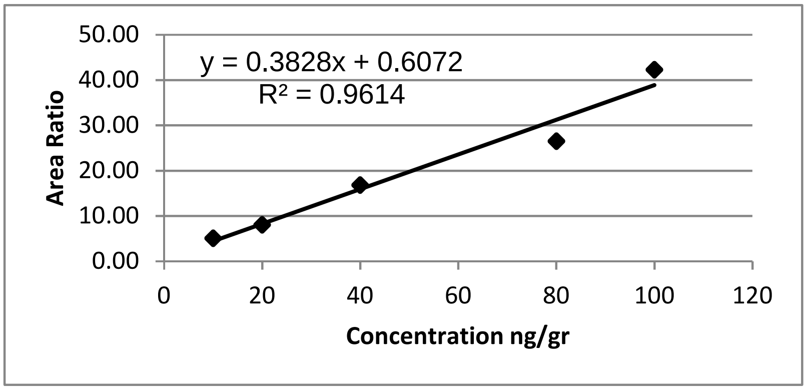

Linearity of the Claibration Curve

Sulfachloropyridazine Feather

Calibration Curve a		
**Sample**	**Concentration (ng/g)**	**Area Ratio**
F1a	10	4.37
F2a	20	6.36
F3a	40	18.6
F4a	80	32.2
F5a	100	46.8

**Solpe**	0.4591
**Intercept**	-1.2873

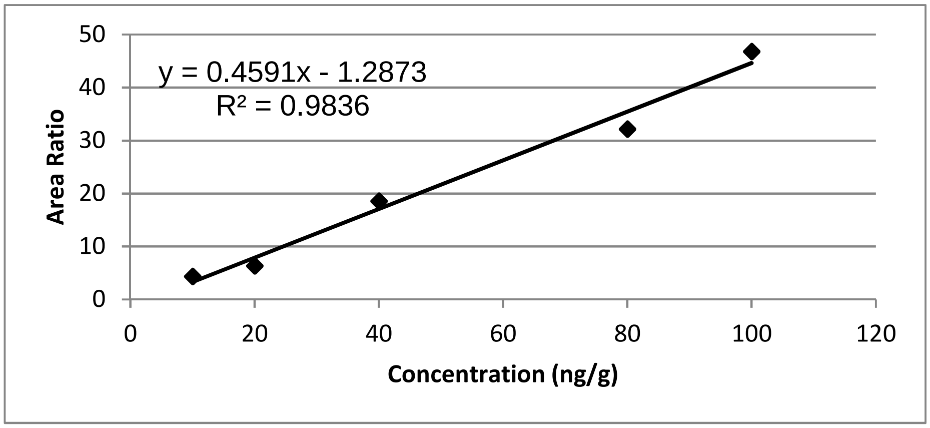

Calibration Curve b		
**Sample**	**Concentration (ng/g)**	**Area Ratio**
F1b	10	3.45
F2b	20	5.26
F3b	40	8.13
F4b	80	15.6
F5b	100	17.8

**Slope**	0.1635
**Intercept**	1.8738

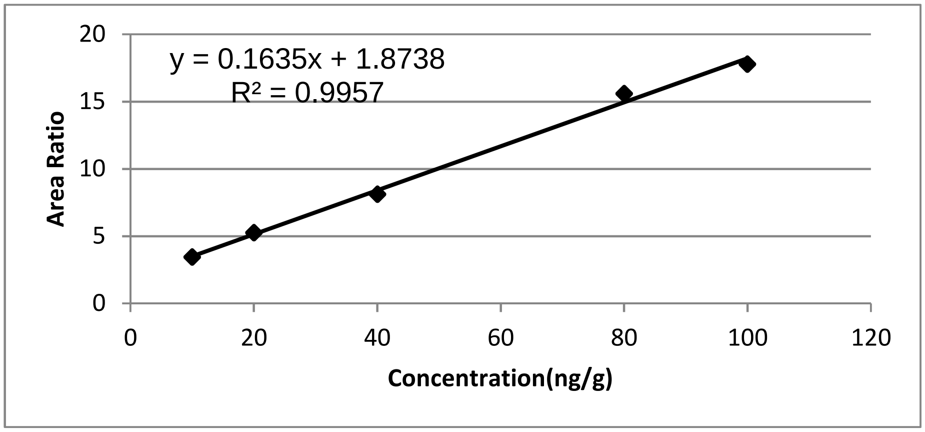

Linearity of the Claibration Curve

Sulfachloropyridazine Feather

Calibration Curve c		
**Sample**	**Concentration (ng/g)**	**Area Ratio**
F1c	10	2.04
F2c	20	3.09
F3c	40	6.06
F4c	80	11.5
F5c	100	12.6

**Slope**	0.1234
**Intercept**	0.8905

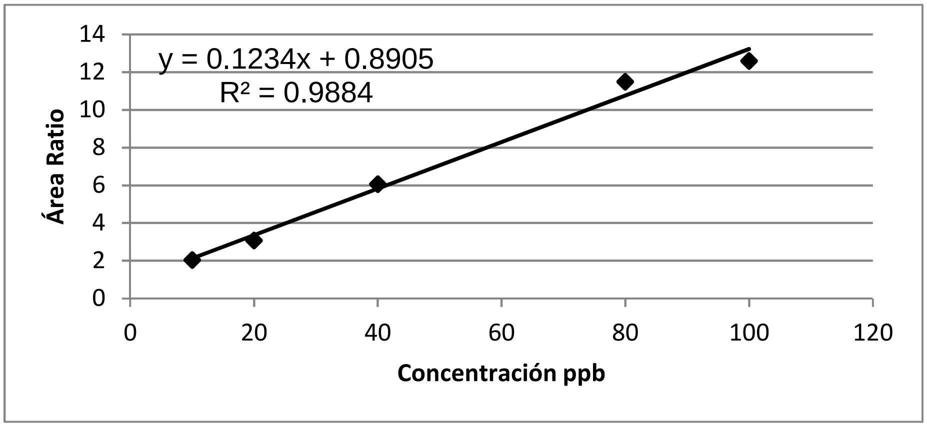

Curve	R2	Average	SD	CV (%)
Curve a	0.9836	0.99	0.01	0.62
Curve b	0.9957			
Curve c	0.9884			

Recovery
Sulfachloropyridazine Feather

Sulfachloropyridazine			
Sample	Fortification Concentration (ng/g)	Quantified Concentration (ng/g)	% of Recovery
1	10	9.8	97.70%
2	10	7.3	73.00%
3	10	16.6	166.00%
4	10	8.0	80.00%
5	10	8.0	80.30%
6	10	9.3	93.30%
Average			98.38%
SD			0.34
CV			35%

Sulfachloropyridazine			
Sample	Fortification Concentration (ng/g)	Quantified Concentration (ng/g)	% of Recovery
1	40	40.3	100.85%
2	40	44.1	110.15%
3	40	30.1	75.25%
4	40	43.0	107.48%
5	40	43.0	107.40%
6	40	41.0	102.50%
Average			100.60%
SD			0.13
CV			13%

Sulfachloropyridazine			
Sample	Fortification Concentration (ng/g)	Quantified Concentration (ng/g)	% of Recovery
1	100	99.9	99.90%
2	100	98.6	98.60%
3	100	103.3	103.30%
4	100	99.0	99.00%
5	100	99.0	99.00%
6	100	99.7	99.70%
Average			99.92%
SD			0.02
CV			2%

Precision
Sulfachloropyridazine Feather

Repeatability										
Fortification concentration	Quantified Concentration						Average	SD	CV	CV %
	a	b	c	d	e	f				
10	7.8	7.7	4.5	6.7	5.3	8.5	6.7	1.57	0.23	23.33
40	43.3	43.4	48.3	44.9	47.1	42.3	44.9	2.36	0.05	5.25
100	98.4	98.4	97.2	98.4	97.6	99.2	98.2	0.70	0.01	0.72

Acceptance Criteria: Repetitivity is accepted when the CV (%) of each measured concentration is in the range of values corresponding to half or equal to the CV (%) of the intralaboratory reproducibility.

Concentration	CV %		Criteria
	Repeatability	Intralaboratory reproducibility	
10	23.3	26.2	**accepted**
40	5.3	10.6	**accepted**
100	0.7	1.4	**accepted**

Intralaboratory Reproducibility										
Fortification concentration	Quantified Concentration						Average	SD	CV	CV %
	a	b	c	d	e	f				
10	11.1	11.4	8.8	6.6	14.8	12.3	10.8	2.84	0.26	26.23
40	38.4	37.9	41.8	45.0	33.2	36.6	38.8	4.12	0.11	10.61
100	100.5	100.7	99.4	98.3	102.3	101.1	100.4	1.39	0.01	1.38

Acceptance Criteria: Reproducibility is accepted when the CV (%) of each measured concentration is lower than indicated by the following table:

Mass fraction	CV of Intralaboratory Reproducibility (%)
1 (ng/g)	35
10 (ng/g)	35
100 (ng/g)	23
1000 (ng/g)	16

Intralaboratory Reproducibility is accepted, CV% is lower than that indicated for each mass fraction.
