# Supplementary material for: Determination of sulfachloropyridazine residue levels in feathers from broiler chickens after oral administration using liquid chromatography coupled to tandem mass spectrometry
Source: PLoS One. 2018 Jul 5;13(7):e0200206. doi: 10.1371/journal.pone.0200206 (PMC6033452; doi:10.1371/journal.pone.0200206)

Depletion of Sulfachloropyridazine in feathers

Samplng point (days)	Post-treatment day	Sample	Lifetime (Days)	Concetration of Sulfachloropyridazina (ng/g)	Average	LN (concent)	SD LN	95% of confidence LN+(1,6449+DSLN)	LN LOD (10 ng/g)	LN MRL edible tissue(100 ng/g)
1	7	1	16	4936.646		8.50	0.38	10.53	1.00	4.61
1	7	2	16	2064.029		7.63	0.38	9.66	1.00	4.61
1	7	3	16	1973.042		7.59	0.38	9.61	1.00	4.61
1	7	4	16	1830.061	2858.78	7.51	0.38	9.53	1.00	4.61
1	7	5	16	4026.768		8.30	0.38	10.32	1.00	4.61
1	7	6	16	2752.938		7.92	0.38	9.94	1.00	4.61
1	7	7	16	2427.981		7.79	0.38	9.82	1.00	4.61
2	14	1	23	721.427		6.58	0.50	8.73	1.00	4.61
2	14	2	23	353.469		5.87	0.50	8.02	1.00	4.61
2	14	3	23	541.935	438.89	6.30	0.50	8.44	1.00	4.61
2	14	4	23	299.622		5.70	0.50	7.85	1.00	4.61
2	14	5	23	536.949		6.29	0.50	8.43	1.00	4.61
2	14	6	23	179.961		5.19	0.50	7.34	1.00	4.61
3	21	1	30	89.434		4.49	0.48	6.62	1.00	4.61
3	21	2	30	155.636		5.05	0.48	7.18	1.00	4.61
3	21	3	30	369.538		5.91	0.48	8.04	1.00	4.61
3	21	4	30	120.982	183.39	4.80	0.48	6.92	1.00	4.61
3	21	5	30	130.542		4.87	0.48	7.00	1.00	4.61
3	21	6	30	264.380		5.58	0.48	7.71	1.00	4.61
3	21	7	30	153.246		5.03	0.48	7.16	1.00	4.61
4	32	1	43	22.573		3.12	0.95	5.71	1.00	4.61
4	32	2	43	32.656		3.49	0.95	6.08	1.00	4.61
4	32	3	43	26.299		3.27	0.95	5.86	1.00	4.61
4	32	4	43	35.944	18.92	3.58	0.95	6.18	1.00	4.61
4	32	5	43	5.000		1.61	0.95	4.20	1.00	4.61
4	32	6	43	5.000		1.61	0.95	4.20	1.00	4.61
4	32	7	43	5.000		1.61	0.95	4.20	1.00	4.61

LOD	10
LOQ	14.6

Intercepto	9.205
Pendiente	-0.205
R2	0.908

Intercepto	11.024
Pendiente 95%	-0.183
R2 95%	0.890
WDT	54.705

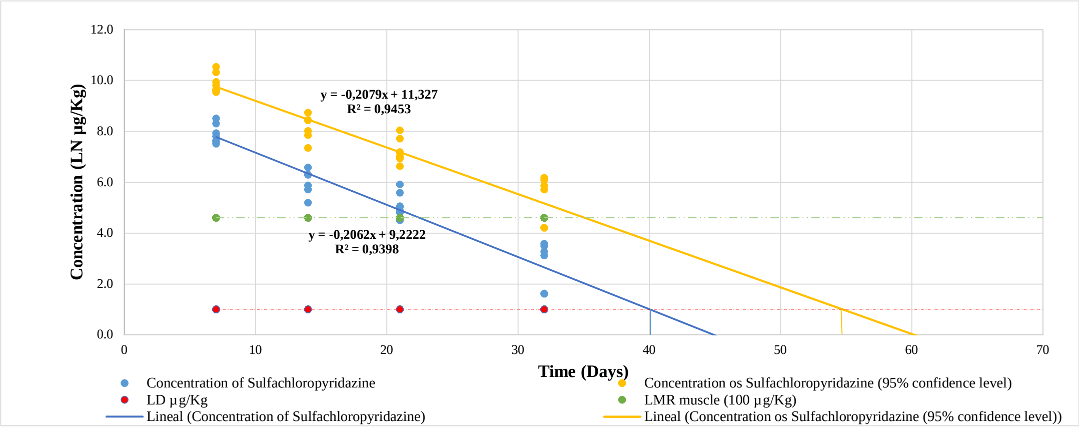

Supplement: S1 Table — Data of concentrations of sulfachloropyridazine residues in feathers samples, for each sampling point during depletion determination. (PDF) [file pone.0200206.s004.pdf]
